# Isolation, Structural Characterization and Antidiabetic Activity of New Diketopiperazine Alkaloids from Mangrove Endophytic Fungus *Aspergillus* sp. 16-5c

**DOI:** 10.3390/md19070402

**Published:** 2021-07-20

**Authors:** Geting Ye, Cuiying Huang, Jialin Li, Tao Chen, Jing Tang, Wenbin Liu, Yuhua Long

**Affiliations:** Guangzhou Key Laboratory of Analytical Chemistry for Biomedicine, School of Chemistry, South China Normal University, Guangzhou 510006, China; yegeting@m.scnu.edu.cn (G.Y.); huangcuiying@m.scnu.edu.cn (C.H.); jialinli@m.scnu.edu.cn (J.L.); ct2020@m.scnu.edu.cn (T.C.); tangjing2020@m.scnu.edu.cn (J.T.); liuwenbin@m.scnu.edu.cn (W.L.)

**Keywords:** diketopiperazine alkaloids, mangrove fungi, antidiabetic activity, α-glucosidase inhibition, PTP1B inhibition

## Abstract

Six new DIKETOPIPERAZINE alkaloids aspergiamides A–F (**1**–**6**), together with ten known alkaloids (**7**–**16**), were isolated from the mangrove endophytic fungus *Aspergillus* sp. 16-5c. The structures of the new compounds were elucidated based on 1D/2D NMR spectroscopic and HR-ESIMS data analyses. The absolute configurations of aspergiamides A-F were established based on the experimental and calculated ECD data. All the compounds were evaluated for the antidiabetic activity against α-glucosidase and PTP1B enzyme. The bioassay results disclosed compounds **1** and **9** exhibited significant α-glucosidase inhibitory with IC_50_ values of 18.2 and 7.6 μM, respectively; compounds **3**, **10**, **11,** and **15** exhibited moderate α-glucosidase inhibition with IC_50_ values ranging from 40.7 to 83.9 μM; while no compounds showed obvious PTP1B enzyme inhibition activity.

## 1. Introduction

Type 2 diabetes mellitus (T2DM) is a metabolic disorder disease and is characterized by an imbalance of blood glucose levels due to defects in insulin secretion. Increased blood glucose levels in T2DM may induce hypertension, atherosclerosis, and other metabolic disorder diseases [1]. It is estimated that 425 million people have diabetes, and about 90% account for type 2 diabetes. According to the World Health Organization (WHO), diabetes will reach the seventh cause of death by 2030 [2]. Although many antidiabetic drugs with different mechanisms can be available from the market, the inevitable side effects such as weight gain, liver damage, and allergic reactions hinder their application [3]. PTP1B is an intracellular non-transmembrane enzyme, which plays a major negative regulator role in insulin function by dephosphorylation of tyrosine-phosphorylated proteins [4]. Studies have shown that overexpression of protein tyrosine phosphatase 1B (PTP1B) can lead to the dephosphorylation of insulin receptors and insulin receptor substrates, resulting in insulin resistance and ultimately inactivating the entire insulin signaling pathway. Therefore, PTP1B inhibitors have attracted particular attention as potential therapeutic agents against diabetes [5]. In addition, α-glucosidase, a traditional antidiabetic target responsible for the hydrolysis of polysaccharides to monosaccharides, is still considered an important direction to discover new chemical entities for the treatment of non-insulin-dependent T2DM [6]. Therefore, the development of PTP1B and α-glucosidase inhibitors could be a promising strategy in discovering novel antidiabetic drug candidates.

Diketopiperazine (DKP) alkaloids are important secondary metabolites of microbes. Among these alkaloids, the diketopiperazine moiety is biosynthesized from different amino acids and formed as the smallest cyclic dipeptide skeleton. Diketopiperazine alkaloids have many important biological activities, such as antiviral, anti-tumor, antibacterial, and so on [7,8]. Indole diketopiperazine alkaloids belong to the subclass of DKPs are the condensation products of a complete tryptophan with a second amino acid-like l-tryptophan, l-proline, l-phenylalanine, or l-leucine [9]. Indole diketopiperazine alkaloids have attracted widespread attention not only because of their unique structure but also because of the wide range of biological activities, such as antiviral [10], anticancer [11], antioxidant [12], and insecticidal activities [13]. Marine fungi are important sources of diketopiperazine alkaloids. According to statistics, between 2000 and 2019, as many as 155 indole diketopiperazine alkaloids were identified from marine fungi alone [14]. 

**Figure 1:**
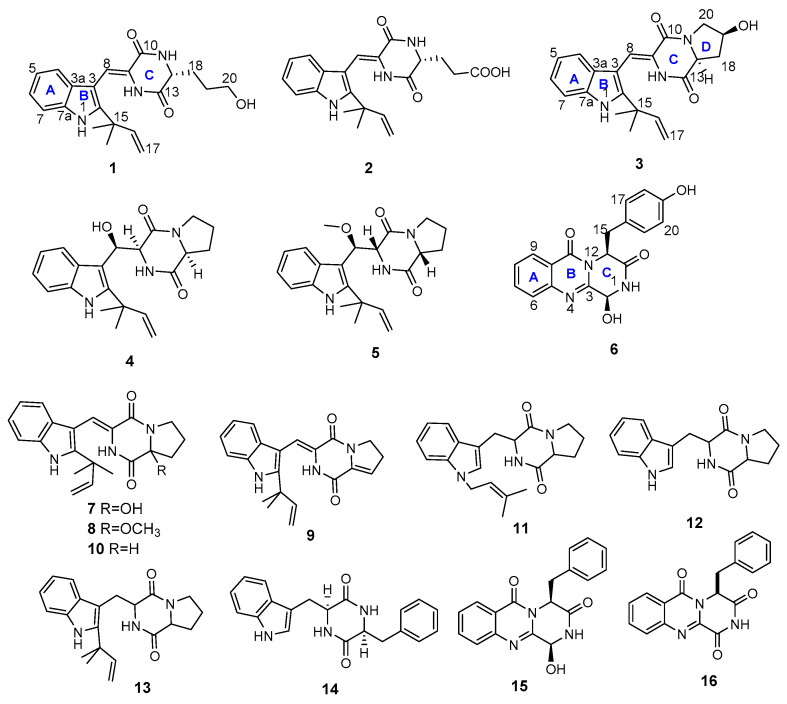
Chemical structures of compounds **1**–**16.**

As part of our aims for exploring biological active natural products [15], the secondary metabolites from fungus *Aspergillus* sp. 16-5c were investigated. Chemical investigation yielded six new diketopiperazine alkaloids, including five indole diketopiperazine alkaloids, aspergiamides A–E (**1**–**5**) and one 4-quinazolinone like diketopiperazine alkaloids aspergiamide F (**6**), together with ten known alkaloids (**7**–**16**) from the culture extracts of the fungus 16-5c (Figure 1). These compounds were evaluated the antidiabetic potential by screening the enzyme inhibition of α-glucosidase and PTP1B. Herein, we describe the isolation and structure elucidation of the new compounds, as well as their bioactivities.

## 2. Results and Discussion

### Structural Elucidation

**Figure 2:**
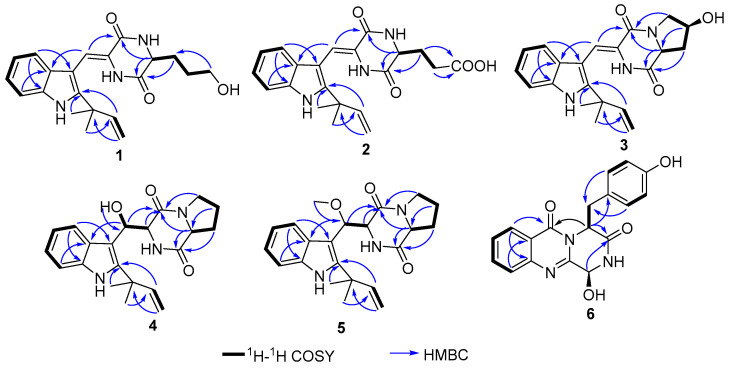
Key HMBC and 1H-1H COSY correlations of **1**–**6**.

**Figure 3:**
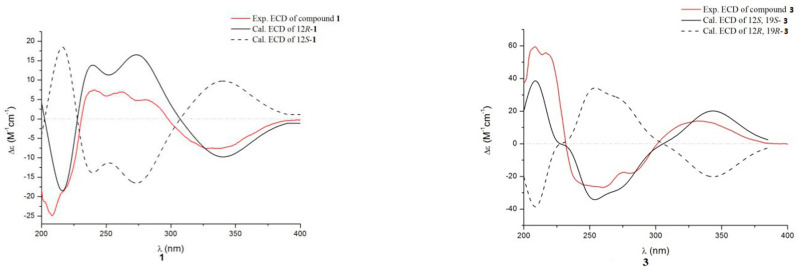
Comparison between the experimental and calculated ECD spectra of **1** and **3**.

Compound **1** was obtained as a pale yellow powder. The molecular formula was established as C_21_H_25_O_3_N_3_ according to the HRESIMS at *m*/*z* 366.1827 [M − H]^−^ (Calcd 366.1823 for C_21_H_24_O_3_N_3_), indicating 11 degrees of unsaturation. The ^13^C NMR and HMQC spectra (Table 1, Figures S3 and S4) displayed 21 carbon signals, consisting of two methyls, four methylenes (one olefinic carbon), seven methines (including four aromatic methines), and eight quaternary carbons (including two amide carbons, three olefinic carbons). The ^1^H NMR (Table 1) spectrum of **1** revealed a set of adjacent aromatic protons at δ_H_ 7.08 (m, 1H), 7.13 (m, 1H), 7.24 (d, J = 7.9 Hz, 1H), 7.43 (d, J = 8.0 Hz, 1H), indicating the occurrence of two *ortho*-substituted benzene ring. Four olefinic protons resonances at δ_H_ 5.11 (m, 2H), 6.11 (dd, J = 17.3, 10.6 Hz, 1H), 7.22 (s, 1H), two methyls at δ_H_ 1.54 (s, 3H) and 1.55 (s, 3H), three aliphatic methylenes at δ_H_ 2.03–1.67 (m, 4H) and 3.63 (t, J = 6.3 Hz, 2H), one aliphatic methine at δ_H_ 4.21 (t, J = 6.3 Hz, 1H) were also recorded in this spectrum. The ^1^H-^1^H COSY spectrum of 1 showed cross-peaks that connect the aromatic protons from H-4 (δ_H_ 7.24) through H-7 (δ_H_ 7.43), while HMBC correlations from H-4 to C-7a (δ_C_ 136.8)/C-3 (δ_C_ 104.3), from H-7 to C-3a (δ_C_ 127.1) suggesting an indole ring (Figure 2). The HMBC correlations from H-12 (δ_H_ 4.21) to C-10 (δ_C_ 162.5)/C-13 (δ_C_ 168.1) and correlations from H-17 (δ_H_ 5.11) to C-15 (δ_C_ 40.5), from H3-15a/15b (δ_H_ 1.55/1.54) to C-16 (δ_C_ 146.0) combined with the ^1^H-^1^H COSY correlation between H-16 (δ_H_ 6.11) and H-17, suggesting a diketopiperazine fragment and a prenyl unit, respectively. The indole ring was further connected with the diketopiperazine and prenyl group to form a 2,3-disubstituted indole diketopiperazine substructure consistent with HMBC correlations from H3-15a/15b to C-2 (δ_C_ 146.2), H-8 (δ_H_ 7.22) to C-10/C-3/C-3a. All the above NMR data suggested the structure of **1** was similar to the known compound neoechinulin A [16], with the main difference at the presence of an –CH2-CH2-CH2OH moiety in **1**. The ^1^H-^1^H COSY correlation of H-18 (δ_H_ 1.95–2.03)/H-19 (δ_H_ 1.67–1.74)/H-20 (δ_H_ 3.63) suggested the existence of –CH_2_-CH_2_-CH_2_– fragment. The chemical shift of C-20 (δ_C/H_ 62.5/3.63), combined with HMBC correlation from H-20 to C-18, indicated a hydroxyl was located at C-20 to form a –CH_2_-CH_2_-CH_2_OH moiety. Moreover, key HMBC correlations from H-19 to C-12 suggested the moiety was connected with C-12, as shown in Figure 1. The *Z*-geometry of the Δ8 double bond was identified by the lack of NOE effect between H-8 and NH-14 [17]. The absolute configuration of compound **1** was determined by the quantum chemical calculation study for electronic circular dichroism (ECD). Consequently, the calculated ECD for 12*R*-**1** is consistent with the experimental ECD of **1** (Figure 3), which indicated the absolute configuration of **1** as 12*R*. Thus, the structure of compound **1** was identified and named aspergiamide A.

Compound **2** was isolated as a white powder and assigned a molecular formula of C_21_H_23_O_4_N_3_ (12 degrees of unsaturation) based on the HRESIMS and 1D NMR (Figures S8–S14). The ^13^C NMR (Table 1) displayed 21 carbon signals, consisting of two methyls, three methylenes (one olefinic carbon), seven methines, and nine quaternary carbons (including one carboxyl carbonyl carbon, two amide carbonyl carbons). The ^1^H NMR spectrum (Table 1) of **2** revealed a set of adjacent aromatic protons at δ_H_ 7.08 (m, 1H), 7.13 (m, 1H), 7.26 (d, J = 7.8 Hz, 1H), 7.42 (d, J = 8.0 Hz, 1H), indicating the occurrence of two ortho-substituted benzene ring. Four olefinic protons resonances at δ_H_ 5.11 (m, 2H), 6.11 (dd, J = 17.3, 10.6 Hz, 1H), 7.22 (s, 1H), two methyls at δ_H_ 1.54 (s, 3H) and 1.55 (s, 3H), two aliphatic methylenes at δ_H_ 2.21 (m, 2H) and 2.48 (m, 2H), one aliphatic methine at δ_H_ 4.25 (t, J = 5.6 Hz, 1H) were also recorded. Cumulative analyses of 1D and 2D NMR spectroscopic data revealed that **2** possessed a similar planar structure as that of **1**. A major difference was the replacement of a hydroxymethylene group in **1** (δ_C_ 62.5) by a carboxyl group in **2** (δ_C_ 176.3), and the chemical shift of H-19 downfield from 1.67 in **1** to 2.84 in **2** due to the deshielding effect of the carboxyl group, indicating that compound **2** is the oxidation product of **1**. Then, the planar structure of **2** was further verified by analyzing 2D NMR, as shown in Figure 2. The ^1^H-^1^H COSY correlation of H-18 (δ_H_ 2.21)/H-19 (δ_H_ 2.48) suggested the existence of –CH_2_-CH_2_– fragment. The chemical shift of C-20 (δ_C_ 176.3), combined with HMBC correlation from H-18 to C-20 and from H-19 to C-20, indicated a carboxyl group was located at C-20 to form a –CH_2_-CH_2_-COOH moiety. Moreover, key HMBC correlations from H-19 to C-12 (δ_C_ 56.1) suggested the moiety was connected with C-12, as shown in Figure 1. Δ8 double bond was identified as *Z* configuration by the lack of NOE effect between H-8 and NH-14 [17]. Furthermore, the absolute configuration of C-12 was determined to be R based on the same negative sign of its specific rotation ($\;{\left[\alpha \right]}_{D}^{25}-154.8{}^{\circ},\;c\;0.1,\;\mathrm{MeOH}$), as that of compound **1** (${\left[\alpha \right]}_{D}^{25}-171.4{}^{\circ},\;c\;0.1,\;\mathrm{MeOH}$)). Therefore, the structure of compound **2** was identified and named aspergiamide B. 

**Figure 4:**
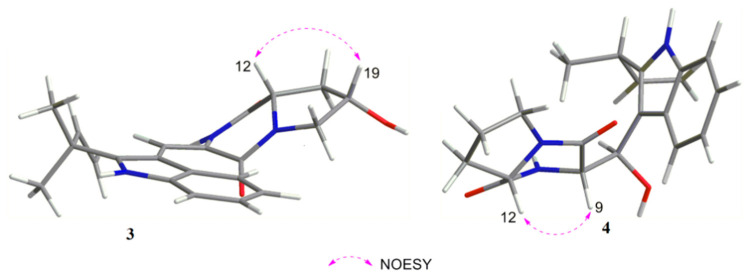
Key NOESY correlations of **3** and **4.**

Compound **3** was isolated as a pale yellow powder. It gave a molecular formula of C_21_H_23_O_3_N_3_, as established by the HRESIM and NMR data, showing 12 degrees of unsaturation. The ^13^C NMR and HSQC spectra displayed 21 carbon signals, consisting of two methyls, three methylenes (one olefinic carbon), seven methines, and nine quaternary carbons (including one carboxyl carbonyl carbon, two amide carbonyl carbons). Analysis of the ^1^H NMR spectrum of **3** revealed a set of adjacent aromatic protons at δ_H_ 7.07 (m), 7.12 (m), 7.31 (d, J = 7.9), 7.41, (d, J = 8.0), indicating the occurrence of two ortho-substituted benzene ring. Four olefinic protons at δ_H_ 5.11 (m), 6.11 (dd, J = 17.2, 10.8), 7.23 (s), two methylenes at δH 2.11 (m, 1H), 2.34 (dd, J = 13.0, 5.9), 3.53 (d, J = 13.2), 3.92 (dd, J = 13.2, 4.9), two aliphatic methines at δ_H_ 4.51 (m) and 4.72 (dd, J = 11.7, 5.9). All the above data were indicative of a prenyl containing 2, 3-disubstituted indole diketopiperazine skeleton. The ^1^H-^1^H COSY correlation of H-18 (δ_H_ 2.11, 2.34)/H-19 (δ_H_ 4.51)/H-20 (δ_H_ 3.53, 3.92) suggested an aliphatic fragment of –CH_2_-CH-CH_2_–, combined with key HMBC correlations from H-19 to C-12 (δ_C_ 58.7), from H-18 to C-13 (δ_C_ 168.2), from H-20 to C-10 (δ_C_ 160.9) suggested the aliphatic fragment was connected with diketopiperazine ring at C-12 and N-11 to form a five-member ring. The chemical shift of C-19 (δ_C/H_ 68.6, 4.51; CH), together with the HRESIMS result, indicated a hydroxyl was connected with C-19. The geometry of the Δ8 double bond was Z configuration according to the chemical shift of H-8 (δ_H_ 7.23) together with the lack of NOE effect between H-8 and NH-14 [17]. The NOESY correlation observed between H-12 and H-19 indicated that H-12 and H-19 were on the same face of the molecule (Figure 4), implying the existence of two possible relative configurations. Therefore, compound **3** had only one pair of enantiomers (12S, 19S-**3** or 12R, 19R-**3**). The absolute configuration of **3** was determined by ECD calculation (Figure 3). The calculated data of 12*S*, 19*S*-**3** showed good agreement with the experimental ECD of **3**. Thus, the structure of **3** was assigned and named aspergiamide C.

**Figure 5:**
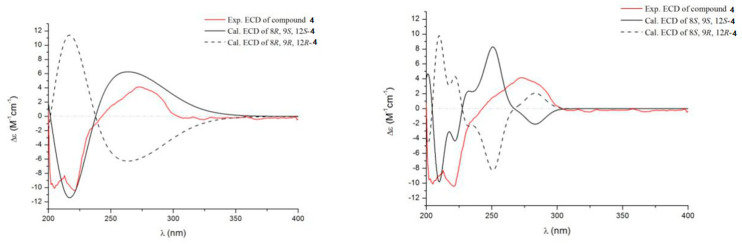
Comparison between the experimental and calculated ECD spectra of **4**.

Compound **4** was obtained as a pale yellow powder with a molecular formula C_21_H_25_O_3_N_3_ based on HRESIMS (Figure S22), indicating 11 degrees of unsaturation. The ^13^C NMR and HMQC spectra (Table 2) showed signals for two amide carbons, four aromatic carbons, two olefinic carbons, three methylenes, three oxygen- or nitrogen-bonded methines, two methyls, and five quaternary carbons, combined with a set of adjacent aromatic protons at δ_H_ 7.07 (m), 7.12 (m), 7.31 (d, J = 7.9), 7.41, (d, J = 8.0) in ^1^H NMR spectrum, suggesting high similarity to the known compound brevianamide W (**9**) [18]. However, the degree of unsaturation for **9** was 12, one more than that of **4**. Moreover, two olefinic carbons [δ_C_ 112.5 (CH), 126.2 (C)] in **9** were replaced by two oxygen- or nitrogen-bonded methines [δ_C_ 68.9 (CH), 62.7 (CH)] in **4** according to the ^13^C NMR and HMQC spectra. The ^1^H-^1^H COSY correlations between OH-8 (δ_H_ 5.33)/H-8 (δ_H_ 5.30)/H-9 (δ_H_ 4.0) indicated a -CH-CH-OH unit. The key HMBC (Table 2) correlations from OH-8 to C-8 (δ_C_ 68.9)/C-9 (δ_C_ 62.7)/C-3 (δ_C_ 111.1), from H-8 to C-10 (δ_C_ 164.4) /C-3a (δ_C_ 127.3), combined with the chemical shift of C-8 [δ_C/H_ 68.9/5.30 (dd, J = 6.2, 3.5)], suggesting that Δ8 double bond of **9** was reduced, and a proton at C-8 was replaced by a hydroxyl group. Thus, the planar structure of **4** was shown in Figure 1. The correlation between H-9 and H-12 in the NOESY spectrum (Figure 3) suggested that they were located on the same side. Therefore, the ECD spectra of the four possible absolute configurations (8*R*, 9*S*, 12*S*-**4** or 8*R*, 9*R*, 12*R*-**4**; 8*S*, 9*S*, 12*S*-**4** or 8*S*, 9*R*, 12*R*-**4**) were calculated (Figure 5). The experimental ECD curve was consistent with the calculated configurations of 8*R*, 9*S*, 12*S*-**4**. Thus, the structure of compound **4** was identified and named aspergiamide D.

**Figure 6:**
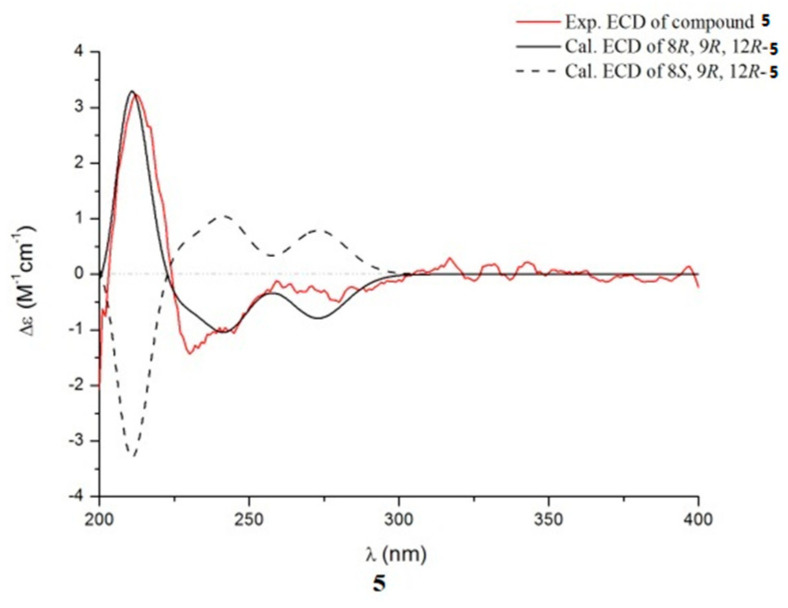
Comparison between the experimental and calculated ECD spectra of **5.**

Compound **5** was isolated as a light yellow powder. The molecular formula was established as C_22_H_27_O_3_N_3_ according to the HRESIMS, showing 11 degrees of unsaturation. The ^13^C NMR and HMQC spectra (Table 2) showed signals for seven quaternary carbons, four methylenes, eight methines, and two methyls, together with a set of adjacent aromatic protons at δ_H_ 7.03 (m), 7.09 (m), 7.40 (d, J = 8.1), 7.74 (d, J = 8.0) in ^1^H NMR spectrum, indicating high similarity to **4**, except one more methoxyl signal at δ_H_ 3.17 (s, 3H) and δ_C_ 56.9 was observed in the 1D NMR data spectra of **5**. HMBC correlations from OCH_3_-8 (δ_H_ 3.17) to C-8 (δ_C_ 77.8), from H-8 to C-3a (δ_C_ 128.7)/C-10 (δ_C_ 165.8) indicated the methoxyl was located at C-8. The absolute configuration of **5** was tentatively assigned as 9*R* and 12*R* by the negative sign of its specific rotation ($\;{\left[\alpha \right]}_{D}^{25}-22.1{}^{\circ},\;c\;0.1,\;\mathrm{MeOH}$) which was comparable to that of rubrumline F (${\left[\alpha \right]}_{D}^{25}-22.7{}^{\circ}$) [19]. Based on the above configurations of C-9 and C-12, compound **5** gave only one pair of enantiomers (8*R*, 9*R*, 12*R*-**5** or 8*S*, 9*R*, 12*R*-**5**). Consequently, the calculated ECD (Figure 6) for 8*R*, 9*R*, 12*R*-**5** showed good agreement with the experimental ECD of **5**. Thus, the structure of **5** was identified and named aspergiamide E.

**Figure 7:**
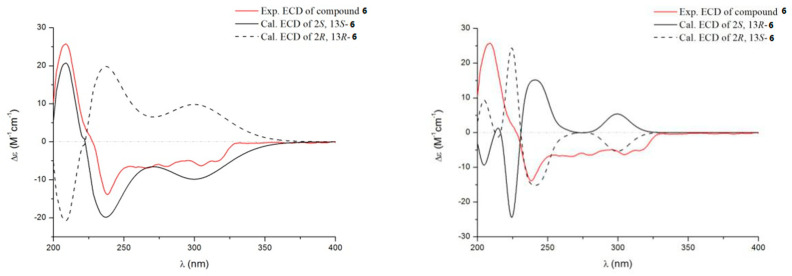
Comparison between the experimental and calculated ECD spectra of **6**.

Compound **6** was obtained as a white powder. It gave a molecular formula of C_18_H_15_O_4_N_3_, as established by HRESIMS and NMR data, indicating 13 degrees of unsaturation. The ^13^C NMR spectrum of **6** showed one methylene signal, ten methines (including eight aromatic methines and one oxygenated methine), and seven quaternary carbons. Analysis of the ^1^H NMR spectrum of **6** (Table 2) revealed a set of adjacent aromatic protons at 7.56, (dd, J = 4.0, 11.1), 7.73 (d, J = 8.1), 7.84 (m), and 8.14 (dd, J = 1.1, 8.0), and a set of opposite aromatic protons at δ_H_ 6.64 (d, J = 8.4, 2H), 7.04 (d, J = 8.4, 2H), indicating the occurrence of one ortho-substituted benzene ring and one para-substituted benzene ring. The ^1^H-^1^H COSY spectrum of **6** showed cross-peaks that connect the aromatic protons from H-6 (δ_H_ 7.73) through H-9 (δ_H_ 8.14), while HMBC correlations from H-6 to C-10 (δ_C_ 121.6), from H-9 to C-5 (δ_C_ 128.2)/C-11 (δ_C_ 161.8) suggested a quinazoline ring. The quinazoline ring was further fused with a pyrazine to form a tricyclic pyrazinoquinazolinedione [20], which was consistent with HMBC correlations from H-13 (δ_H_ 5.37) to C-11/C-3 (δ_C_ 151.4)/C-14 (δ_C_ 171.8), from H-2 (δ_H_ 5.71) to C-3/C-14. Moreover, the linkage between the para-substituted benzene ring and pyrazinoquinazolinedione moiety by C-15 was deduced based on HMBC correlations from H-13 to C-6, from H-17/H-19 to C-15 (Figure 2). The planar structure of **6** showed a close similarity to that of brevianamide M (**15**) [21], with the main difference being one more hydroxyl in **6**, which was deduced based on the lack of an aromatic proton, and the downfield shift of C-19 (δ_C_ 157.6) in 1D NMR spectra. Compound **6** has two chiral centers (C-2/C-13); therefore, the ECD spectra of four possible absolute configurations (2*S*,13*S*-**6** or 2*R*,13*R*-**6**; 2*S*,13*R*-**6** or 2*R*,13*S*-**6**) were calculated as shown in Figure 7. The experimental ECD data was consistent with the calculated configurations of 2*S*,13*S*-**6**. Thus, the structure of compound **6** was identified and named aspergiamide F.

In addition to the isolation of the above new compounds, ten known analogues including brevianamide Q (**7**) [22], brevianamide R (**8**) [22], brevianamide K (**9**) [21], brevianamide W (**10**) [18], *N*-Prenyl-cyclo-l-tryptophyl-l-proline (**11**) [23], brevianamide F (**12**) [24], epi-deoxybrevianamide E (**13**) [25], cyclo-(tryptophyl-phenylalanyl) (**14**) [26], brevianamide M (**15**) [21] and brevianamide N (**16**) [21] were isolated and identified from this fungus. Their structures were determined by comparing their NMR and MS data with the reported in literatures.

Investigation on the biological activities of prenylated DKP alkaloids disclosed antioxidant activity in the literature [27,28]. It is reported that excessive accumulation of free radical and synchronous reduced the antioxidant defense mechanisms and can induce complications of diabetes mellitus [15]. In order to find potent antidiabetic natural products, all the compounds (**1**–**16**) were screened for the enzyme inhibitory activities against α-glucosidase and PTP1B (Table 3). Compared with the positive control acarbose (408 μM), all of the tested compounds, except compounds **5**, **12**, and **13**, showed good to moderate inhibitory activity to α-glucosidase. Especially, compounds **1** and **9** exhibited significant α-glucosidase inhibitory with IC_50_ values of 18.2 and 7.6 μM, respectively. Compounds **3**, **10**, **11,** and **15** showed moderate α-glucosidase inhibition with IC_50_ values ranging from 40.7 to 83.9 μM. However, these compounds have no obvious PTP1B inhibition at a concentration of 100 μg/mL (Table 3). 

## 3. Materials and Methods

### 3.1. General Experimental Procedures

HRESIMS data were recorded with a Finnigan LTQ-Orbitrap Elite (Thermo Fisher, Waltham, MA, USA). NMR spectra were reported by Bruker AVANCE NEO 600 MHz spectrometer (Bruker BioSpin, Fällanden, Switzerland). CD spectrum was obtained on a Chirascan spectropolarimeter (Applied Photophysics, Leatherhead, UK). Optical rotation was determined on an MCP 500 (Anton Paar, Austria). UV spectra were obtained on a PERSEE TU-1990 spectrophotometer. Sephadex LH-20 (25−100 μm; GE Healthcare, Sweden) and silica gel (200−300 mesh; Qingdao Shenghai Chemical Co. Ltd., Qingdao, China) were used for CC. Thin-layer chromatography (TLC) was detected on the Silica gel GF254 plate (Qingdao Marine Chemical Ltd., Qingdao, China).

### 3.2. Fungal Material

The fungus (16-5c) used in this research was isolated from the leaves of S. apetala, a mangrove plant that was collected from Hainan Island, China. The fungus was identified as *Aspergillus* sp. by the ITS region (deposited in GenBank, accession no JX993829) [29].

### 3.3. Fermentation, Extraction, and Isolation

The fungus *Aspergillus* sp. 16-5c was fermented on solid autoclaved rice medium using eighty 1 L Erlenmeyer flasks; each contained 50 g rice and 50 mL 0.3% saline water, culturing in room temperature under static condition for 28 days. The mycelia and solid rice medium were extracted with methanol three times. The organic solvents were evaporated under reduced pressure; we obtained 110 g of organic extract. The extract was isolated by column chromatography over silica gel eluting with a gradient of petroleum ether/ethyl acetate from 1/0 to 0/1 to afford four fractions (Fractions 1–5). Fraction 3 (253 mg) was applied to silica gel CC and purified by Sephadex LH-20 CC to obtain compounds **7**–**10**, **13**, and **16**. Fraction 4 (203 mg) was applied to column chromatography over silica gel, eluting with CH2Cl2/MeOH (100:1), and then further purified by Sephadex LH-20 CC eluted with MeOH to give compounds **1**–**6**, **11**–**12**, and **14**–**15**.

Aspergiamide A: pale yellow powder; ${\left[\alpha \right]}_{D}^{25}-171.4{}^{\circ}\;\left(c\;0.1,\;\mathrm{MeOH}\right)$; (c 0.1,MeOH)″; UV (MeOH) λmax (logε) 222 (3.30), 284 (0.90), 337 (1.05) nm; ECD λext (Δε) (MeOH), 220 (−18.75), 240 (+13.50), 275 (+16.51), 340 (−10.18) nm; HRESIMS at *m*/*z* 366.1827 [M − H]^−^ (calcd for C_21_H_24_N_3_O_3_, 366.1823). ^1^H and ^13^C NMR see Table 1.

Aspergiamide B: white powder; ${\left[\alpha \right]}_{D}^{25}-154.8{}^{\circ}\;\left(c\;0.1,\;\mathrm{MeOH}\right);$ (c 0.1,MeOH)″; UV (MeOH) λmax (log ε) 224 (2.16), 284 (0.59),338 (0.76) nm; HRESIMS at *m*/*z* 382.1766 [M + H]^+^ (calcd for C_21_H_24_N_3_O_4_, 382.1761). ^1^H and ^13^C NMR see Table 1.

Aspergiamide C: pale yellow powder; ${\left[\alpha \right]}_{D}^{25}+64.5{}^{\circ}\;\left(c\;0.1,\;\mathrm{MeOH}\right)$; (c 0.1,MeOH)″; UV (MeOH) λmax (log ε) 225 (2.36), 284 (0.671), 338 (0.90) nm; ECD λext (Δε) (MeOH), 210 (+38.95), 254 (35.40), 345 (+19.85) nm.; HRESIMS at *m*/*z* 364.1670 [M − H]^−^ (calcd for C_21_H_22_O_3_N_3_, 364.1666). ^1^H and ^13^C NMR see Table 1.

Aspergiamide D: pale yellow powder; ${\left[\alpha \right]}_{D}^{25}+78.5{}^{\circ}\;\left(c\;0.1,\;\mathrm{MeOH}\right)$; (c 0.1,MeOH)″; UV (MeOH) λmax (log ε) 213 (1.63) nm; ECD λext (Δε) (MeOH), 217.5 (−11.51), 263 (+6.15) nm; HRESIMS at *m*/*z* 366.1828 [M − H]^−^ (calcd for C_21_H_24_O_3_N_3_, 366.1823). ^1^H and ^13^C NMR see Table 2.

Aspergiamide E: light yellow powder; ${\left[\alpha \right]}_{D}^{25}-22.1{}^{\circ}\;\left(c\;0.1,\;\mathrm{MeOH}\right)$; (c 0.1,MeOH)″; UV (MeOH) λmax (log ε) 213 (2.13) nm; ECD λext (Δε) (MeOH), 210 (−3.45), 245 (−1.12), 260 (−0.36), 271 (−0.78) nm; HRESIMS at *m*/*z* 404.1946 [M + Na]^+^ (calcd for C_22_H_27_O_3_N_3_Na, 404.1944). ^1^H and ^13^C NMR see Table 2.

Aspergiamide F: white powder; ${\left[\alpha \right]}_{D}^{25}-136.8{}^{\circ}\;\left(c\;0.1,\;\mathrm{MeOH}\right)$; (c 0.1,MeOH)″; UV (MeOH) λmax (log ε) 213 (2.13), 261 (0.67) nm; ECD λext (Δε) (MeOH), 210 (+20.12), 237.3 (−20.05), 260 (−6.58), 305 (−9.85) nm; HRESIMS at *m*/*z* 336.0993 [M − H]^−^ (calcd for C_18_H_15_O_4_N_3_, 336.0989). ^1^H and ^13^C NMR see Table 2.

### 3.4. Antidiabetic Bioassays

The α-glucosidase inhibition assay was performed in a 96-well microplate reader method as described in a previous report [24]. The α-glucosidase assay was performed at 37 °C with a final volume of 200 μL. For each well, 10 μL samples (dissolved in DMSO), followed by the addition of 50 μL phosphate buffer (pH 6.86), 20 μL 0.2 U/mL α-glucosidase solutions and incubated for 5 min. 20 μL substrate p-nitrophenyl-α-d-glucopyranoside (p-NPG) was added to each well to start the reaction. The reaction was incubated at 37 °C for 15 min and stopped by the addition of 50 μL 0.4 M Na_2_CO_3_. The α-Glucosidase activity was determined by measuring the release of p-nitrophenol from p-NPG at 405 nm.

PTP1B inhibitory activity was determined by measuring the enzymatic hydrolysis rate of p-nitrophenyl phosphate (p-NPP) as reported in the study [30]. This assay was performed at 37 °C with a final volume of 100 μL, using a Tris-HCl buffer (25 mM Tris-HCl, 1 mM EDTA, 1 mM DTT, pH 7.5). Samples were dissolved in 10 μL DMSO followed by the addition of 30 μL buffer, 40 μL 0.075 U/μL PTP1B stock solution, and then added 20 μL 22.26 μg/μL p-NPP solution to start the reaction. After 30 min, the reaction ended by adding 50 μL 2.5 M NaOH solution. The absorbance was measured at 405 nm.

The purity of the tested compounds by HPLC analysis for bioassay was 96% for **1**, 96% for **2**, 97% for **3**, 98% for **4**, 96% for **5,** 98% for **6**, 98% for **7**, 98% for **8**, 98% for **9**, 99% for 10, 96% for **11**, 96% for **12**, 97% for **13**, 98% for **14**, 97% for **15**, and 95% for **16**.

### 3.5. ECD Calculation

Conformational searches were carried out by means of the Spartań14 software using the Molecular Merck force field (MMFF). All density functional theory (DFT) and time-dependent (TD)-DFT calculations were performed with Gaussian 09 program. Conformers within a 10 kcal/mol energy window were generated and optimized by DFT calculations at the B3LYP/6-31+G (d, p) level. Conformers with a Boltzmann distribution of over 5% were chosen for ECD calculations by the TD-DFT method at the B3LYP/6-31+G (d, p) level [31]. The polarizable continuum model for MeOH was used. The calculated ECD curves were generated using the SpecDis 3.0 (University of Würzburg, Würzburg, Germany) and Origin Pro 8.0 (Origin Lab, Ltd., Northampton, MA, USA) from dipole-length rotational strengths by applying Gaussian band shapes with sigma = 0.30 eV.

## 4. Conclusions

In conclusion, chemical investigation of the mangrove endophytic fungus *Aspergillus* sp. 16-5c led to isolation and identification of six new alkaloids, aspergiamides A–F (**1**–**6**), with ten known analogs (**7**–**16**). The potential of DKPs alkaloids as antidiabetic agents was evaluated by α-glucosidase inhibition and PTP1B inhibition assay. The results of the α-glucosidase inhibition assay showed compounds **1** and **9** exhibited significant α-glucosidase inhibitory with IC_50_ values of 18.2 and 7.6 μM, respectively. Meanwhile, all the tested DKPs compounds showed no obvious PTP1B inhibition at 100 μg/mL concentration. These outcomes expanded the chemical and biological diversity of DKPs alkaloids and may provide new molecules for antidiabetic drug discovery.

## Figures and Tables

**Table 1:** ^1^H (600 MHz) and ^13^C NMR (150 MHz) data for compounds **1**–**3**.

Position	1 ^a^		2 ^a^		3 ^a^	
	δ_C_	δ_H_ (J/Hz)	δ_C_	δ_H_ (J/Hz)	δ_C_	δ_H_ (J/Hz)
2	146.2, C		146.2, C		146.2, C	
3	104.3, C		104.3, C		104.6, C	
3a	127.1, C		127.3, C		126.0, C	
4	119.9, CH	7.24, d (7.9)	120.0, CH	7.26, d (7.8)	112.5, CH	7.41, d (8.0)
5	121.2, CH	7.08, m	121.2, CH	7.08, m	122.4, CH	7.12, m
6	122.5, CH	7.13, m	122.7, CH	7.13, m	121.1, CH	7.07, m
7	112.6, CH	7.43, d (8.0)	112.6, CH	7.42, d (8.0)	120.2, CH	7.31, d (7.9)
7a	136.8, C		136.9, C		136.7, C	
8	114.5, CH	7.22, s	114.7, CH	7.22, s	114.6, CH	7.23, s
9	124.6, C		124.5, C		127.3, C	
10	162.5, C		162.5, C		160.9, C	
12	56.8, CH	4.21, t (5.4)	56.1, CH	4.25, t (5.6)	58.7, CH	4.72, dd (11.7, 5.9)
13	168.1, C		167.5, C		168.2, C	
15	40.5, C		40.5, C		40.5, C	
15a	28.1, CH_3_	1.55, s	28.1, CH_3_	1.55, s	28.0, CH_3_	1.56, s
15b	28.3, CH_3_	1.54, s	28.2, CH_3_	1.54, s	28.3, CH_3_	1.54, s
16	146.0, CH	6.11, dd (17.3, 10.6)	146.0, CH	6.11, dd (17.3, 10.6)	146.2, CH	6.11, dd (17.2,10.8)
17	112.7,CH_2_	5.11, m	112.7, CH_2_	5.11, m	112.5, CH_2_	5.11, m
18	32.6, CH_2_	1.95, m	31.0, CH_2_	2.21, m	39.0, CH_2_	2.11, m
		2.03, m				2.34, dd (13.0, 5.9)
19	28.4, CH_2_	1.67, m	30.2, CH_2_	2.48, m	68.6, CH	4.51, m
		1.74, m				
20	62.5, CH_2_	3.63, t (6.3)	176.3, C		55.6, CH_2_	3.53, d (13.2)
						3.92, dd (13.2, 4.9)

^a^ Measured in Methanol-d_4_.

**Table 2:** ^1^H (600 MHz) and ^13^C NMR (150 MHz) data for compounds **4**–**6**.

Position	4 ^a^		5 ^b^		6 ^b^	
	δ_C_	δ_H_ (J/Hz)	δ_C_	δ_H_ (J/Hz)	δ_C_	δ_H_ (J/Hz)
1		10.5, s				
2	140.7, C		143.6, C		77.2, CH	5.71, s
3	111.1, C		106.5, C		151.4, C	
3a	127.3, C		128.7, C			
4	110.3, CH	7.30, d (8.0)	112.4, CH	7.40, d (8.1)		
5	118.0, CH	6.86, m	121.0, CH	7.03, m	148.4, C	
6	120.2, CH	6.97, m	122.3, CH	7.09, m	128.2, CH	7.73, d (8.1)
7	121.6, CH	7.70, d (8.0)	121.2, CH	7.74, d (8.0)	136.0, CH	7.84, ddd
7a	135.0, C		136.6, C			(8.0, 7.0, 1.4)
8	68.9, CH	5.30, dd (3.5, 6.2)	77.8, CH	5.60, d (2.4)	128.7, CH	7.56, ddd (8.0, 7.0, 0.8)
9	62.7, CH	4.0, dd (4.7, 6.2)	63.4, CH	4.39, s	127.6, CH	8.14, dd (1.1, 8.0)
10	164.4, C		165.8, C		121.6, C	
11					161.8, C	
12	58.4, CH	3.86, dd (6.9, 9.6)	60.1, CH	4.16, m		
13	169.5, C		170.6, C		58.9, CH	5.37, dd (7.1, 8.4)
14		8.16, d (4.5)			171.8, C	
15	38.7, C		40.1, C		40.4, CH_2_	3.37, dd (7.1, 13.5)
						3.44, dd (8.4, 13.5)
15a	28.1, CH_3_	1.46, s	28.2, CH_3_	1.60, s		
15b	28.2, CH_3_	1.44, s	28.5, CH_3_	1.57, s		
16	146.3, CH	6.16, dd (17.5, 10.5)	146.9, CH	6.16, dd (17.5, 10.5)	128.4, C	
17	110.7, CH_2_	5.09, m	112.6, CH_2_	5.18, ddd (17.5,10.5,0.8)	131.7, CH	7.06, d (8.4)
18	28.8, CH_2_	2.09, m	30.0, CH_2_	1.95, m	116.1, CH	6.64, d (8.4)
		1.73, m		2.34, dd (7.9, 3.4)		
19	21.9, CH_2_	1.83, m	22.9, CH_2_	1.95, m	157.6, C	
				2.06, m		
20	45.3, CH_2_	3.26, m	46.1, CH_2_	3.47, m	116.1, CH	6.64, d (8.4)
		3.40, m		3.76, m		
21					131.7, CH	7.06, d (8.4)
8-OH		5.33, d (3.5)				
8-OCH_3_			56.9, CH_3_	3.17, s		

^a^ Measured in DMSO-d_6_. ^b^ Measured in Methanol-d_4_.

**Table 3:** Inhibitory potency of DPKs to α-glucosidase and PTP1B.

Compd.	α-glucosidase (IC_50_/μM)	PTP1B (Inhibition Ratio/%) ^a^	Compd.	α-glucosidase (IC_50_/μM)	PTP1B (Inhibition Ratio/%) ^a^
**1**	18.2	29	**10**	40.7	<10
**2**	130.7	<10	**11**	37.5	<10
**3**	83.9	<10	**12**	1086.6	<10
**4**	144.2	<10	**13**	480.5	<10
**5**	1093.5	<10	**14**	353.2	<10
**6**	267.3	<10	**15**	67.8	<10
**7**	198.2	<10	**16**	362.6	<10
**8**	364.3	<10	Acarbose ^b^	408.0	-
**9**	7.6	<10	Oleanolic acid ^c^	-	99.1

^a^ The inhibition ratio screening concentration was 100 μg/mL for the PTP1B test; ^b^ Acarbose was used as a positive control for the α-glucosidase inhibitory test; ^c^ Oleanolic acid was used as a positive control for PTP1B inhibitory test.

## Data Availability

Not applicable.

## Supplementary Materials

The HRESIMS and NMR spectrum (Figures S1–S61) are available online at https://www.mdpi.com/article/10.3390/md19070402/s1.
