# Supplementary material for: Isolation, Structural Characterization and Antidiabetic Activity of New Diketopiperazine Alkaloids from Mangrove Endophytic Fungus Aspergillus sp. 16-5c
Source: Mar Drugs. 2021 Jul 20;19(7):402. doi: 10.3390/md19070402 (PMC8304462; doi:10.3390/md19070402)
Supplement: Supplementary file 1 [file marinedrugs-19-00402-s001.zip › marinedrugs-1308118-supplementary.pdf]

# Supporting Information

## Isolation, structural characterization and antidiabetic activity of new diketopiperazine alkaloids from mangrove endophytic fungus *Aspergillus* sp. 16-5c

Geting Ye [a], Cuiying Huang [a], Jialin Li [a], Tao Chen [a], Jing Tang [a], Wenbin Liu [a], Yuhua Long [a,*]

[a] *School of Chemistry, Guangzhou Key Laboratory of Analytical Chemistry for Biomedicine, South China Normal University, Guangzhou 510006, China.*

* Correspondence: longyh@scnu.edu.cn (Y.L.)

## Table of Contents

Figure S1 HRESIMS of compound **1**
Figure S2 $^{1}$H NMR spectrum (600 MHz, methanol-$d_4$) of compound **1**
Figure S3 $^{13}$C NMR spectrum (600 MHz, methanol-$d_4$) of compound **1**
Figure S4 HMQC spectrum (600 MHz, methanol-$d_4$) of compound **1**
Figure S5 HMBC spectrum (600 MHz, methanol-$d_4$) of compound **1**
Figure S6 $^{1}$H-$^{1}$H COSY spectrum (600 MHz, methanol-$d_4$) of compound **1**
Figure S7 NOESY spectrum (600 MHz, DMSO-$d_6$) of compound **1**
Figure S8 HRESIMS of compound **2**
Figure S9 $^{1}$H NMR spectrum (600 MHz, methanol-$d_4$) of compound **2**
Figure S10 $^{13}$C NMR spectrum (600 MHz, methanol-$d_4$) of compound **2**
Figure S11 HMQC spectrum (600 MHz, methanol-$d_4$) of compound **2**
Figure S12 HMBC spectrum (600 MHz, methanol-$d_4$) of compound **2**
Figure S13 $^{1}$H-$^{1}$H COSY spectrum (600 MHz, DMSO -$d_4$) of compound **2**
Figure S14 NOESY spectrum (600 MHz, DMSO-$d_6$) of compound **2**
Figure S15 HRESIMS of compound **3**
Figure S16 $^{1}$H NMR spectrum (600 MHz, methanol-$d_4$) of compound **3**
Figure S17 $^{13}$C NMR spectrum (600 MHz, methanol-$d_4$) of compound **3**
Figure S18 HMQC spectrum (600 MHz, methanol-$d_4$) of compound **3**
Figure S19 HMBC spectrum (600 MHz, methanol-$d_4$) of compound **3**
Figure S20 $^{1}$H-$^{1}$H COSY spectrum (600 MHz, methanol-$d_4$) of compound **3**
Figure S21 NOESY spectrum (600 MHz, DMSO-$d_6$) of compound **3**
Figure S22 HRESIMS of compound **4**
Figure S23 $^{1}$H NMR spectrum (600 MHz, DMSO-$d_6$) of compound **4**
Figure S24 $^{13}$C NMR spectrum (600 MHz, DMSO-$d_6$) of compound **4**
Figure S25 HMQC spectrum (600 MHz, DMSO-$d_6$) of compound **4**
Figure S26 HMBC spectrum (600 MHz, DMSO-$d_6$) of compound **4**
Figure S27 $^{1}$H-$^{1}$H COSY spectrum (600 MHz, DMSO-$d_6$) of compound **4**
Figure S28 NOESY spectrum (600 MHz, DMSO-$d_6$) of compound **4**
Figure S29 HRESIMS of compound **5**
Figure S30$^{1}$H NMR spectrum (600 MHz, methanol-$d_4$) of compound **5**
Figure S31 $^{13}$C NMR spectrum (600 MHz, methanol-$d_4$) of compound **5**
Figure S32 HMQC spectrum (600 MHz, methanol-*d*4) of compound **5**
Figure S33 HMBC spectrum (600 MHz, methanol-$d_4$) of compound **5**
Figure S34 $^{1}$H-$^{1}$H COSY spectrum (600 MHz, methanol-$d_4$) of compound **5**
Figure S35 HRESIMS of compound **6**
Figure S36 $^{1}$H NMR spectrum (600 MHz, methanol-$d_4$) of compound **6**
Figure S37 $^{13}$C NMR spectrum (600 MHz, methanol-$d_4$) of compound **6**
Figure S38 HMQC spectrum (600 MHz, methanol-$d_4$) of compound **6**
Figure S39 HMBC spectrum (600 MHz, methanol-$d_4$) of compound **6**
Figure S40 $^{1}$H-$^{1}$H COSY spectrum (600 MHz, methanol-$d_4$) of compound **6**
Figure S41 NOESY spectrum (600 MHz, DMSO-$d_6$) of compound **6**

Figure S42 $^{1}$H NMR spectrum (600 MHz, methanol-$d_4$) of compound **7**
Figure S43 $^{13}$C NMR spectrum (600 MHz, methanol-$d_4$) of compound **7**
Figure S44 $^{1}$H NMR spectrum (600 MHz, methanol-$d_4$) of compound **8**
Figure S45 $^{13}$C NMR spectrum (600 MHz, methanol-$d_4$) of compound **8**
Figure S46 $^{1}$H NMR spectrum (600 MHz, chloroform -*d*) of compound **9**
Figure S47 $^{13}$C NMR spectrum (600 MHz, chloroform -*d*) of compound **9**
Figure S48$^{1}$H NMR spectrum (600 MHz, methanol-$d_4$) of compound **10**
Figure S49 $^{13}$C NMR spectrum (600 MHz, methanol-$d_4$) of compound **10**
Figure S50 $^{1}$H NMR spectrum (600 MHz, chloroform -*d*) of compound **11**
Figure S51 $^{13}$C NMR spectrum (600 MHz, chloroform -*d*) of compound **11**
Figure S52 $^{1}$H NMR spectrum (600 MHz, chloroform -*d*) of compound **12**
Figure S53 $^{13}$C NMR spectrum (600 MHz, chloroform -*d*) of compound **12**
Figure S54 $^{1}$H NMR spectrum (600 MHz, methanol-$d_4$) of compound **13**
Figure S55 $^{13}$C NMR spectrum (600 MHz, methanol-$d_4$) of compound **13**
Figure S56 $^{1}$H NMR spectrum (600 MHz, DMSO-$d_6$) of compound **14**
Figure S57 $^{13}$C NMR spectrum (600 MHz, DMSO-$d_6$) of compound **14**
Figure S58 $^{1}$H NMR spectrum (600 MHz, DMSO-$d_6$) of compound **15**
Figure S59 $^{13}$C NMR spectrum (600 MHz, DMSO-$d_6$) of compound **15**
Figure S60 $^{1}$H NMR spectrum (600 MHz, acetone-$d_6$) of compound **16**
Figure S61 $^{13}$C NMR spectrum (600 MHz, acetone-$d_6$) of compound **16**

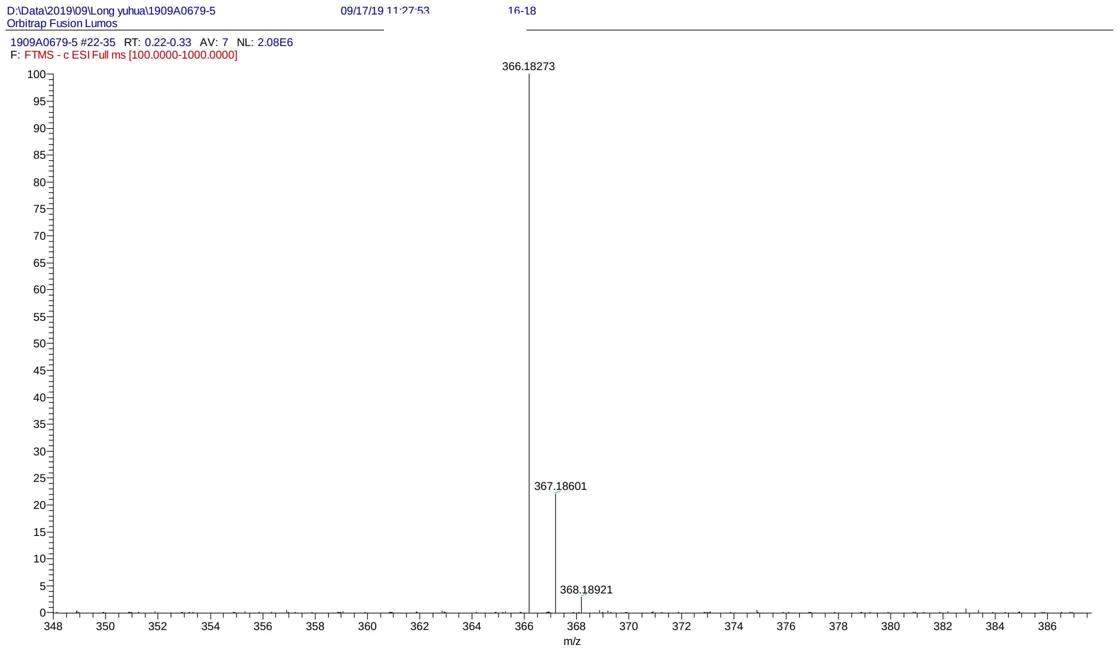


SPECTRUM - simulation :

| m/z | Theo. Mass | Delta (ppm) | RDB equiv. | Composition |
|---|---|---|---|---|
| 366.18273 | 366.18231 | 1.13 | 11.5 | $C_{21}$ $H_{24}$ $O_3$ $N_3$ |

Figure S1 HRESIMS of compound **1**

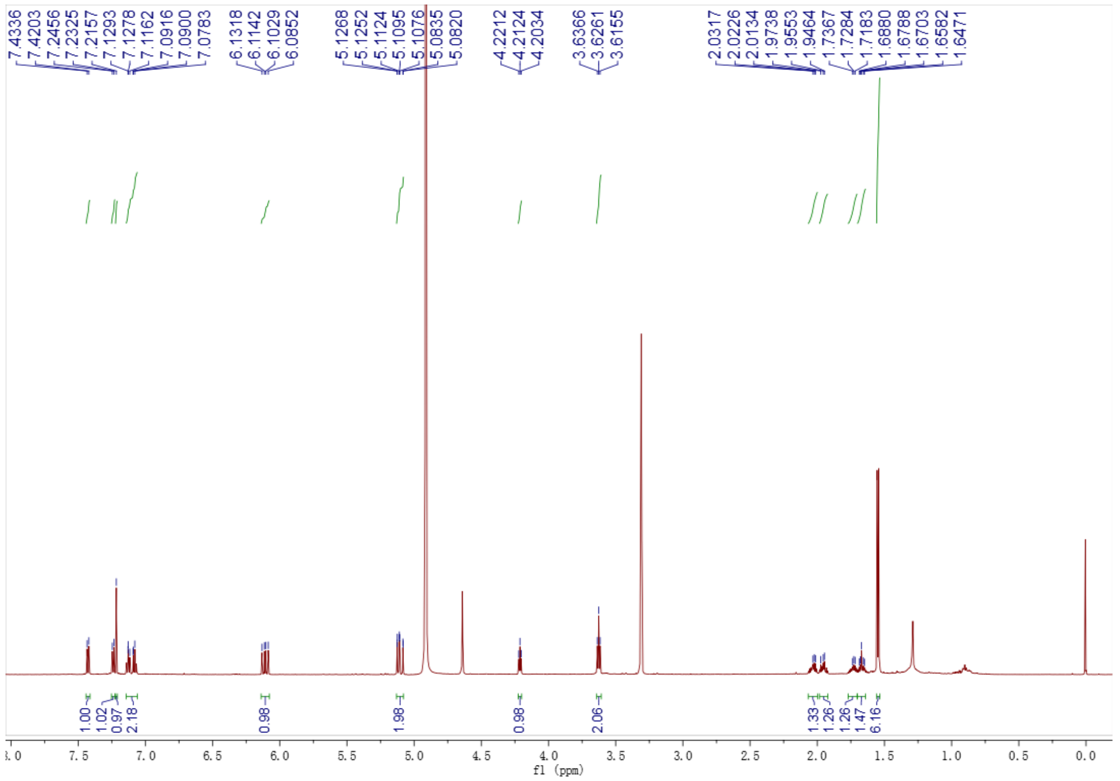


Figure S2 $^{1}$H NMR spectrum (600 MHz, methanol-$d_4$) of compound **1**

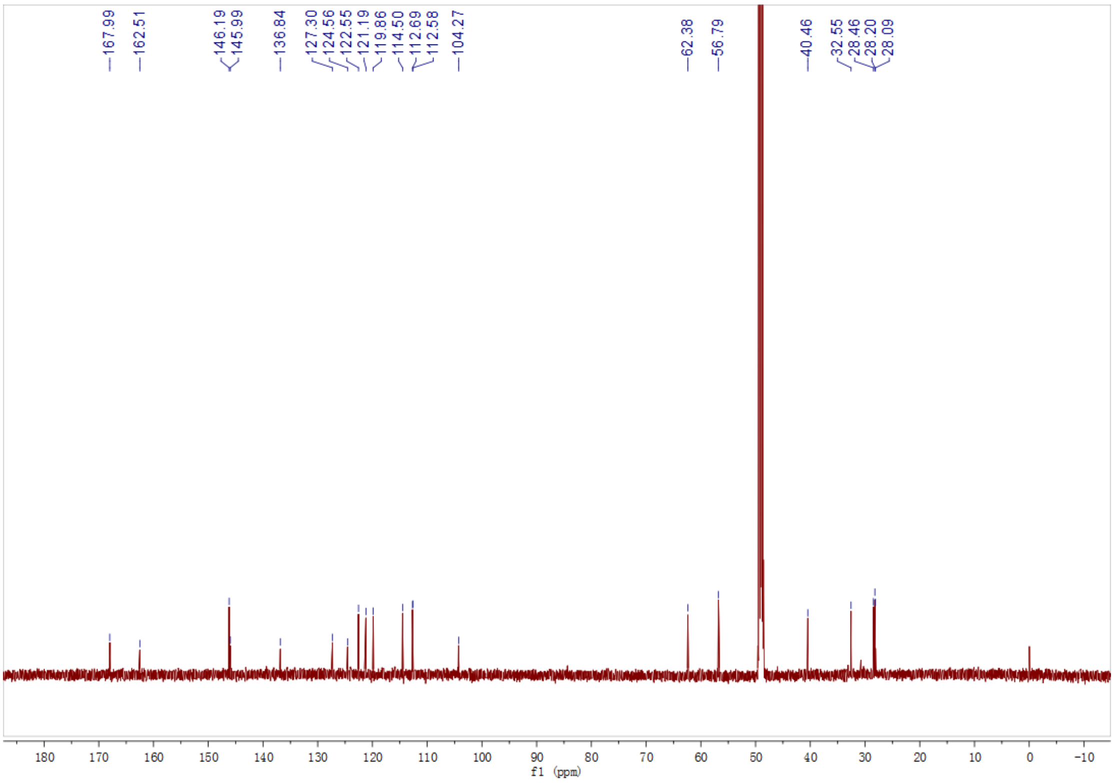


Figure S3 $^{13}$C NMR spectrum (600 MHz, methanol-$d_4$) of compound **1**

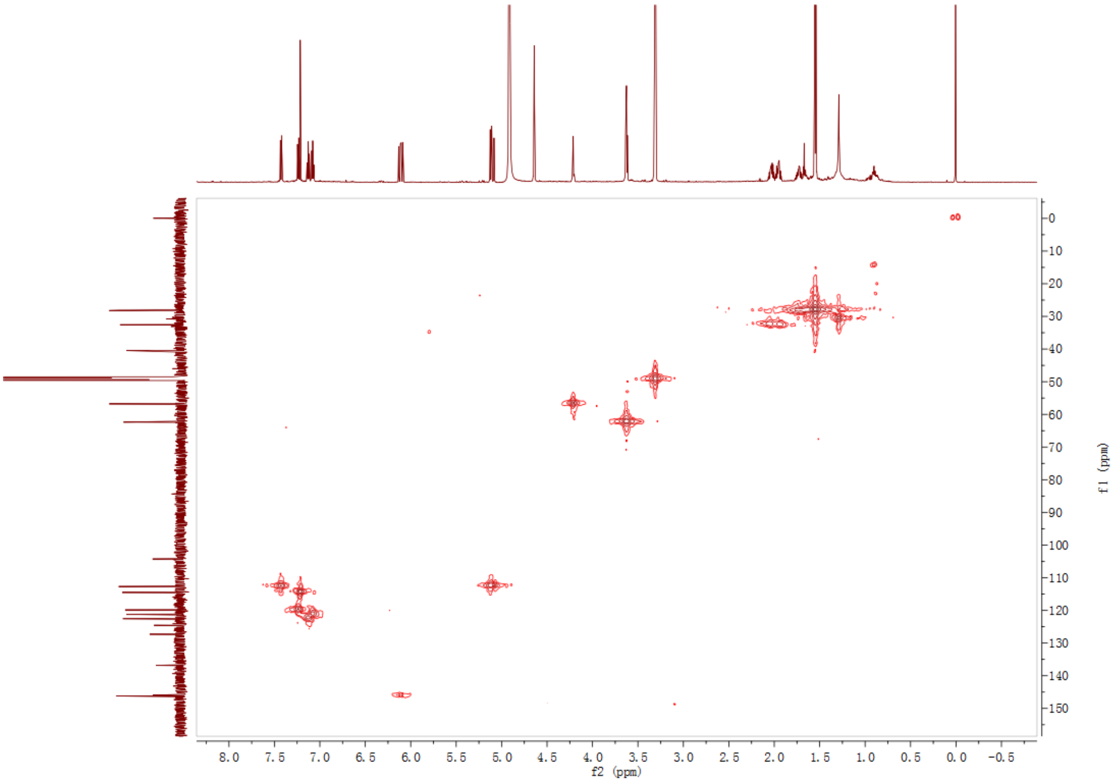


Fig.S4 HMQC spectrum (600 MHz, methanol-$d_4$) of compound **1**

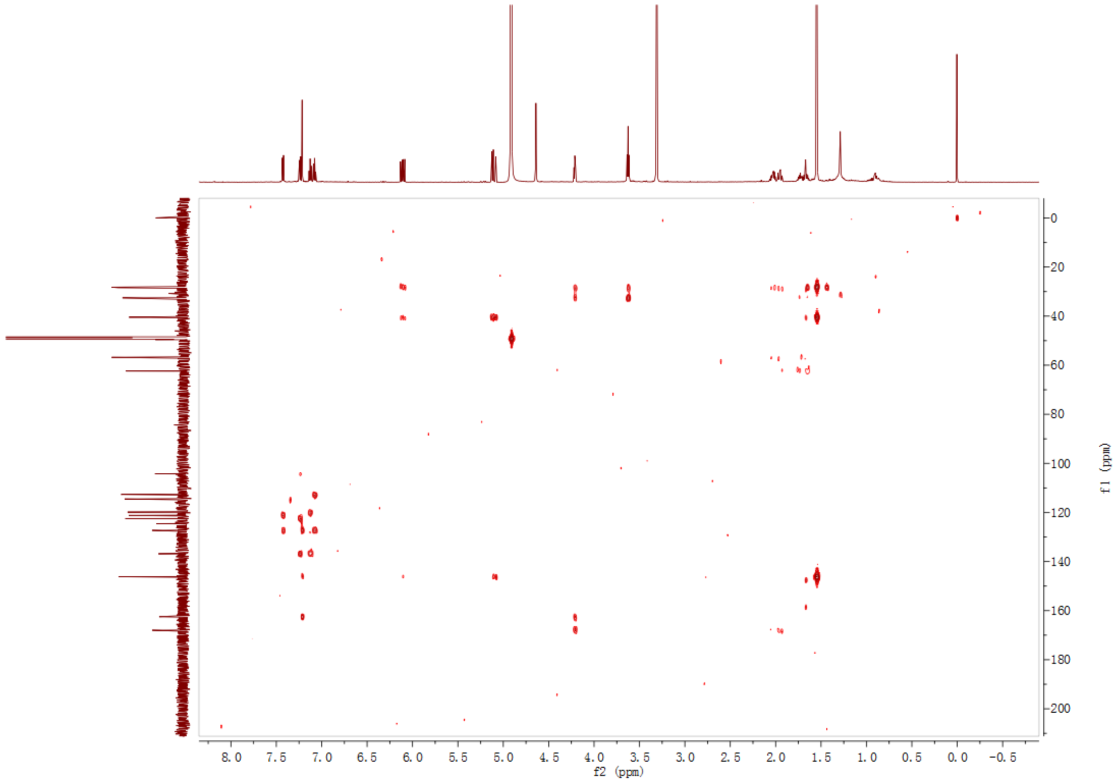


Figure S5 HMBC spectrum (600 MHz, methanol-$d_4$) of compound **1**

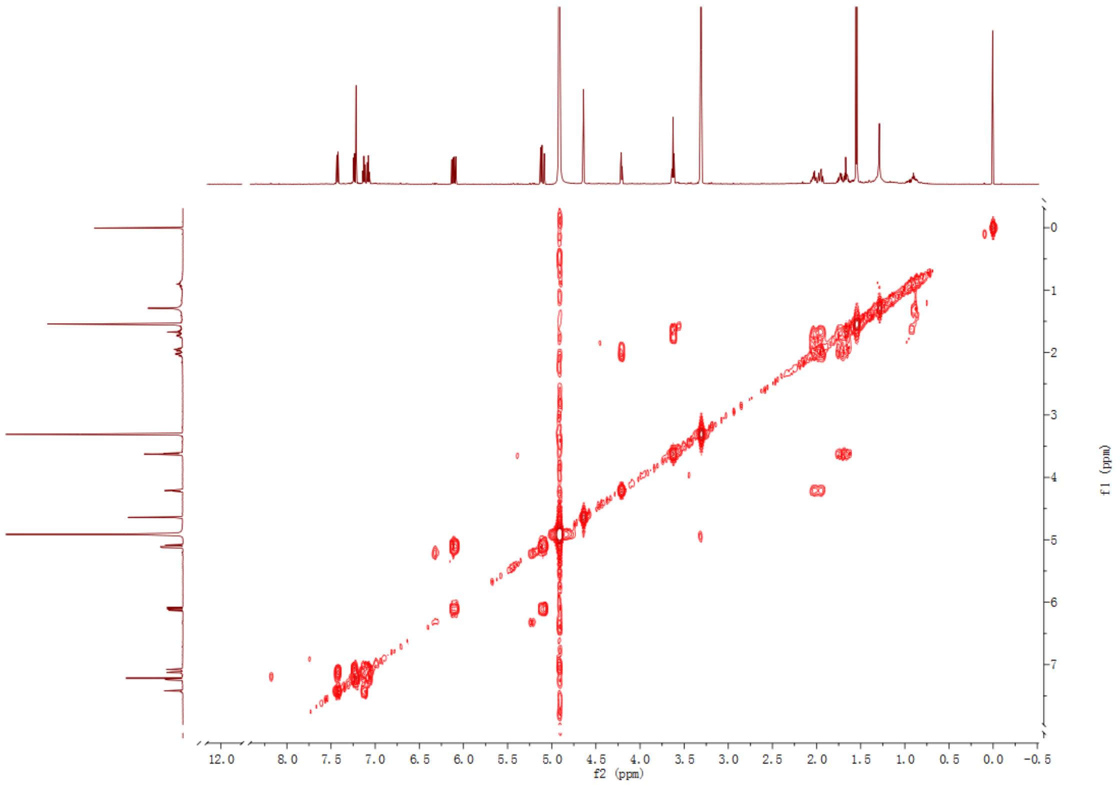


Figure S6 $^{1}H$-$^{1}H$ COSY spectrum (600 MHz, methanol-$d_4$) of compound **1**

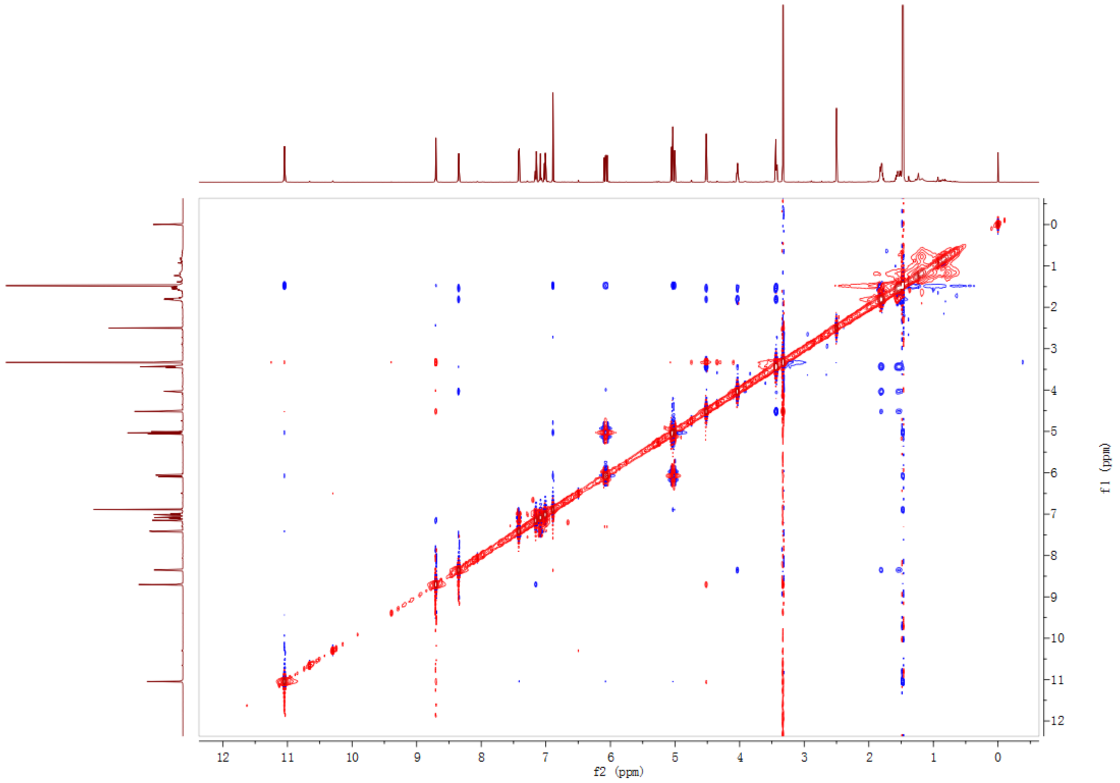


Figure S7 NOESY spectrum (600 MHz, DMSO-$d_6$) of compound **1**

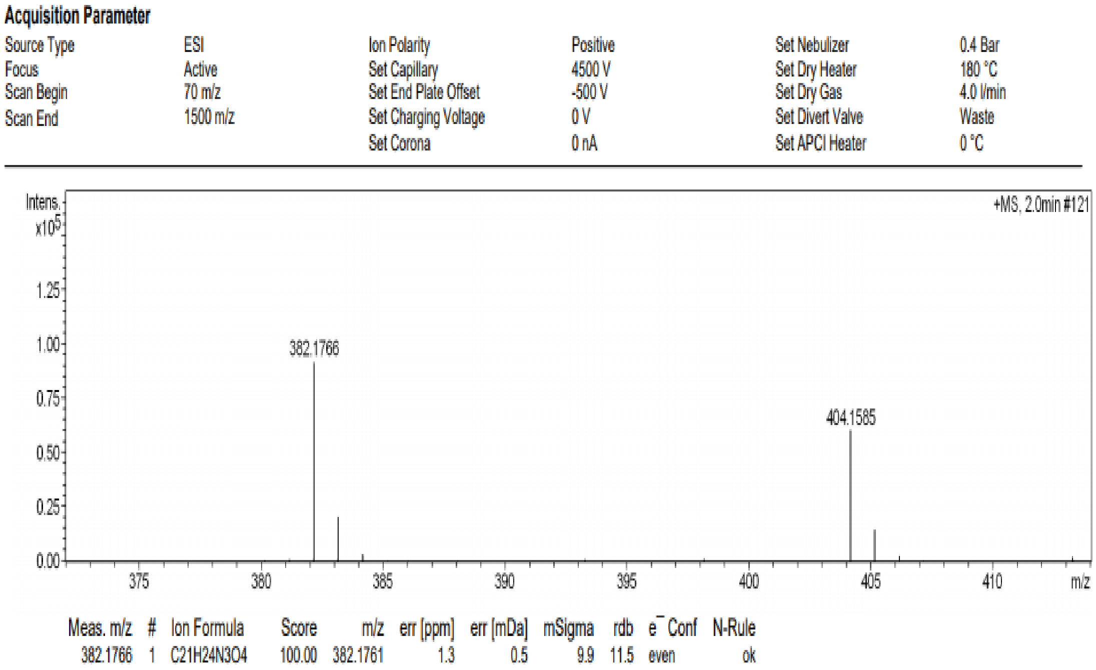


Figure S8 HRESIMS of compound **2**

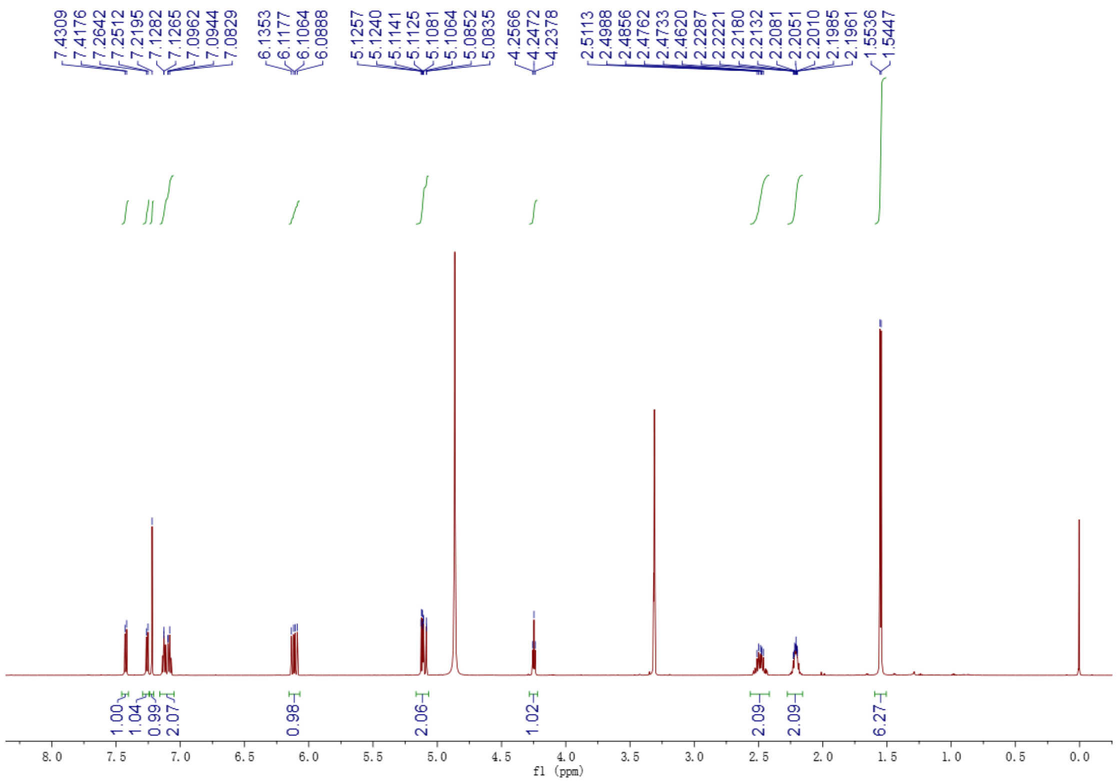


Figure S9 $^{1}$H NMR spectrum (600 MHz, methanol-$d_4$) of compound **2**

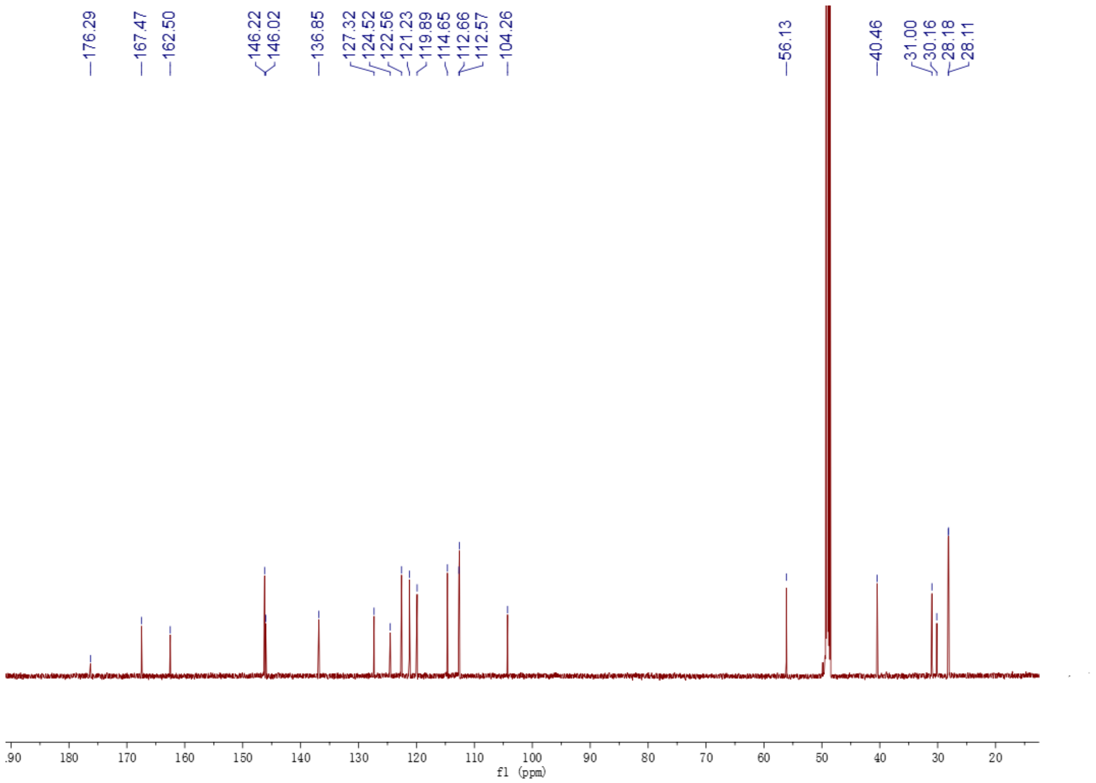


Figure S10 $^{13}$C NMR spectrum (600 MHz, methanol-$d_4$) of compound **2**

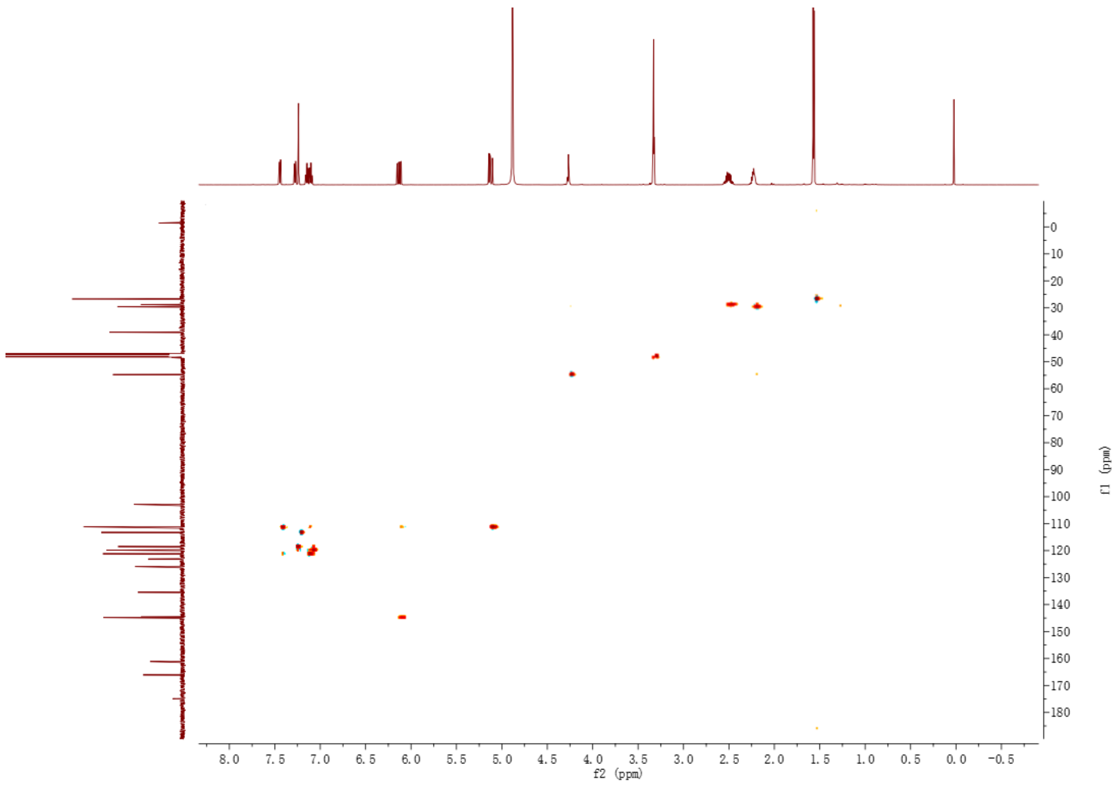


Figure S11 HMQC spectrum (600 MHz, methanol-$d_4$) of compound **2**

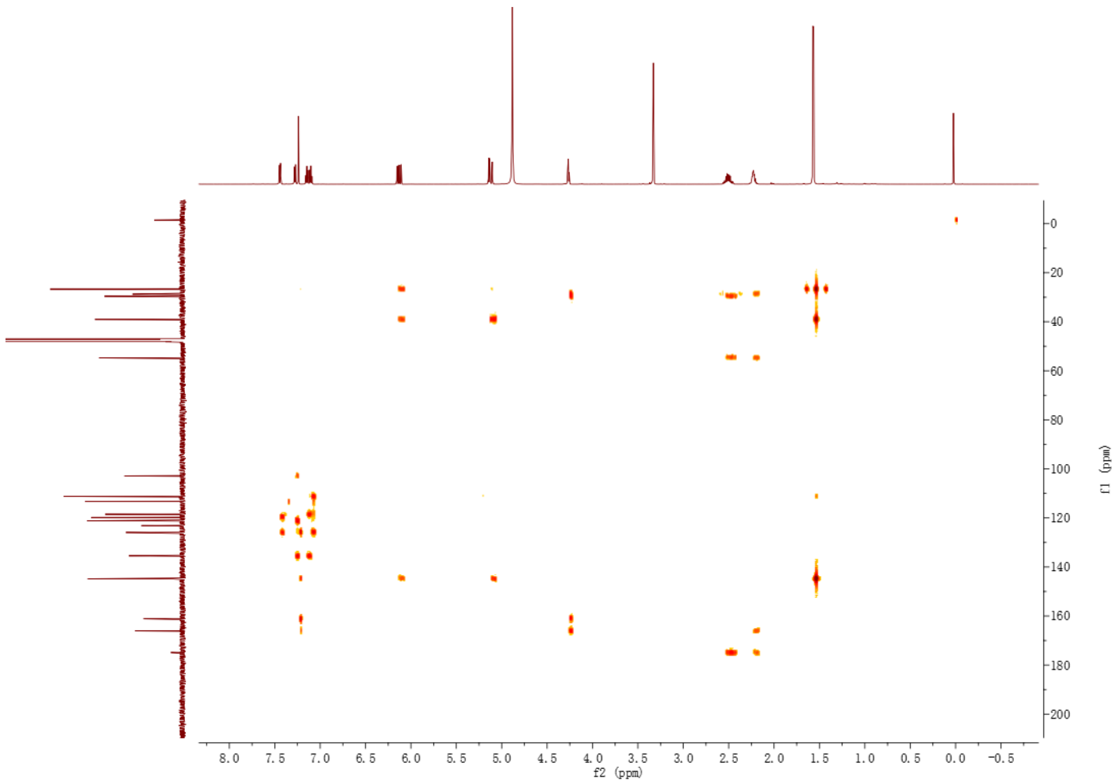


Figure S12 HMBC spectrum (600 MHz, methanol-$d_4$) of compound **2**

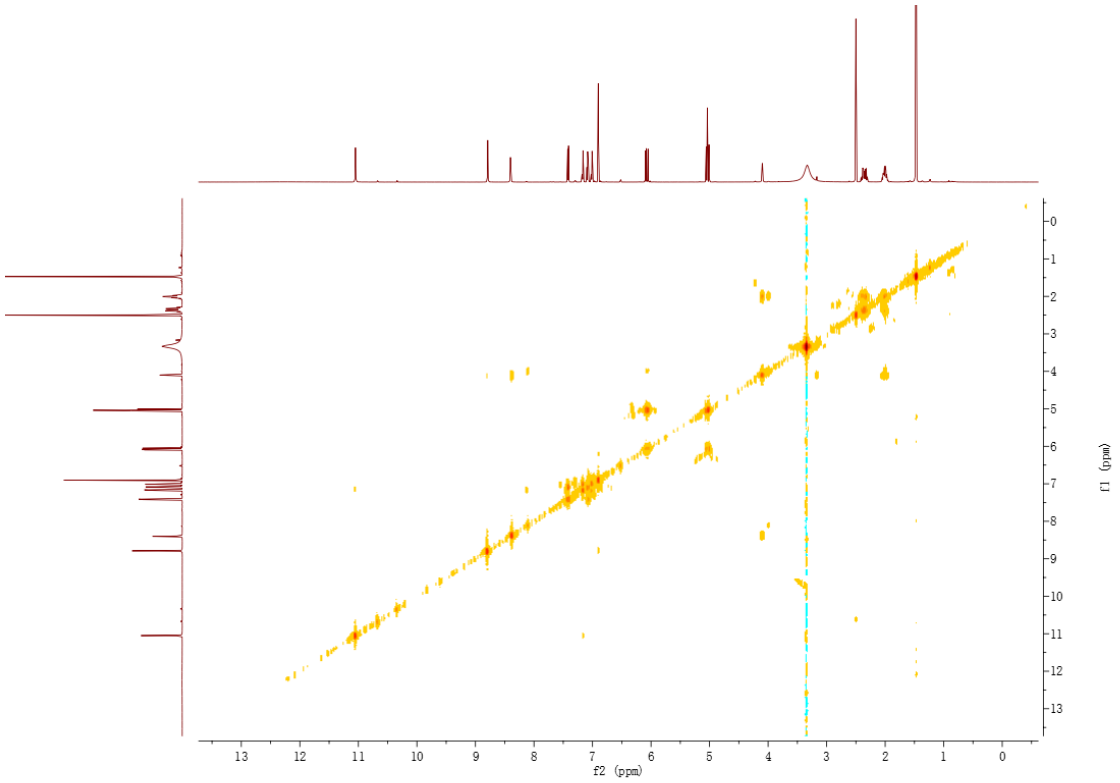


Figure S13 $^{1}$H-$^{1}$H COSY spectrum (600 MHz, DMSO-$d_4$) of compound **2**

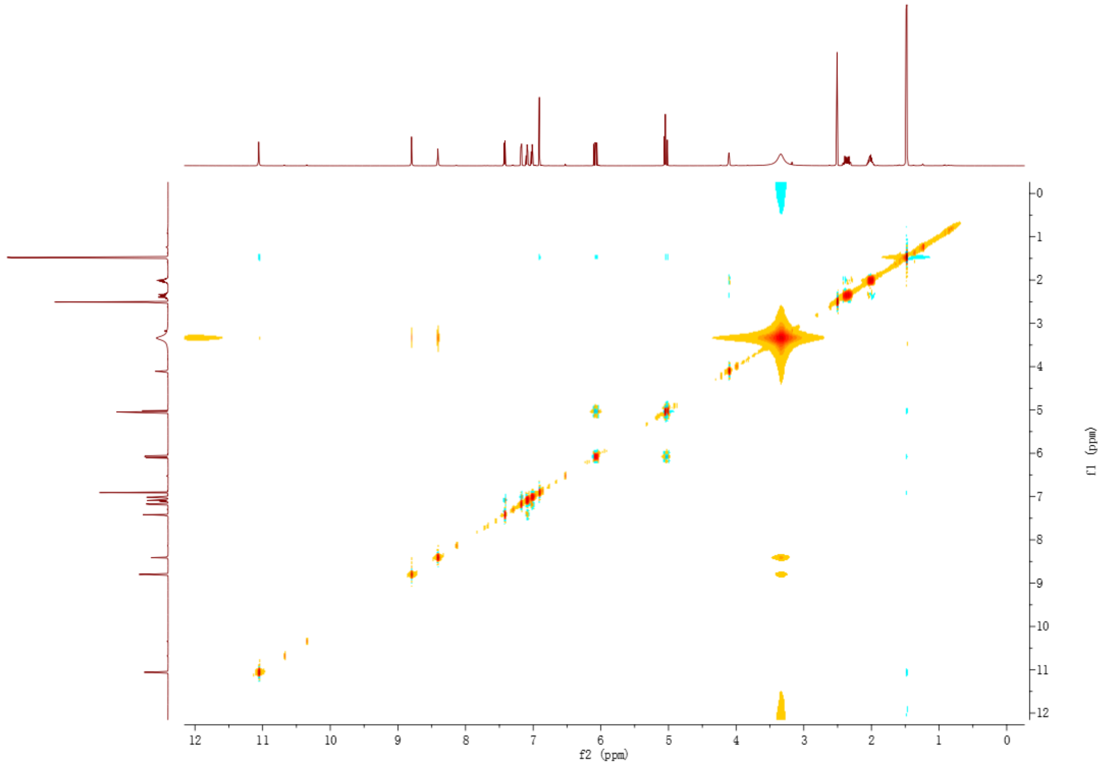


Figure S14 NOESY spectrum (600 MHz, DMSO-$d_6$) of compound **2**

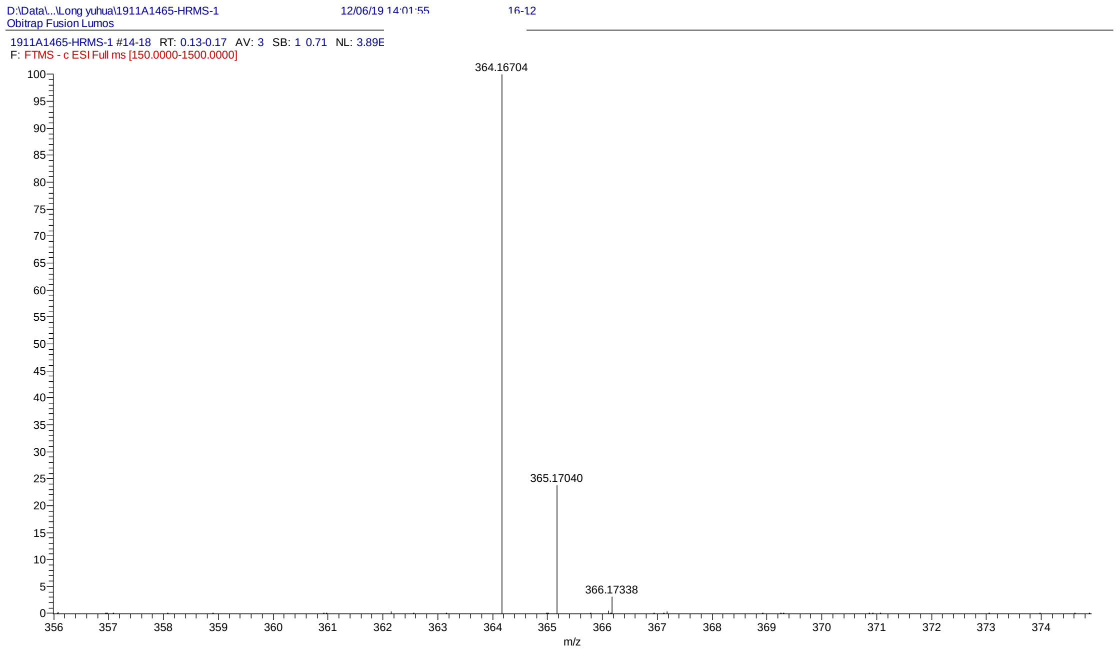


SPECTRUM - simulation :

| m/z | Theo. Mass | Delta (ppm) | RDB equiv. | Composition |
|---|---|---|---|---|
| 364.16704 | 364.16666 | 1.03 | 12.5 | $C_{21} H_{22} O_3 N_3$ |

Figure S15 HRESIMS of compound **3**

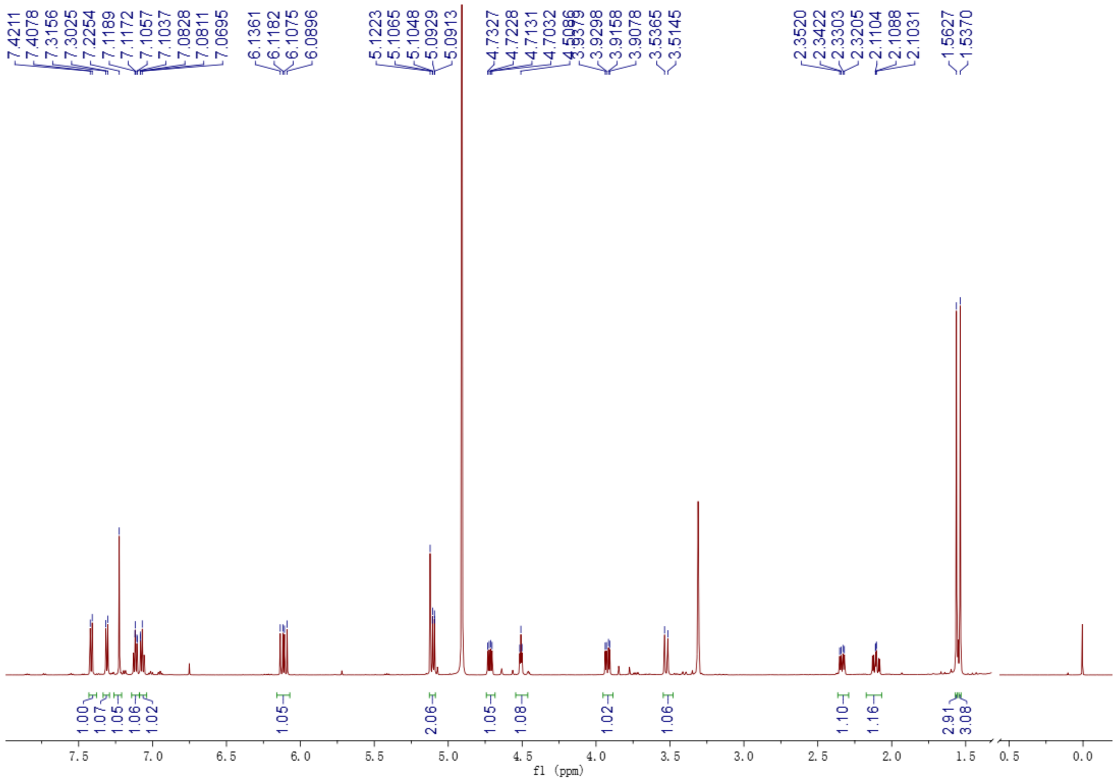


Figure S16 $^{1}$H NMR spectrum (600 MHz, methanol-$d_4$) of compound **3**

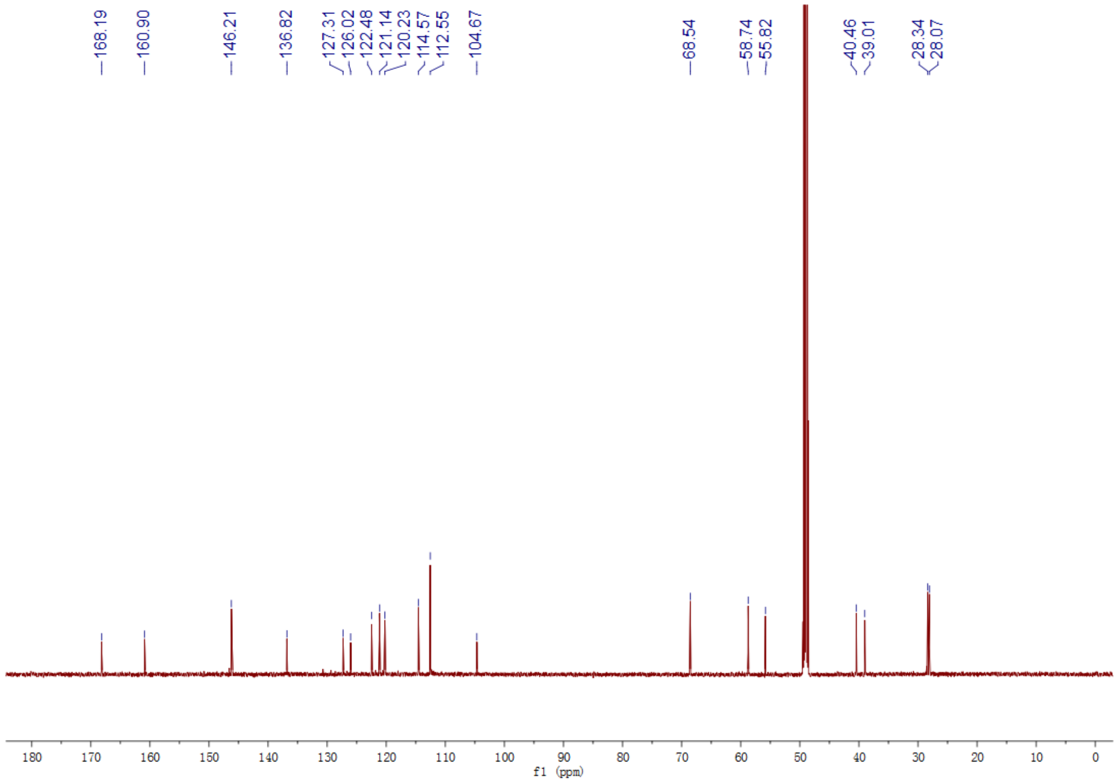


Figure S17 $^{13}$C NMR spectrum (600 MHz, methanol-$d_4$) of compound **3**

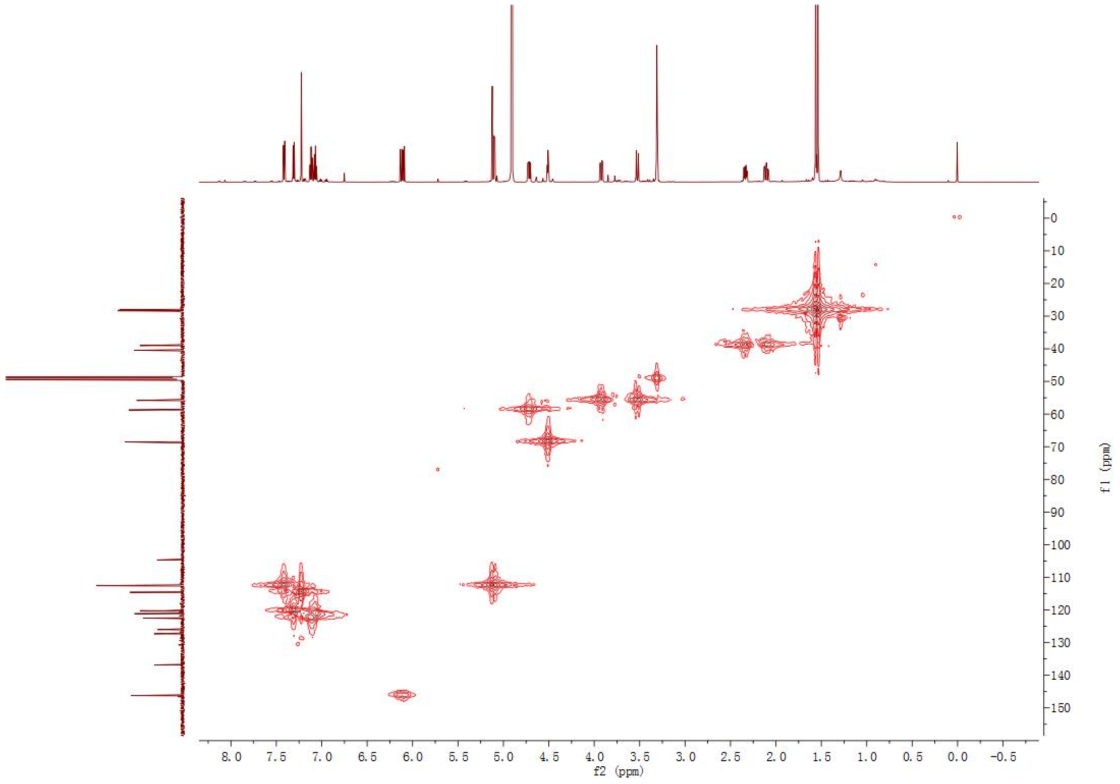


Figure.S18 HMQC spectrum (600 MHz, methanol-$d_4$) of compound **3**

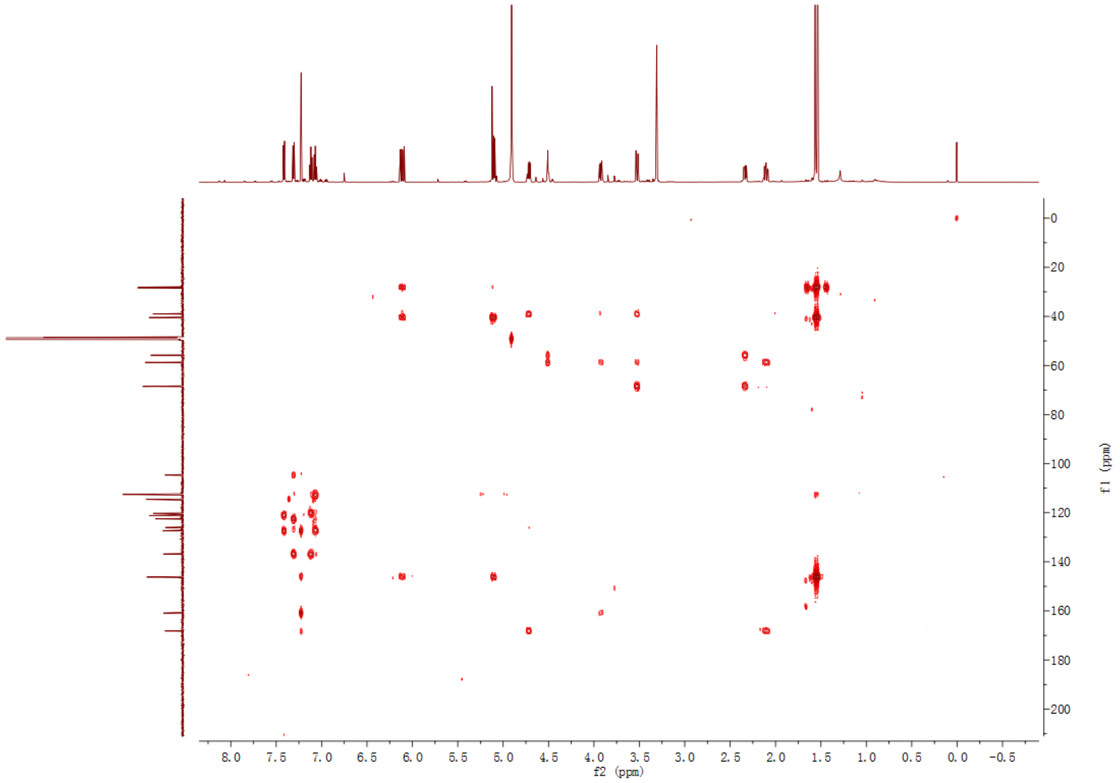


Figure S19 HMBC spectrum (600 MHz, methanol-$d_4$) of compound **3**

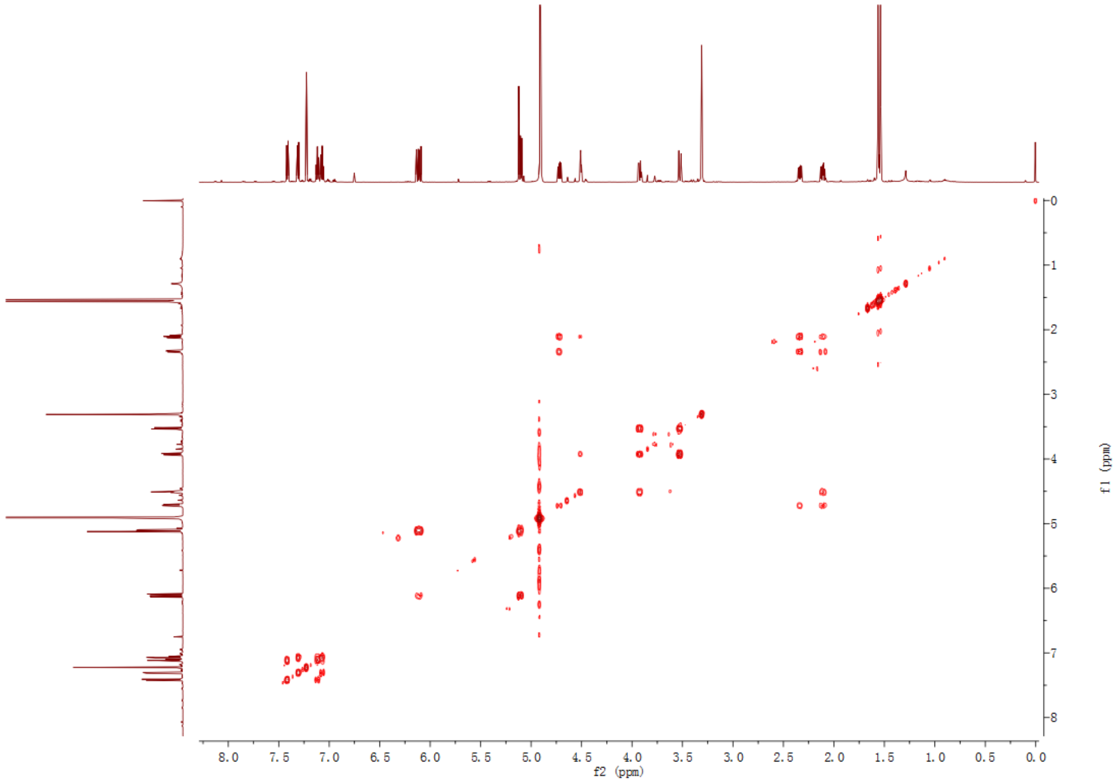


Figure S20 $^1H$-$^1H$ COSY spectrum (600 MHz, methanol-$d_4$) of compound **3**

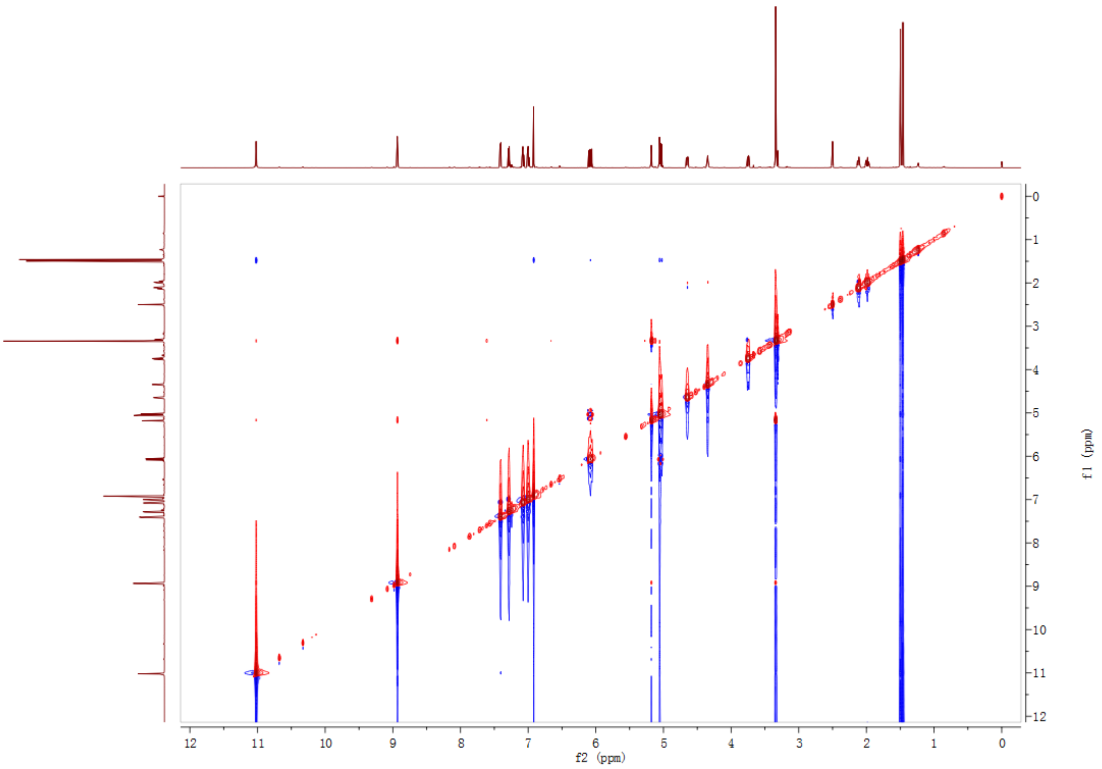


Figure S21 NOESY spectrum (600 MHz, DMSO-$d_6$) of compound **3**

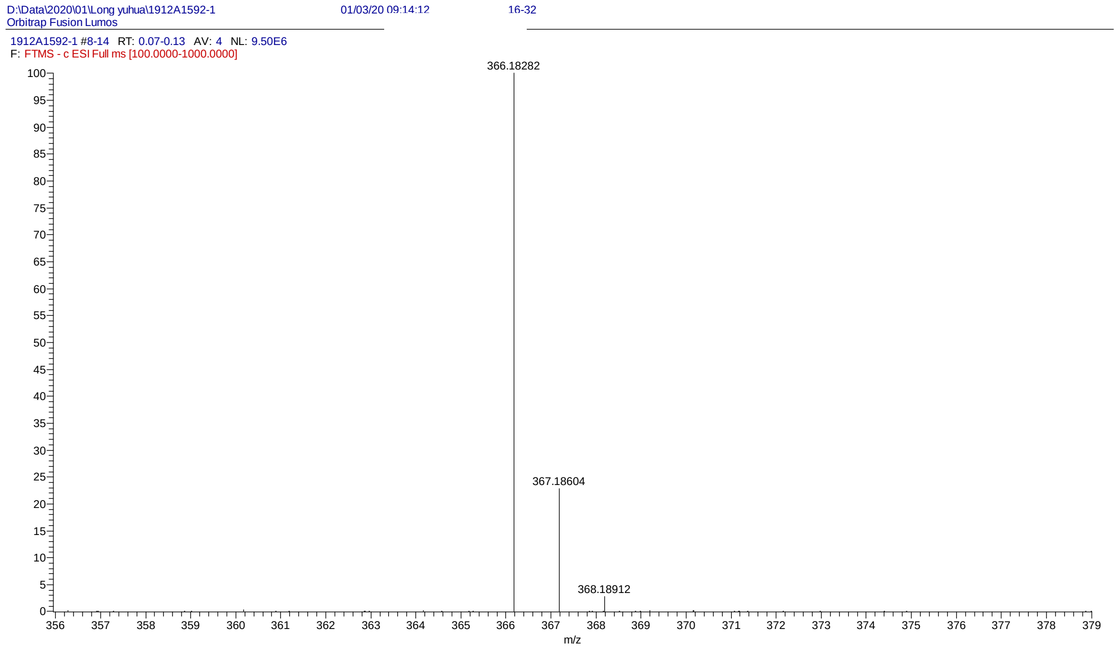


SPECTRUM - simulation :

| m/z | Theo. Mass | Delta (ppm) | RDB equiv. | Composition |
|---|---|---|---|---|
| 366.18282 | 366.18231 | 1.38 | 11.5 | $C_{21} H_{24} O_3 N_3$ |

Figure S22 HRESIMS of compound **4**

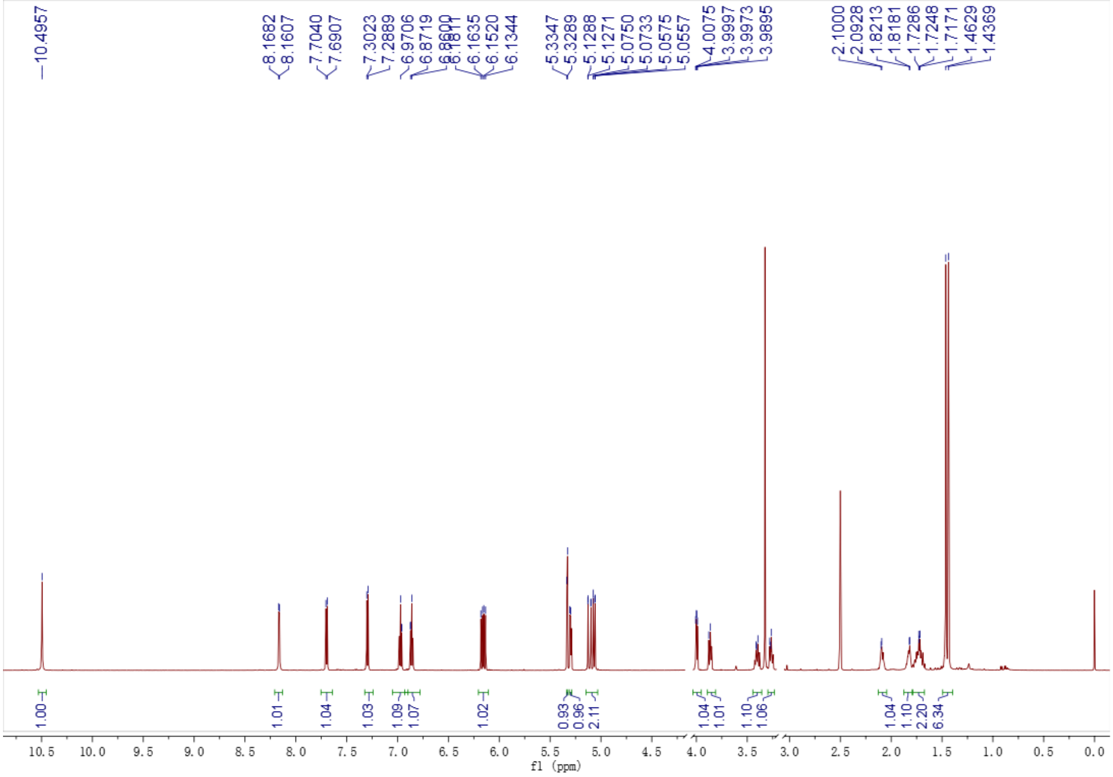


Figure S23 $^{1}$H NMR spectrum (600 MHz, DMSO-$d_6$) of compound **4**

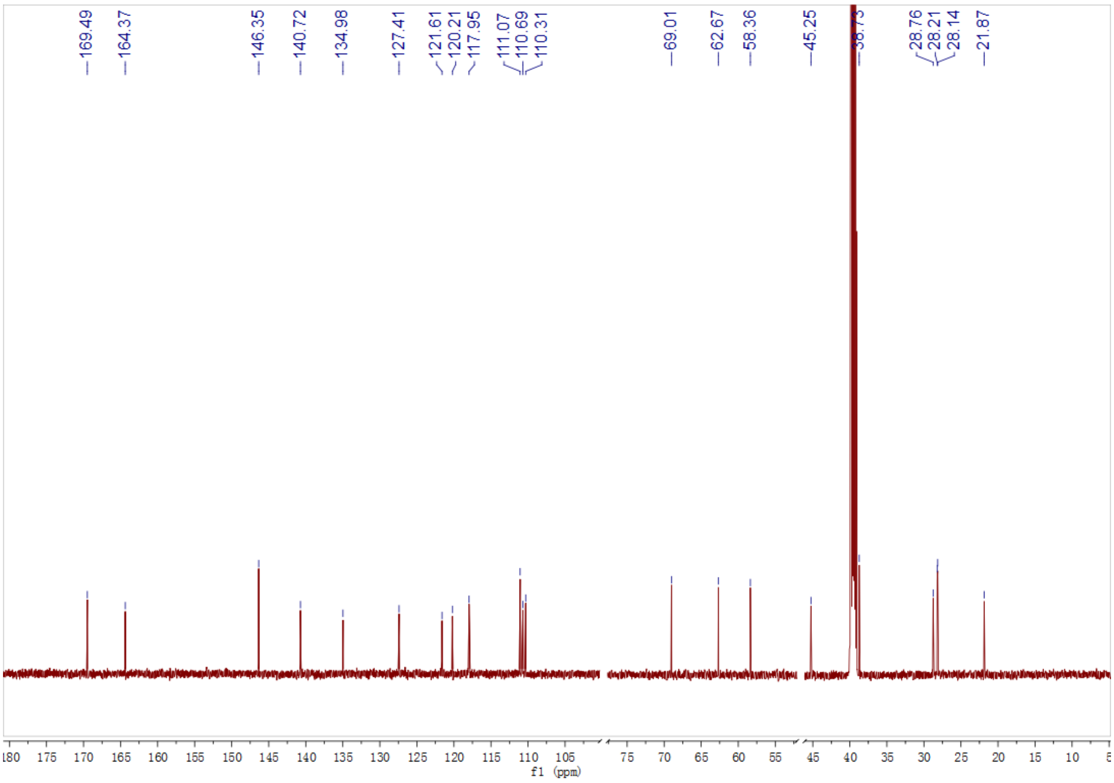


Figure S24 $^{13}$C NMR spectrum (600 MHz, DMSO-$d_6$) of compound **4**

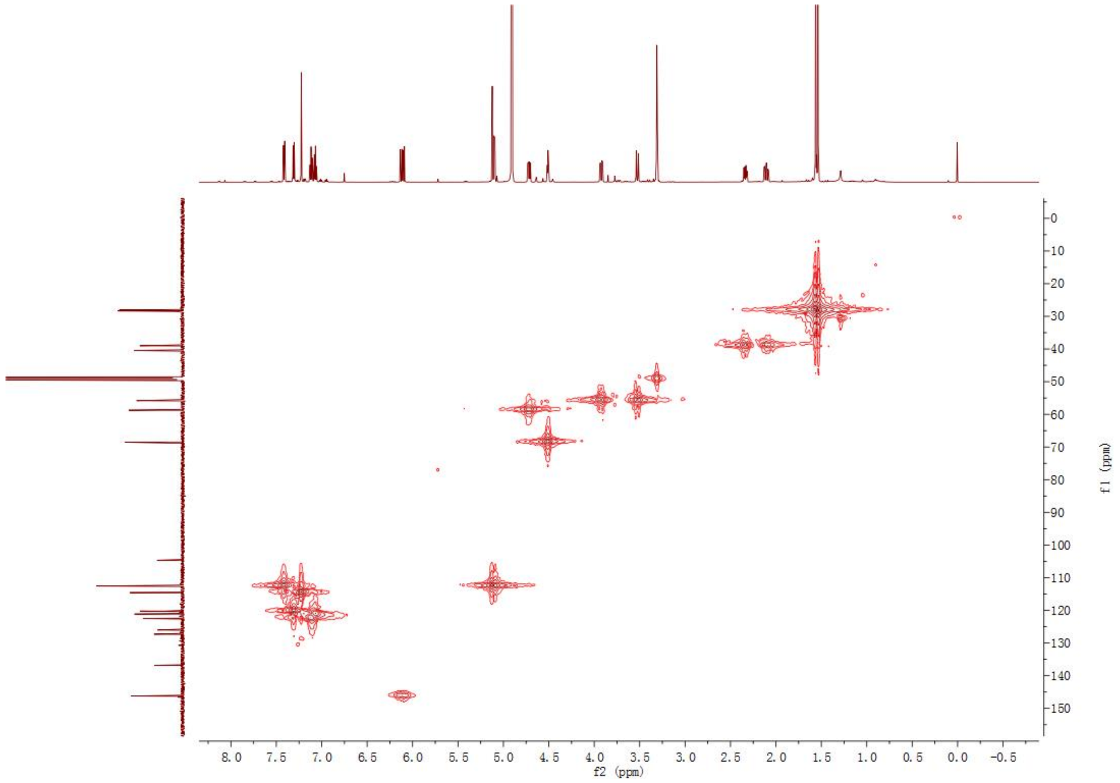


Figure S25 HMQC spectrum (600 MHz, DMSO-$d_6$) of compound **4**

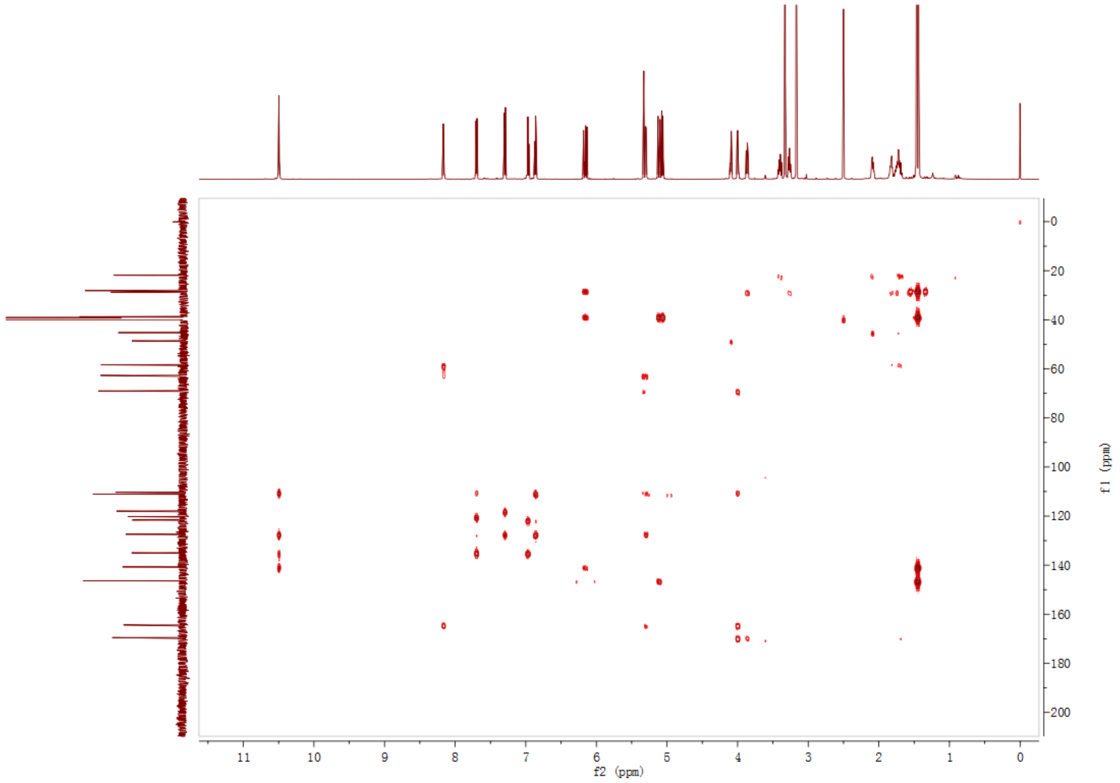


Figure S26 HMBC spectrum (600 MHz, DMSO-$d_6$) of compound **4**

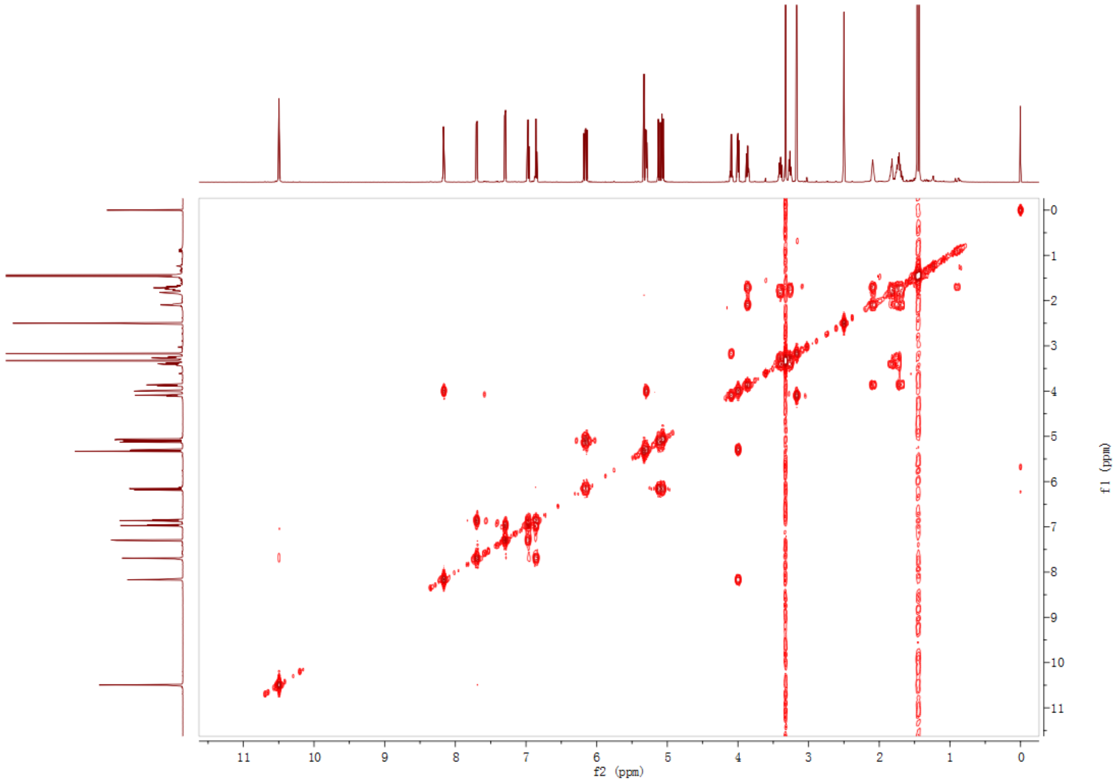


Figure S27 $^{1}H$-$^{1}H$ COSY spectrum (600 MHz, DMSO-*d*$_6$) of compound **4**

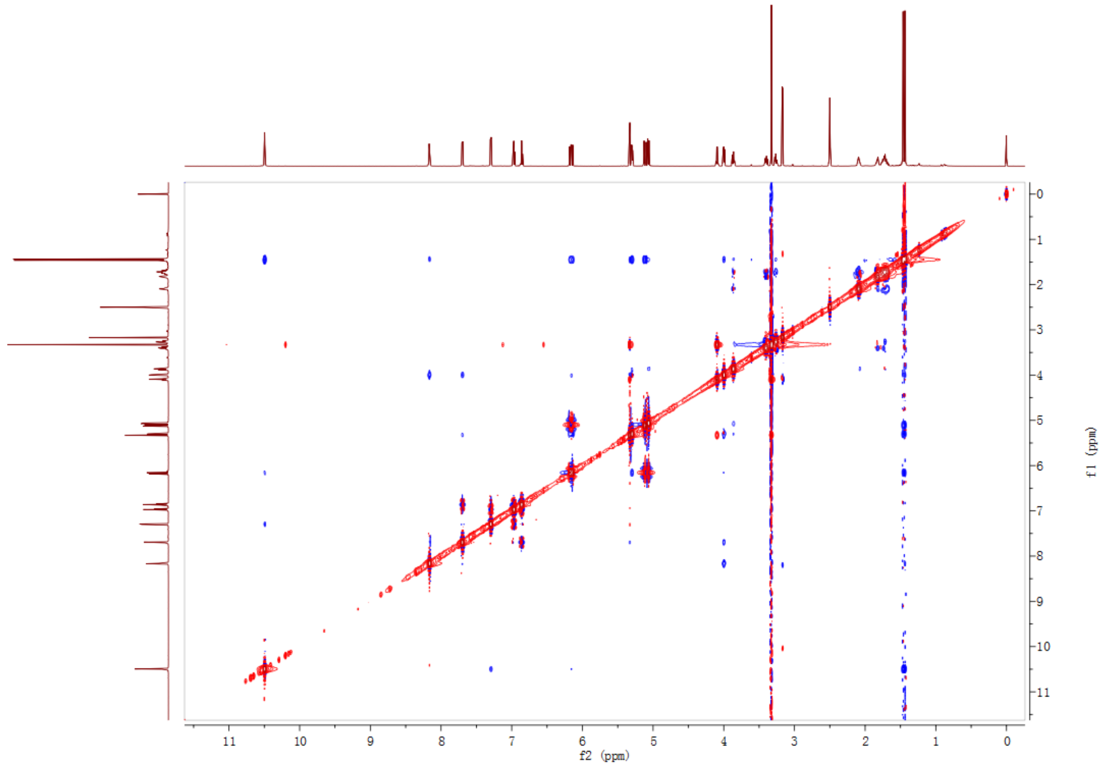


Figure S28 NOESY spectrum (600 MHz, DMSO-*d*$_6$) of compound **4**

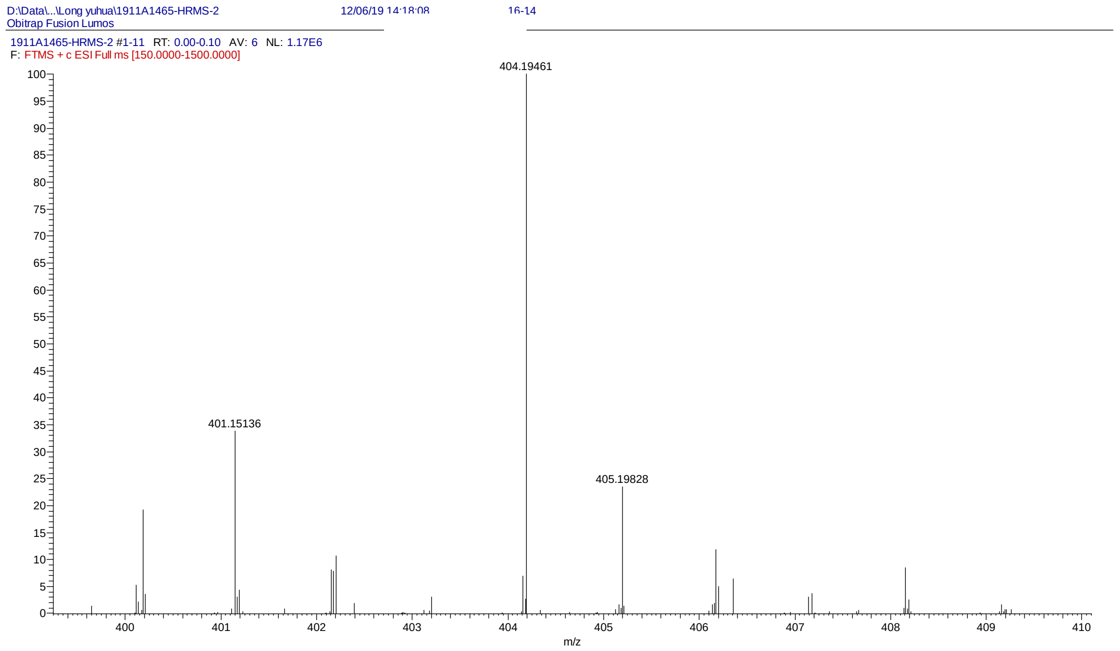


SPECTRUM - simulation :

| m/z | Theo. Mass | Delta (ppm) | RDB equiv. | Composition |
|---|---|---|---|---|
| 404.19461 | 404.19446 | 0.36 | 10.5 | $C_{22}H_{27}O_3N_3Na$ |

Figure S29 HRESIMS of compound **5**

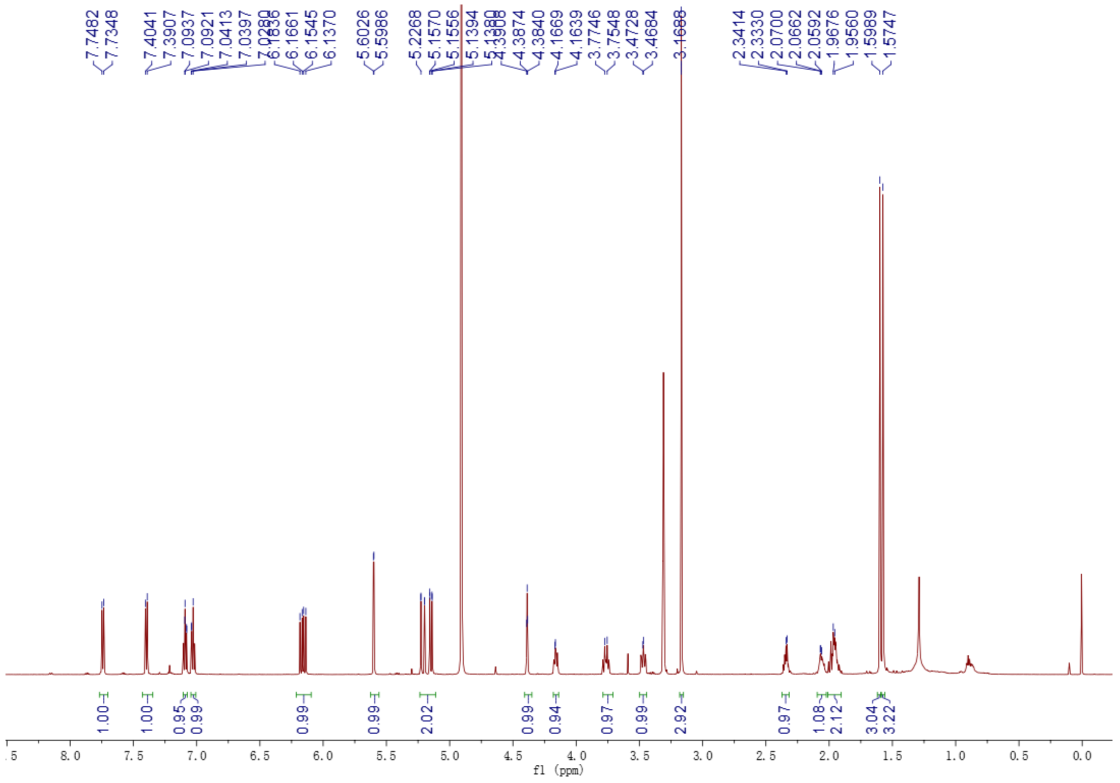


Figure S30 $^1$H NMR spectrum (600 MHz, methanol-$d_4$) of compound **5**

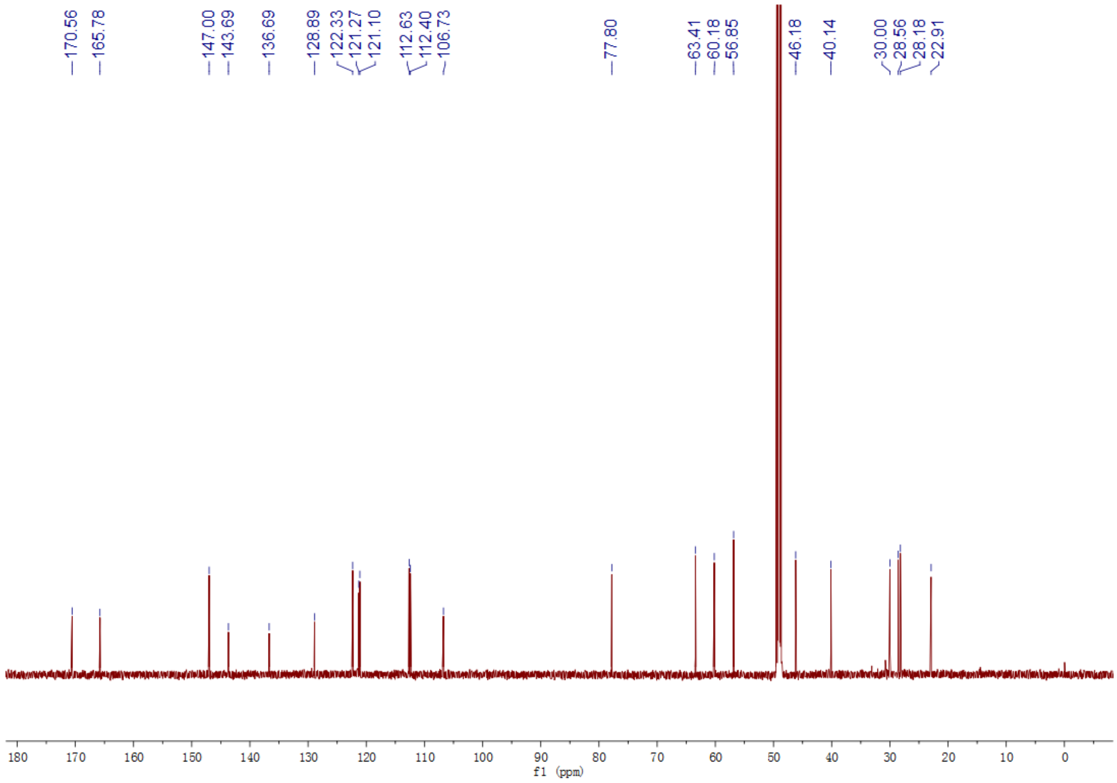


Figure S31 $^{13}$C NMR spectrum (600 MHz, methanol-$d_4$) of compound **5**

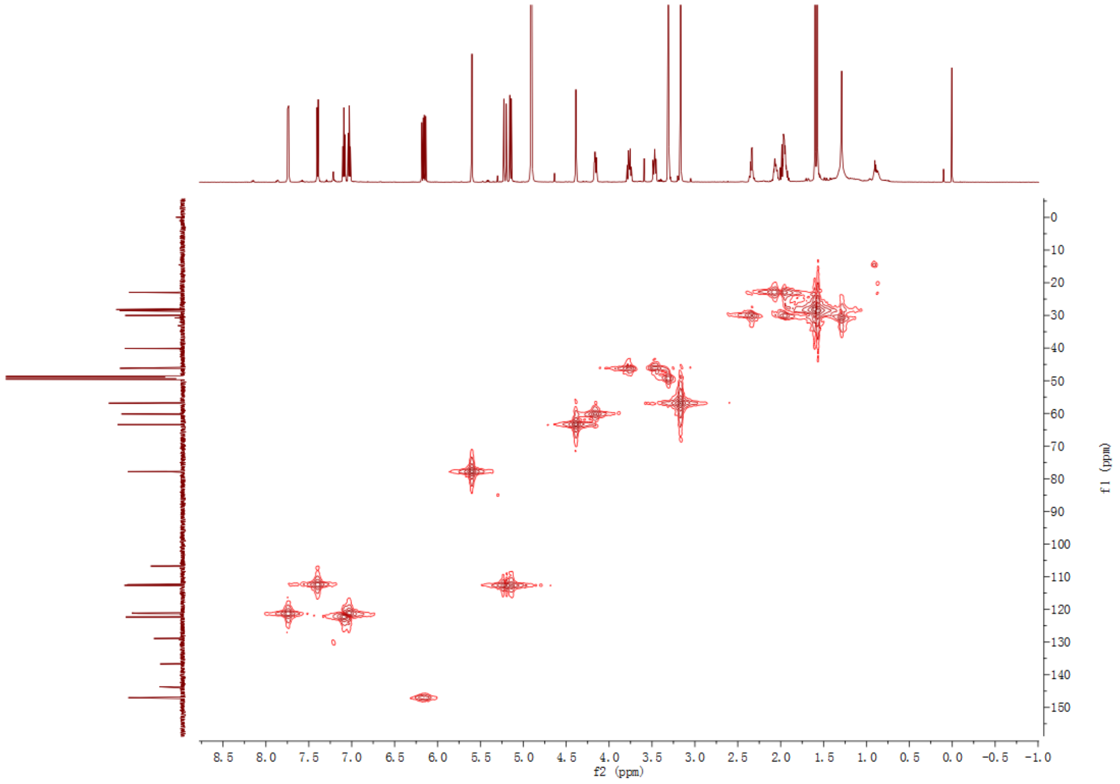


Figure S32 HMQC spectrum (600 MHz, methanol-*d*4) of compound **5**

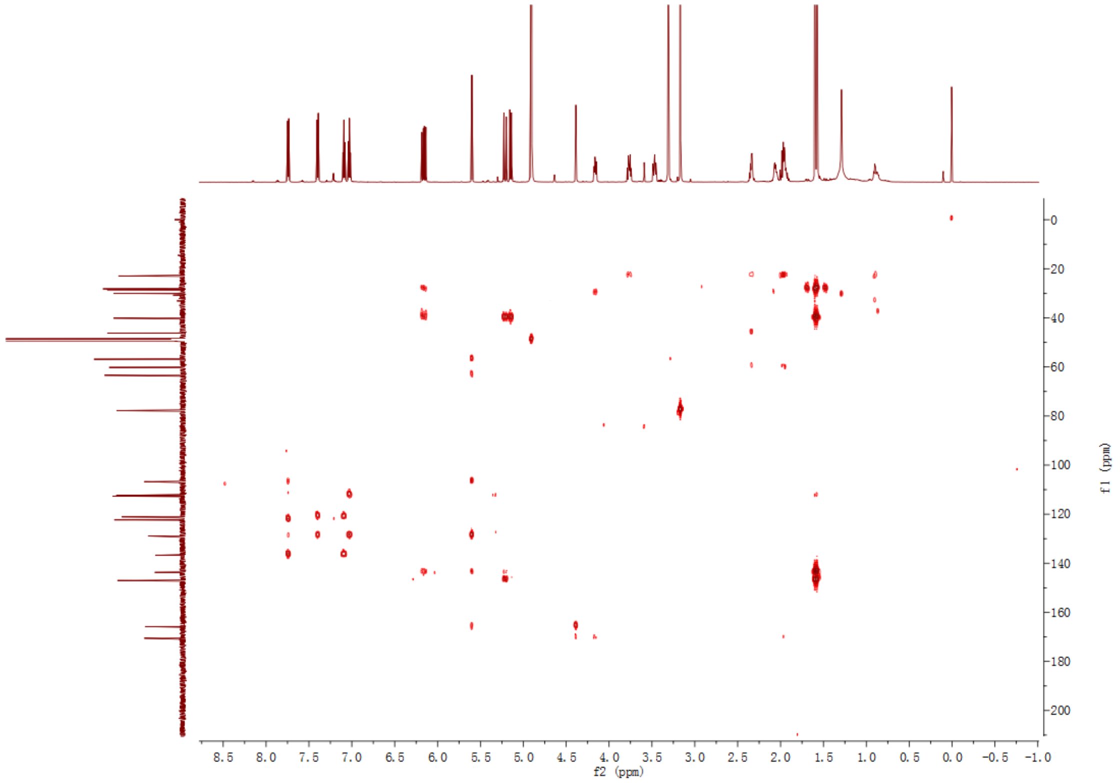


Figure S33 HMBC spectrum (600 MHz, methanol-$d_4$) of compound **5**

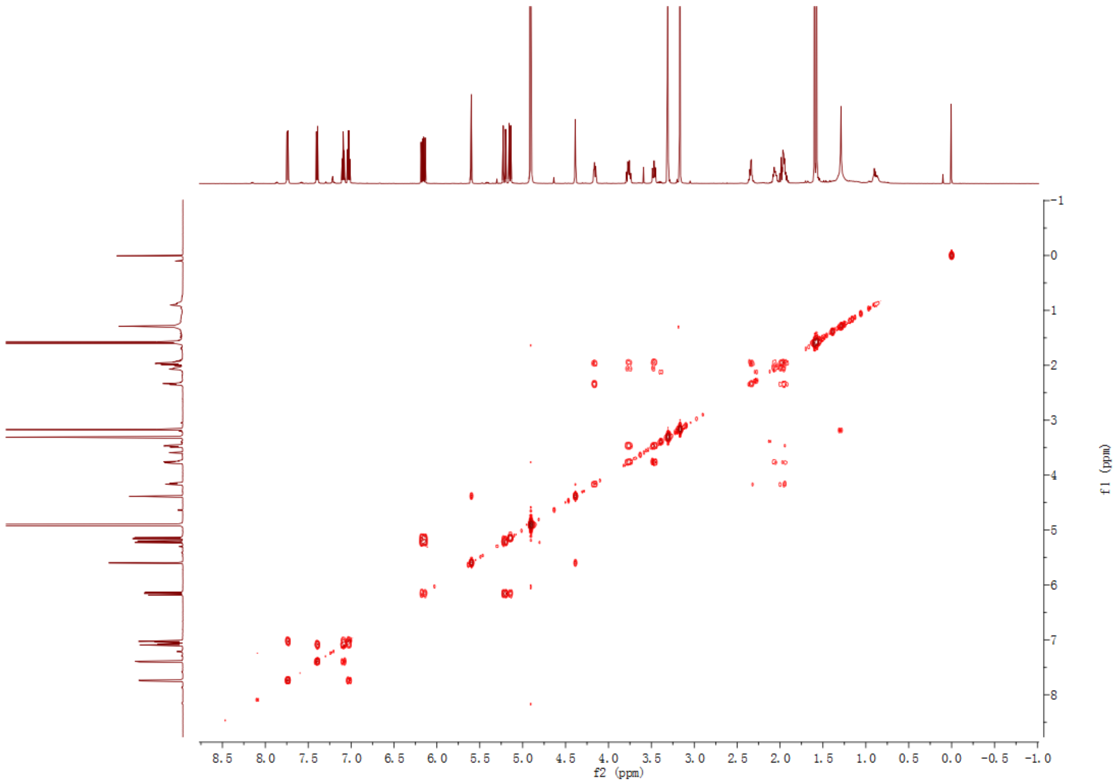


Figure S34 $^1H$-$^1H$ COSY spectrum (600 MHz, methanol-$d_4$) of compound **5**

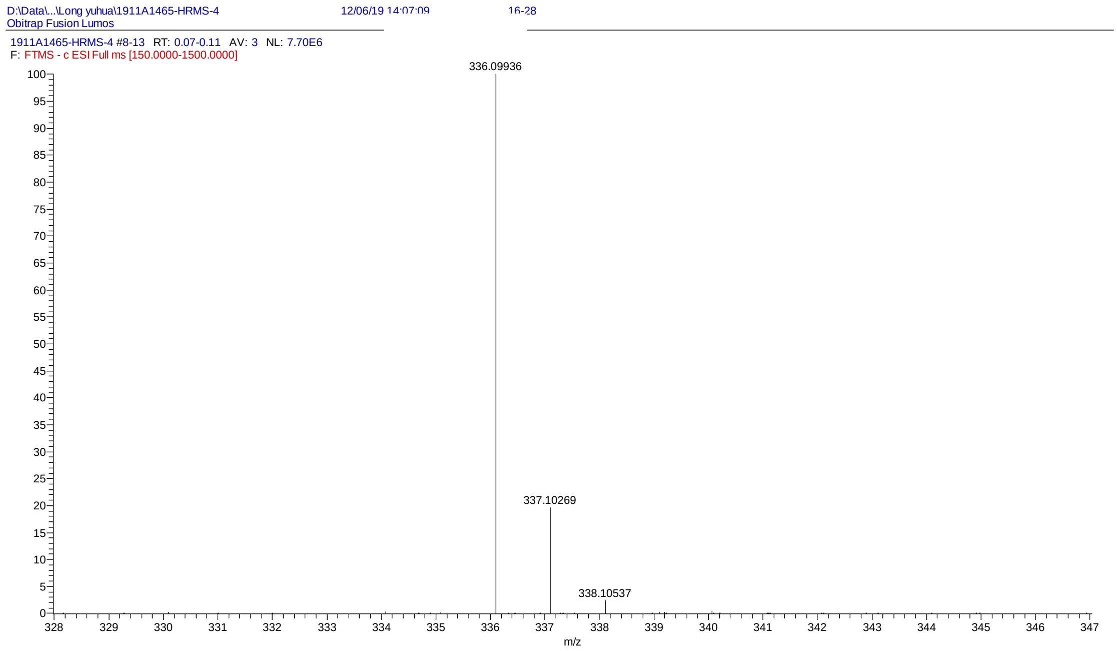


SPECTRUM - simulation :

| m/z | Theo. Mass | Delta (ppm) | RDB equiv. | Composition |
|---|---|---|---|---|
| 336.09936 | 336.09898 | 1.13 | 13.5 | $C_{18}$ $H_{14}$ $O_4$ $N_3$ |

Figure S35 HRESIMS of compound **6**

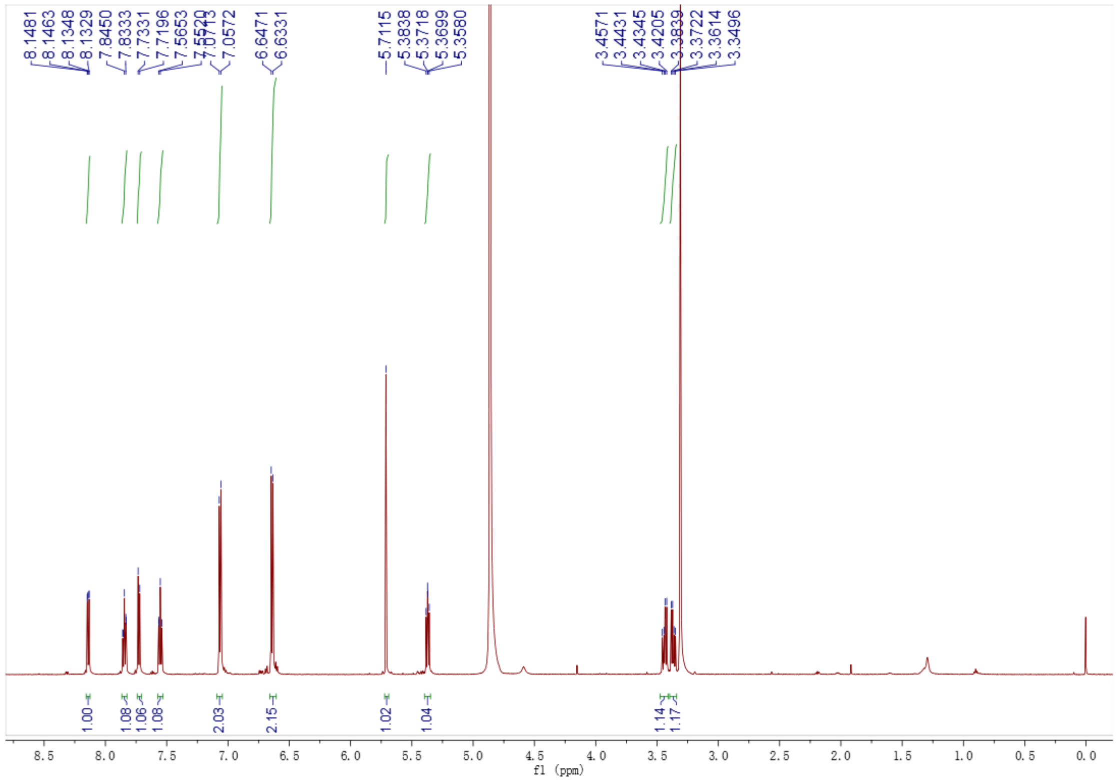


Figure S36 $^1$H NMR spectrum (600 MHz, methanol-$d_4$) of compound **6**

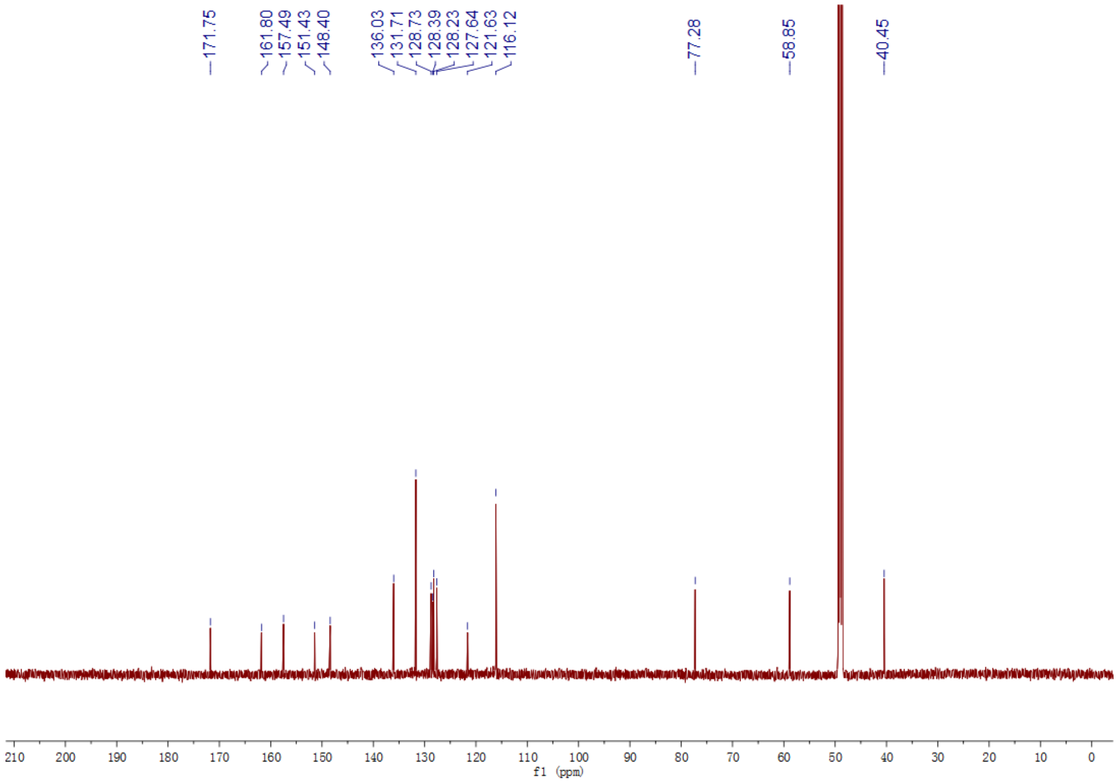


Figure S37 $^{13}$C NMR spectrum (600 MHz, methanol-$d_4$) of compound **6**

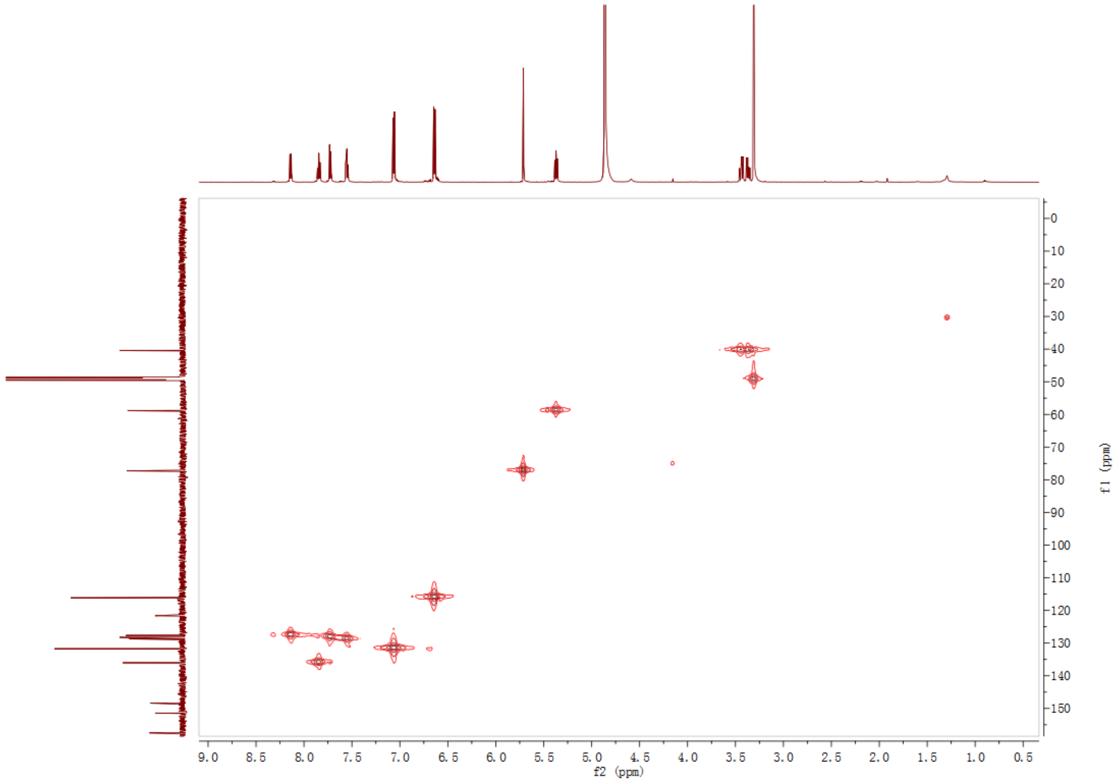


Figure S38 HMQC spectrum (600 MHz, methanol-$d_4$) of compound **6**

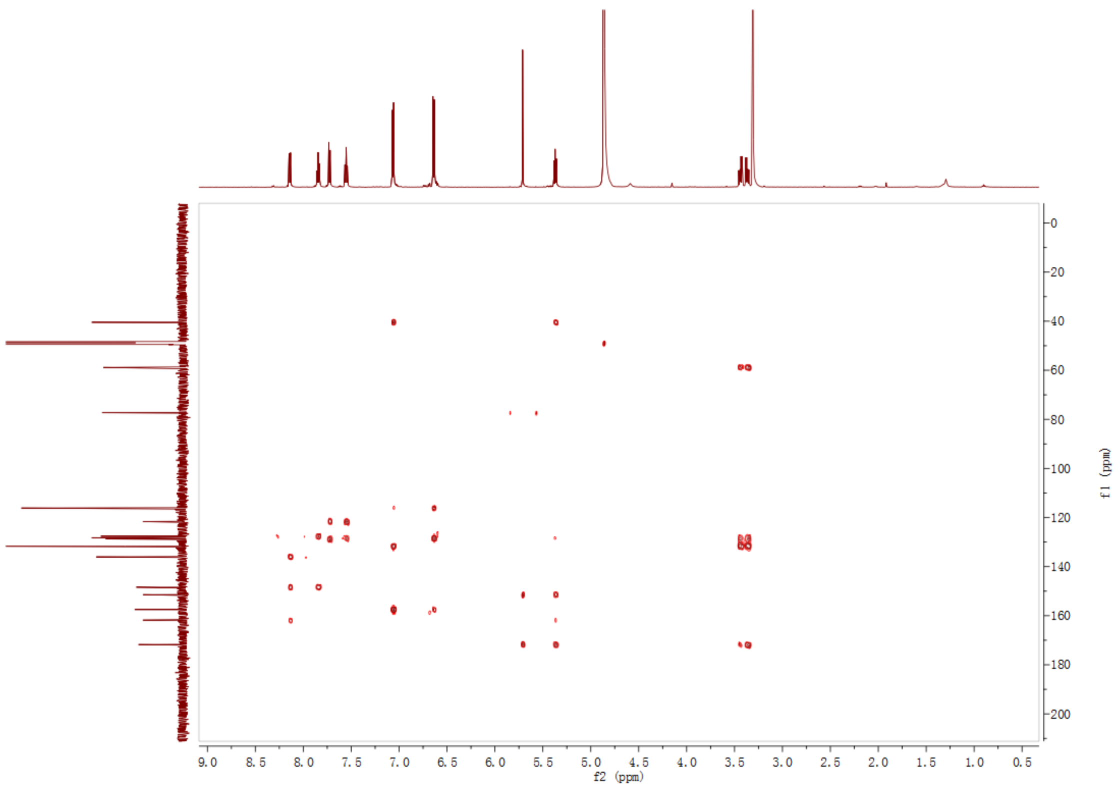


Figure S39 HMBC spectrum (600 MHz, methanol-$d_4$) of compound **6**

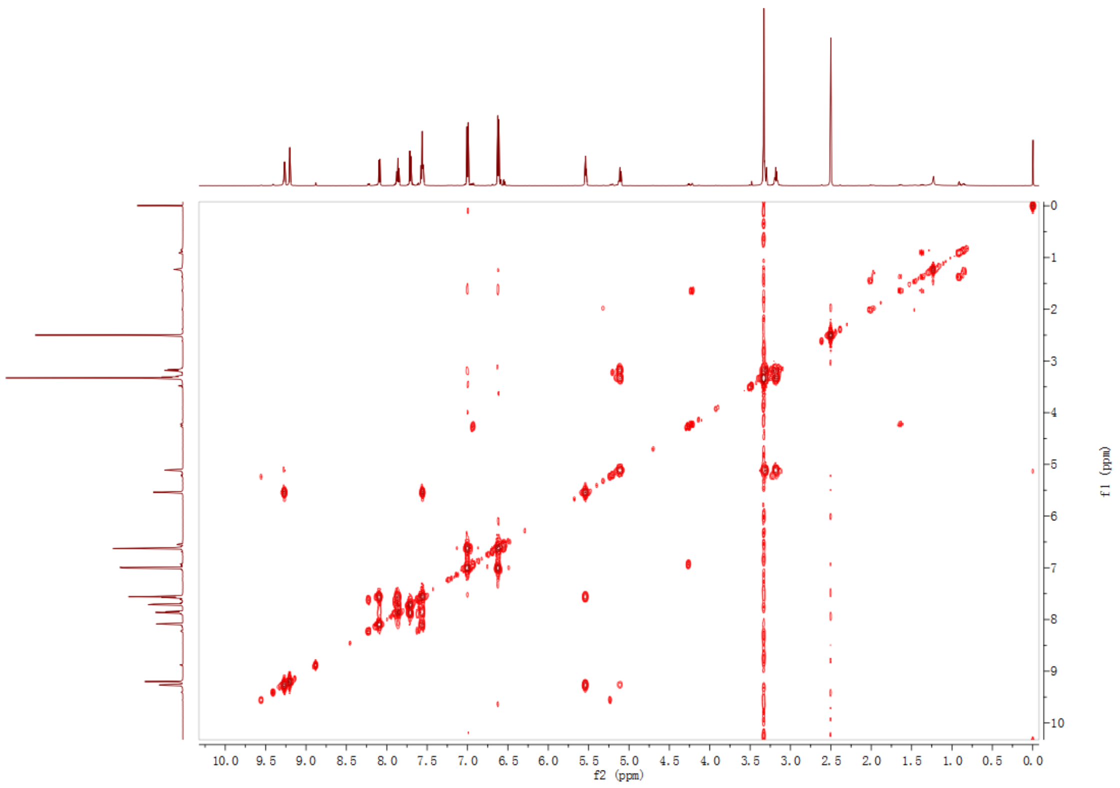


Figure S40 $^1$H-$^1$H COSY spectrum (600 MHz, methanol-$d_4$) of compound **6**

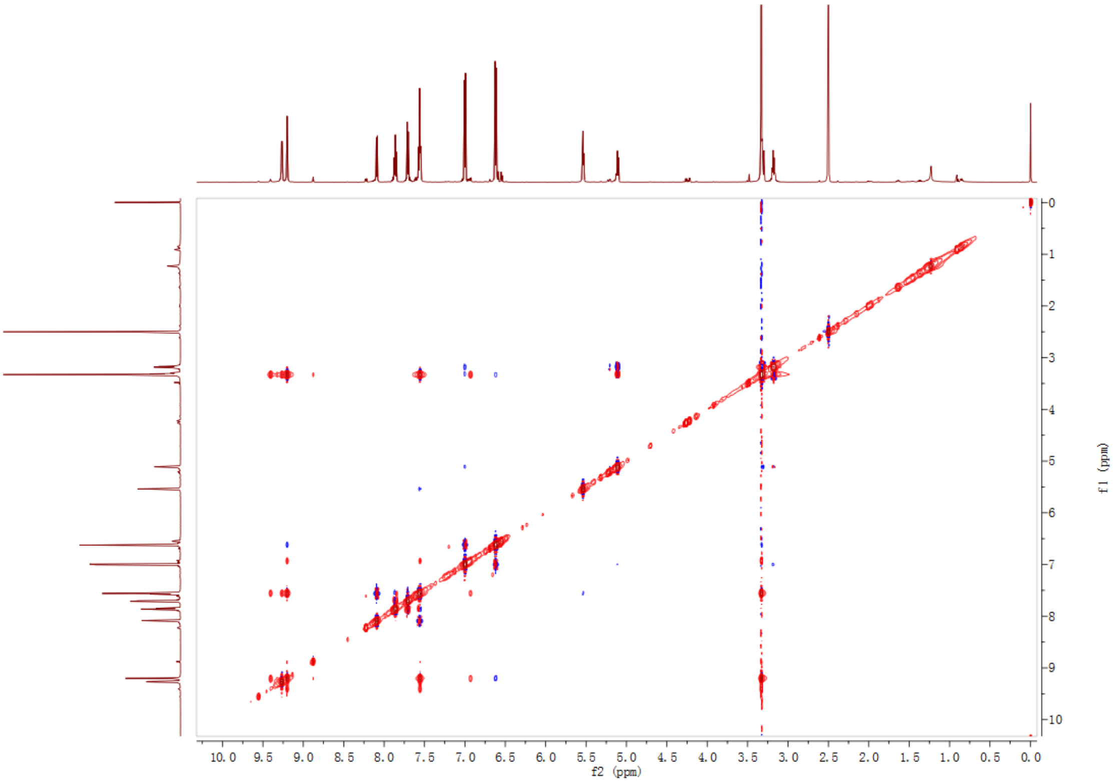

Figure S41 NOESY spectrum (600 MHz, DMSO-$d_6$) of compound **6**

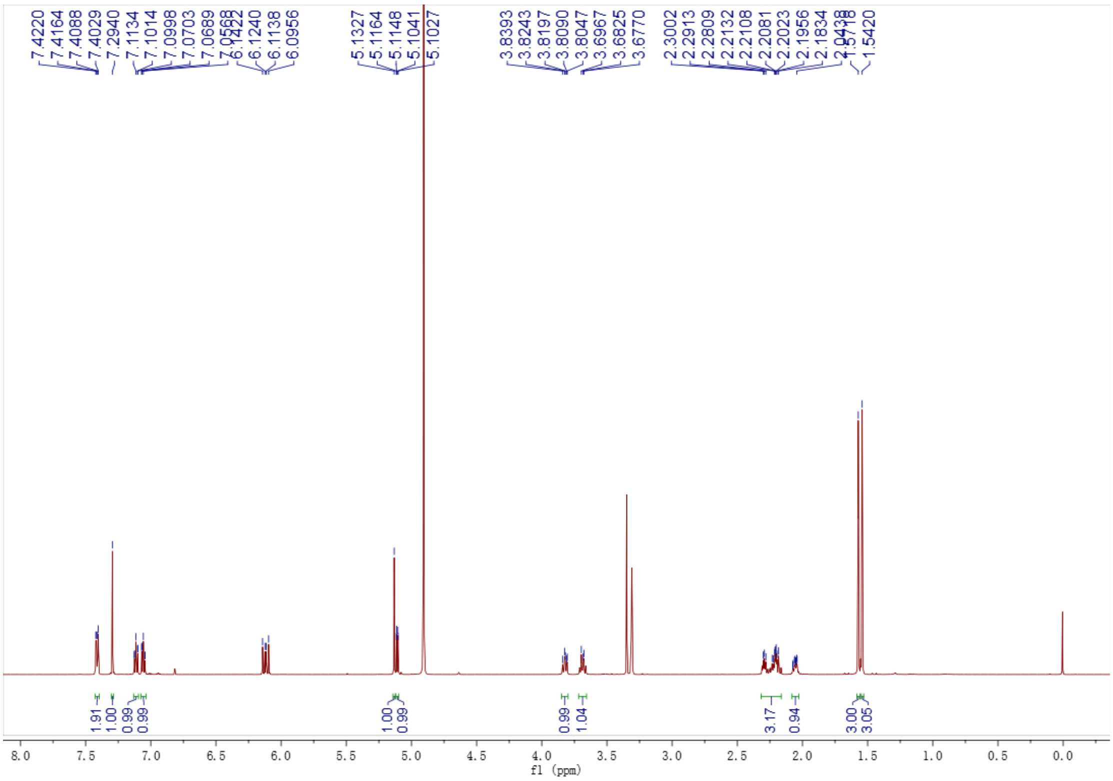

Figure S42 $^{1}$H NMR spectrum (600 MHz, methanol-$d_4$) of compound **7**

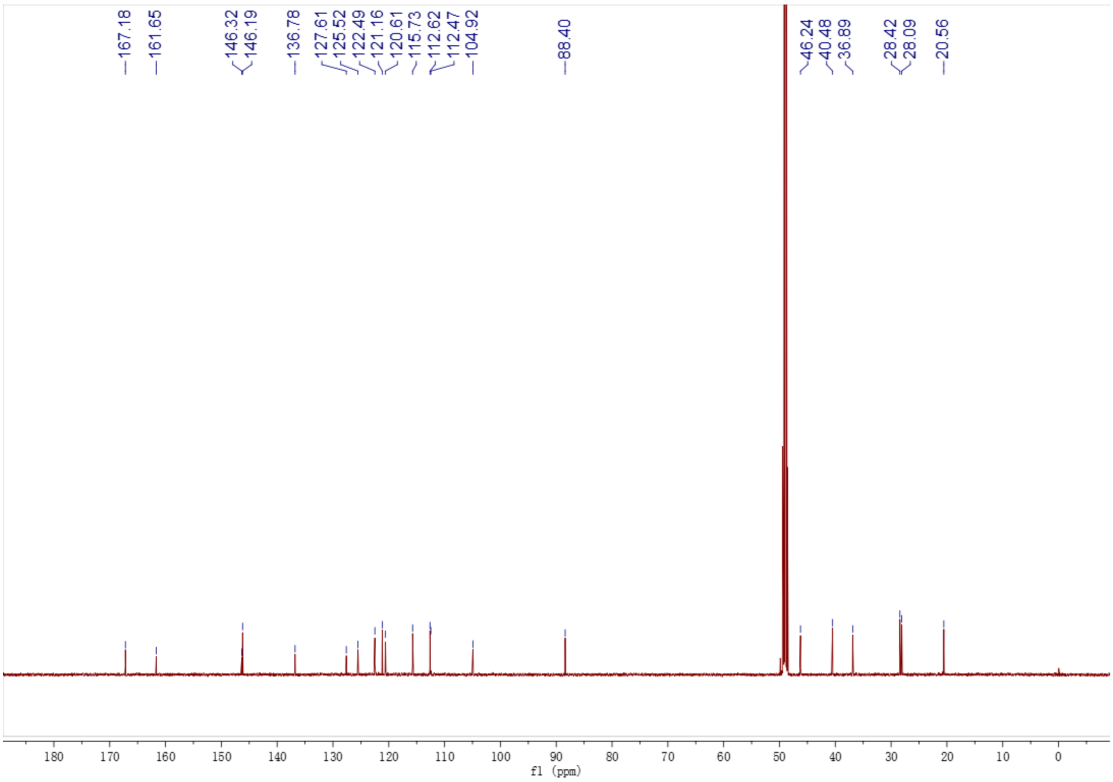

Figure S43 $^{13}$C NMR spectrum (600 MHz, methanol-$d_4$) of compound **7**

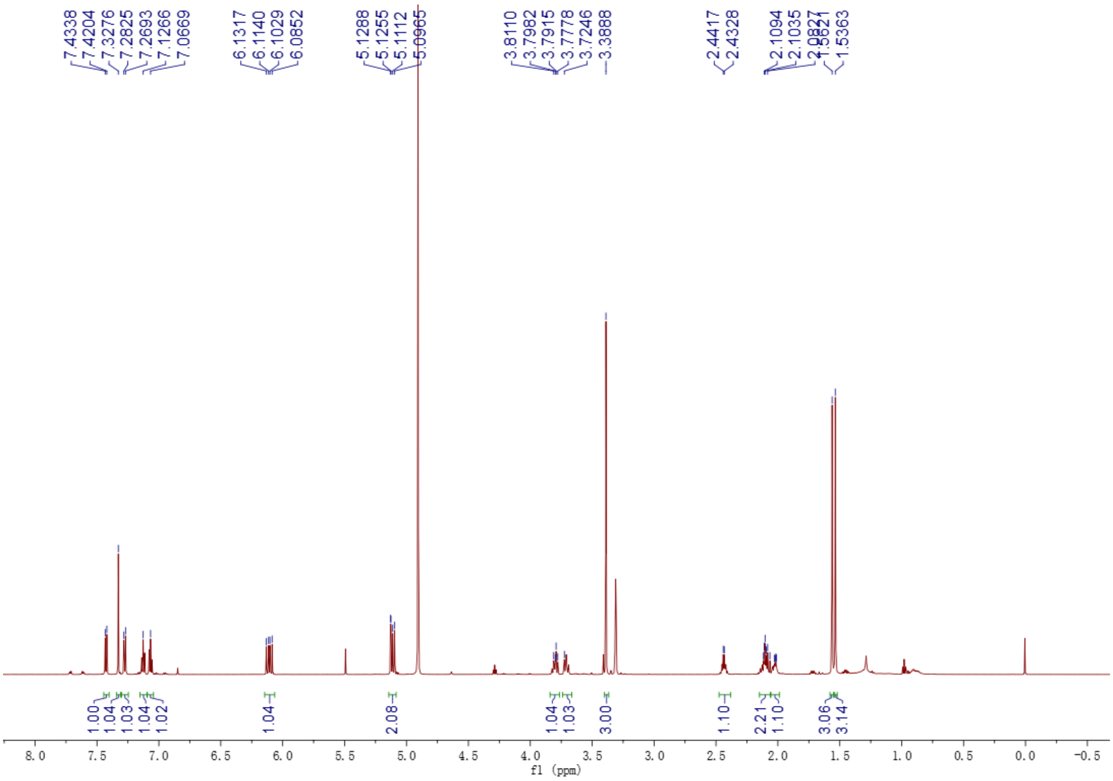

Figure S44 $^{1}$H NMR spectrum (600 MHz, methanol-$d_4$) of compound **8**

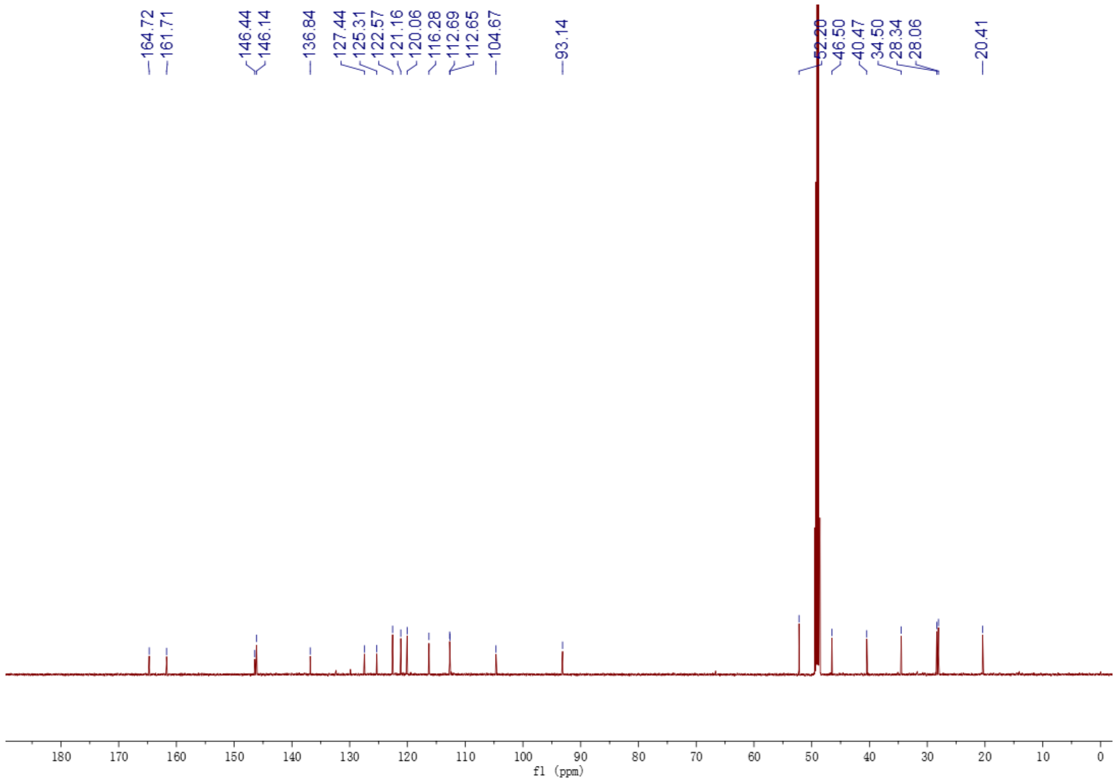

Figure S45 $^{13}$C NMR spectrum (600 MHz, methanol-$d_4$) of compound **8**

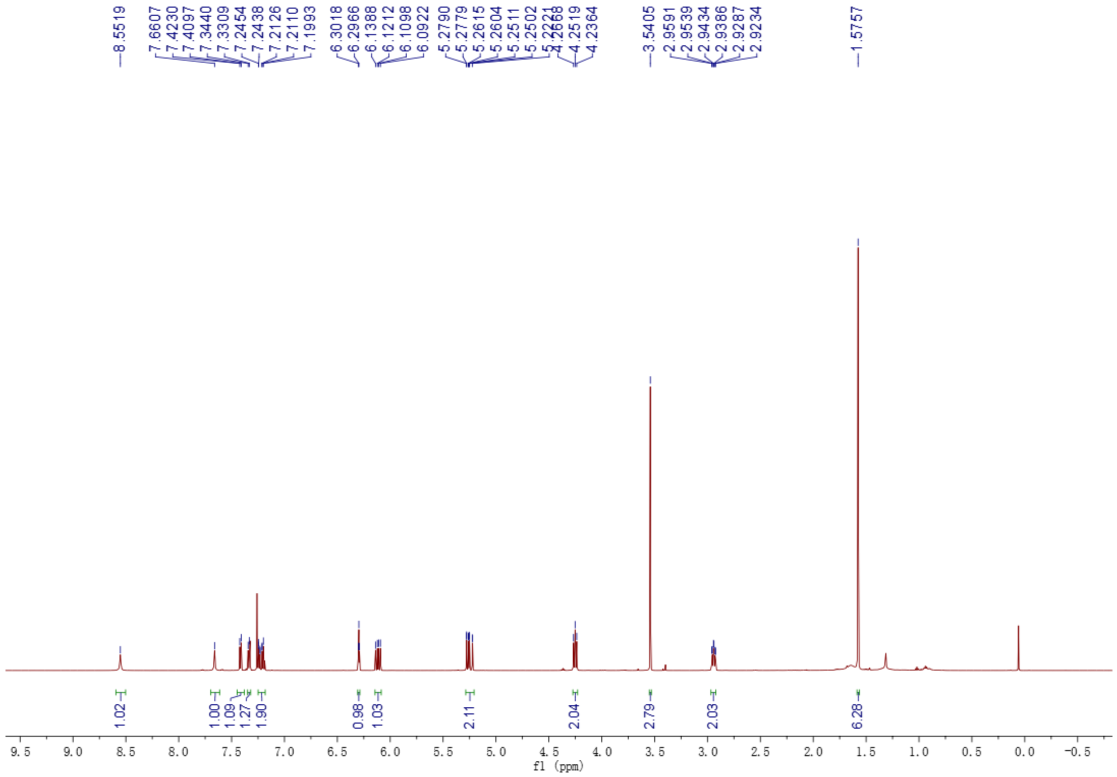

Figure S46 $^{1}$H NMR spectrum (600 MHz, chloroform-$d$) of compound **9**

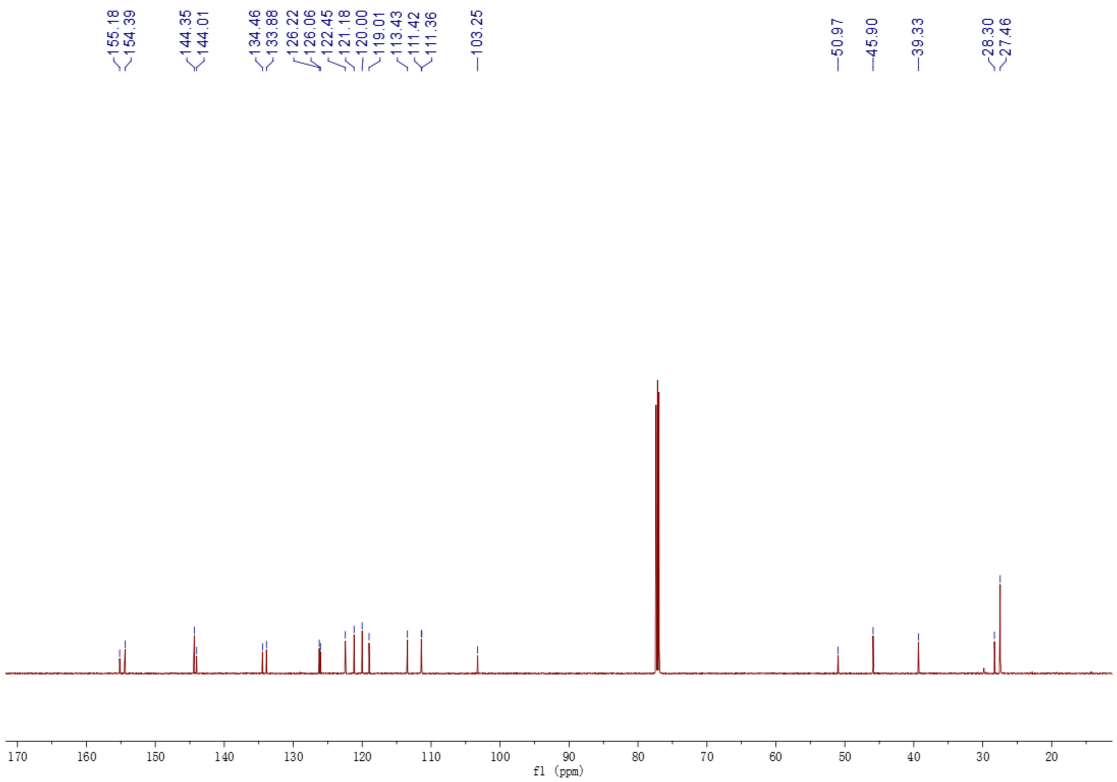

Figure S47 $^{13}C$ NMR spectrum (600 MHz, chloroform-*d*) of compound **9**

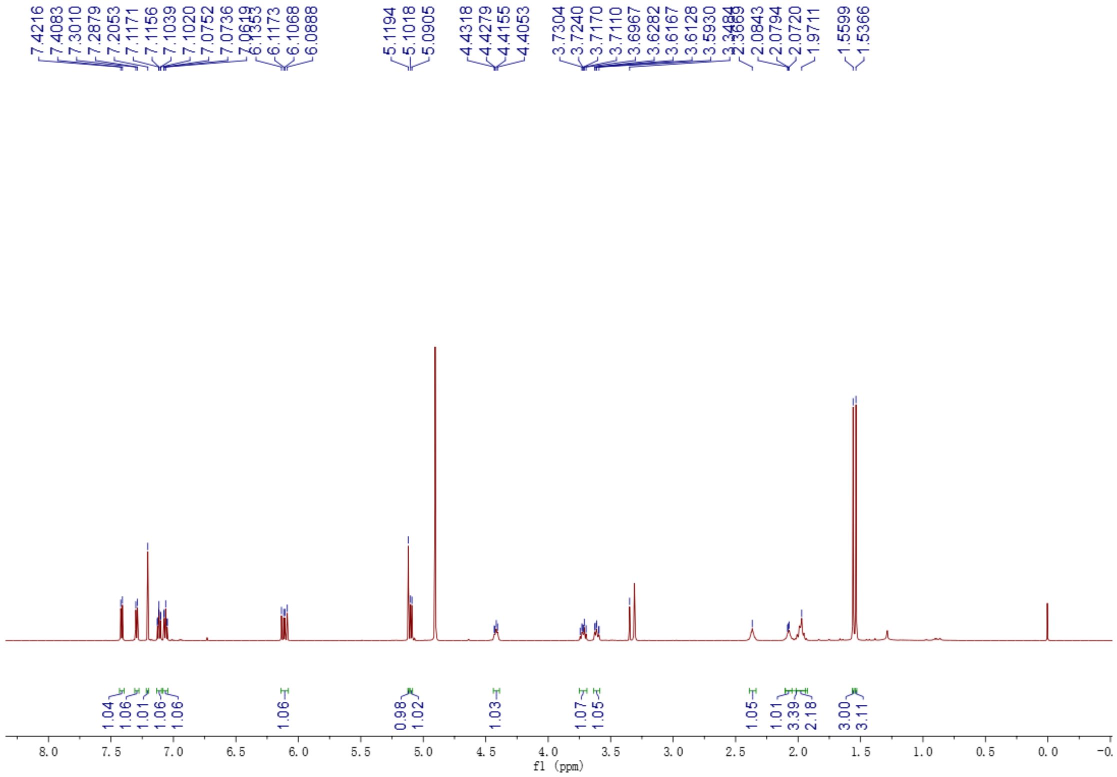

Figure S48 $^{1}H$ NMR spectrum (600 MHz, methanol-$d_4$) of compound **10**

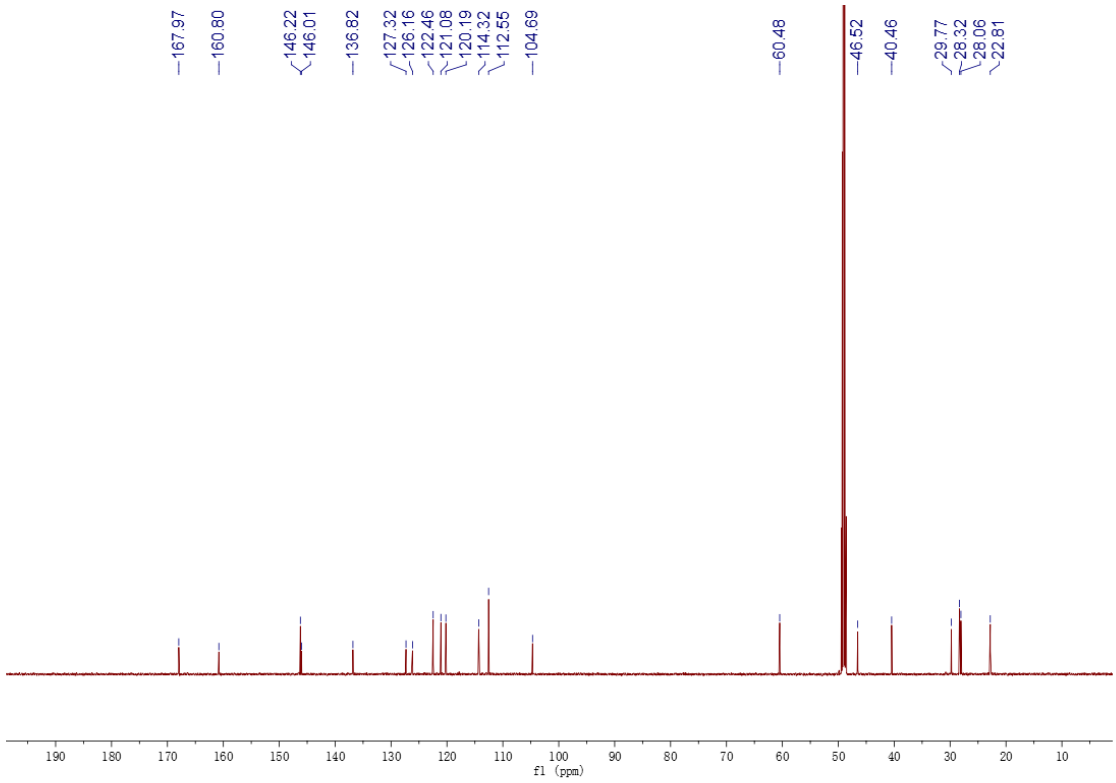


Figure S49 $^{13}C$ NMR spectrum (600 MHz, methanol-$d_4$) of compound **10**

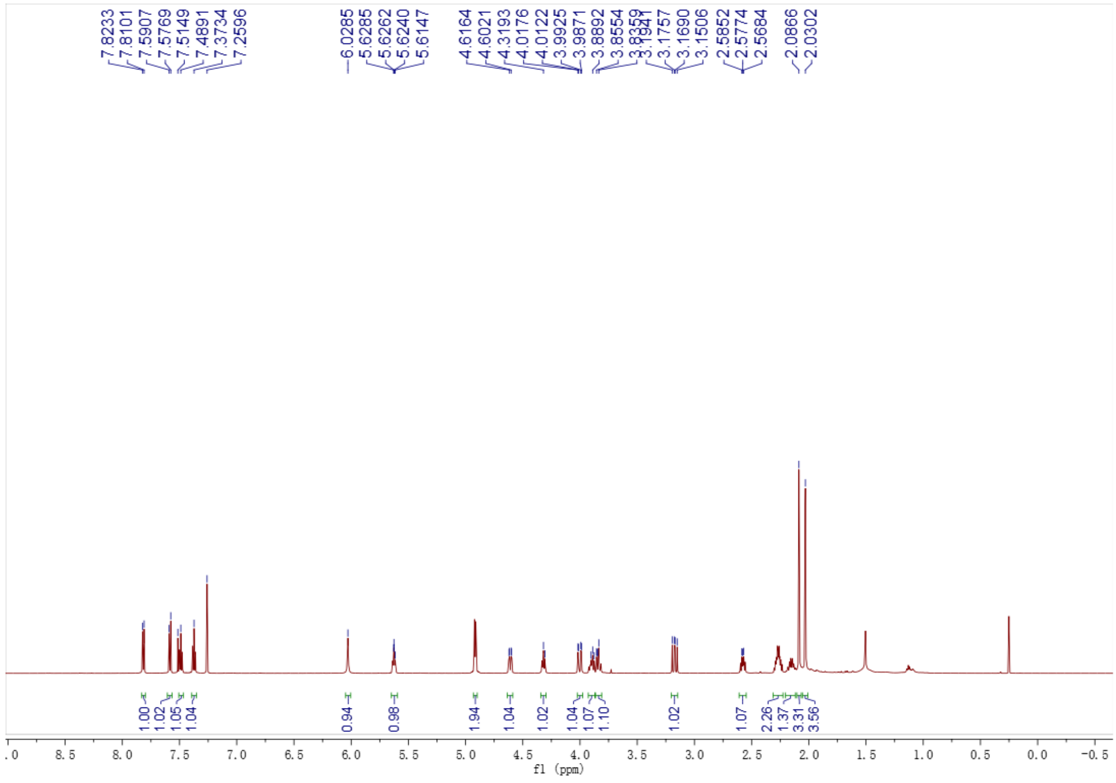


Figure S50 $^{1}H$ NMR spectrum (600 MHz, chloroform-$d$) of compound **11**

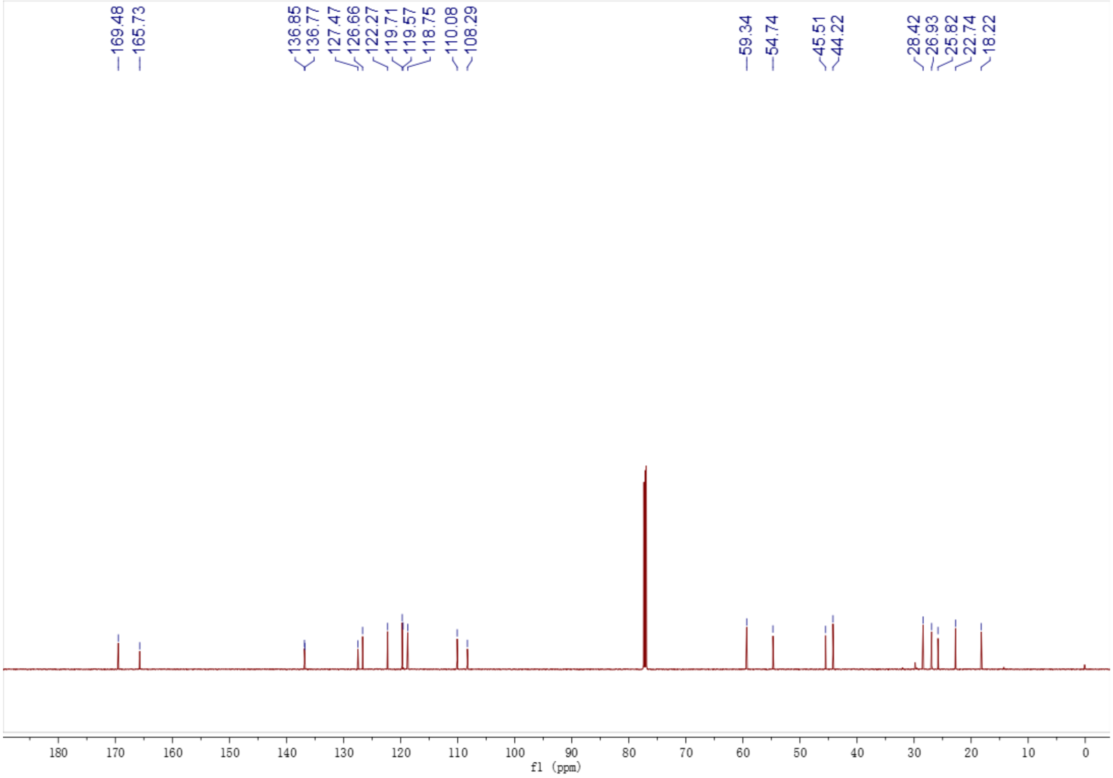

Figure S51 $^{13}$C NMR spectrum (600 MHz, chloroform-*d*) of compound **11**

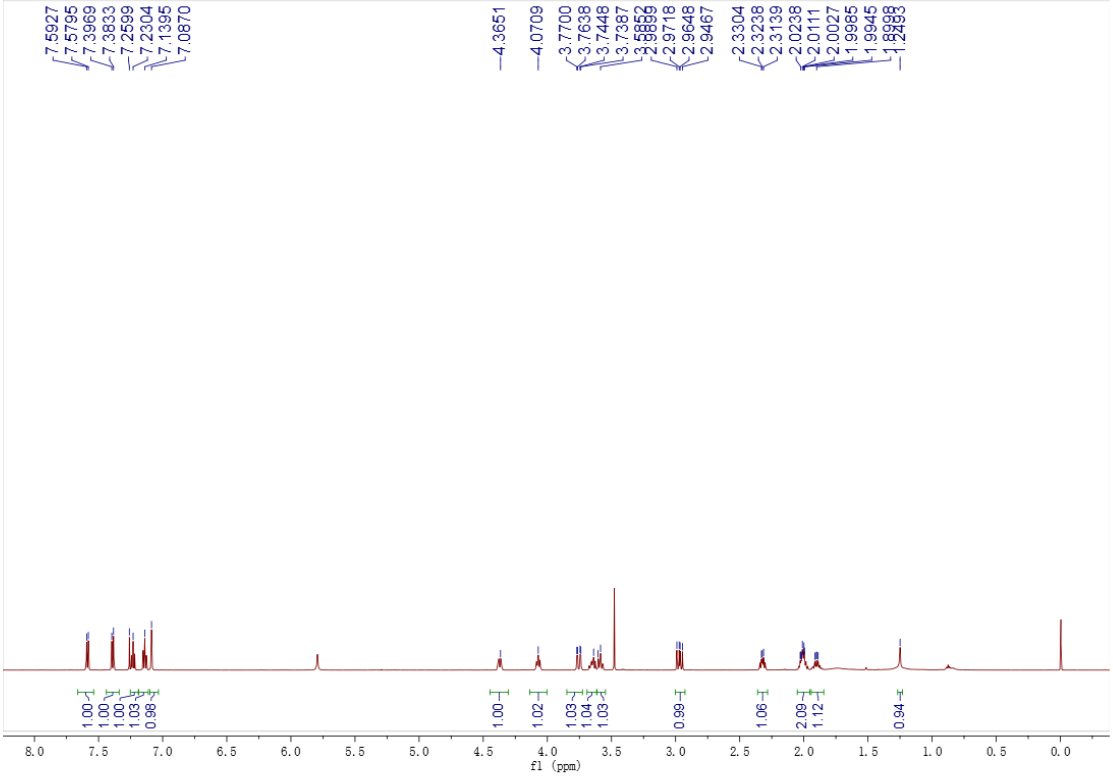

Figure S52 $^{1}$H NMR spectrum (600 MHz, chloroform-*d*) of compound **12**

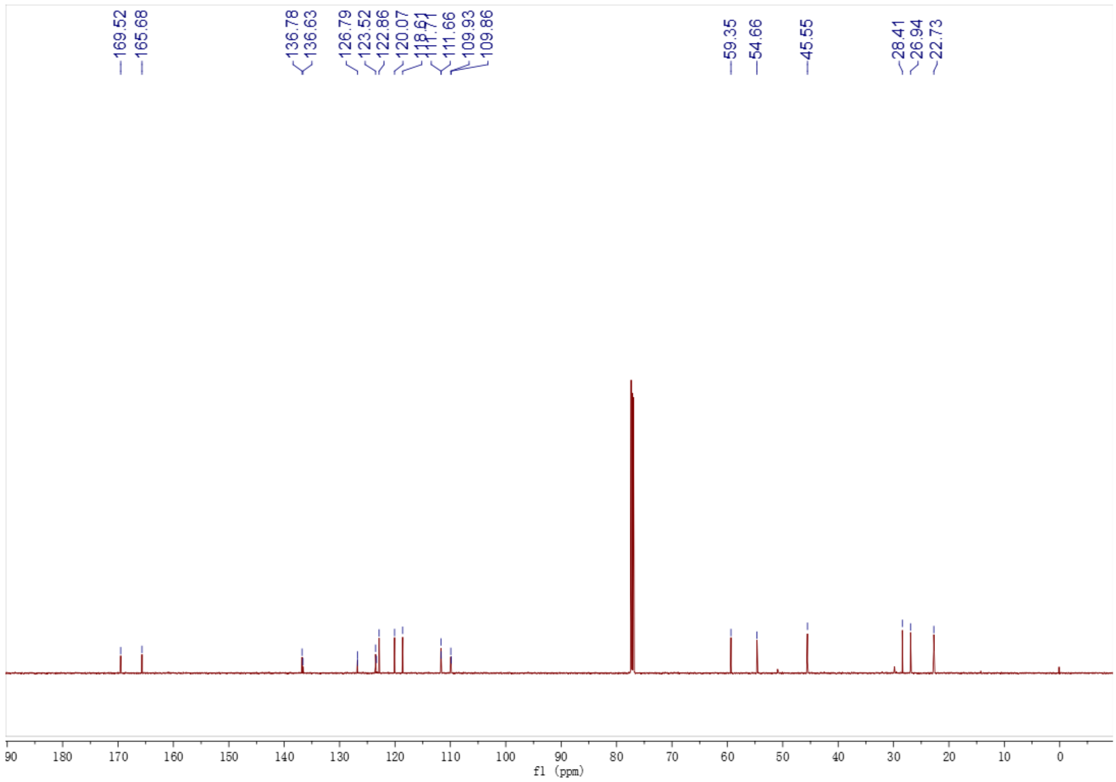

Figure S53 $^{13}$C NMR spectrum (600 MHz, chloroform-*d*) of compound **12**

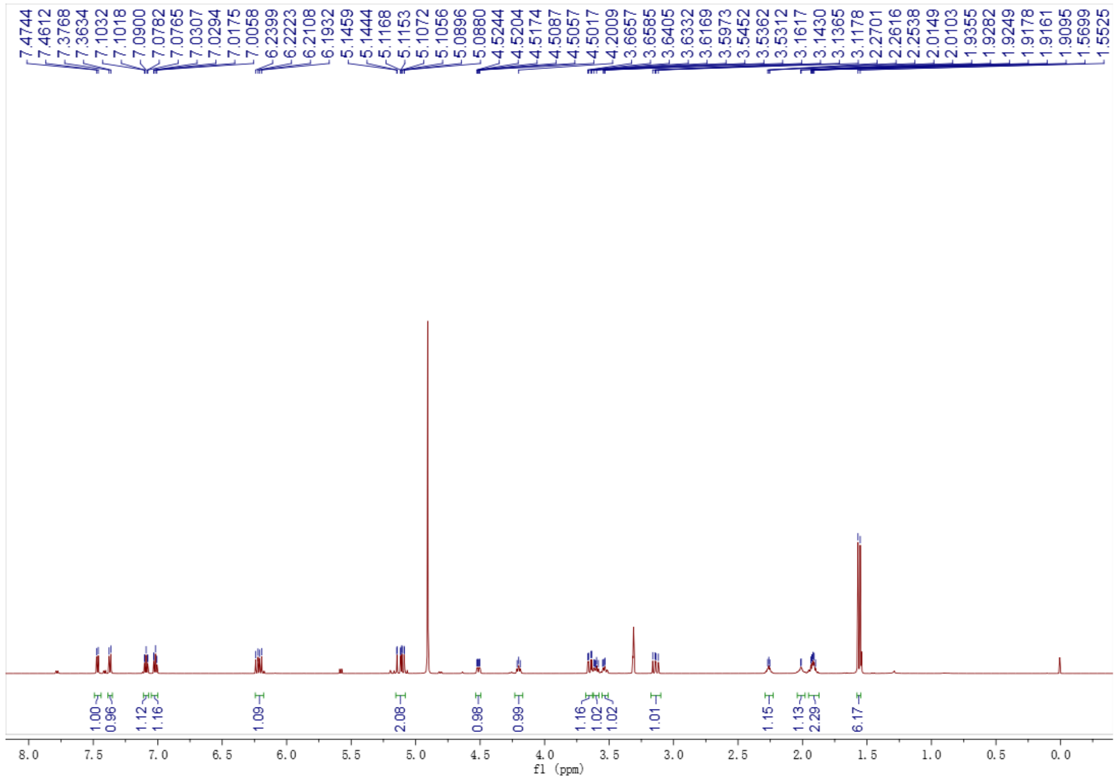

Figure S54 $^{1}$H NMR spectrum (600 MHz, methanol-$d_4$) of compound **13**

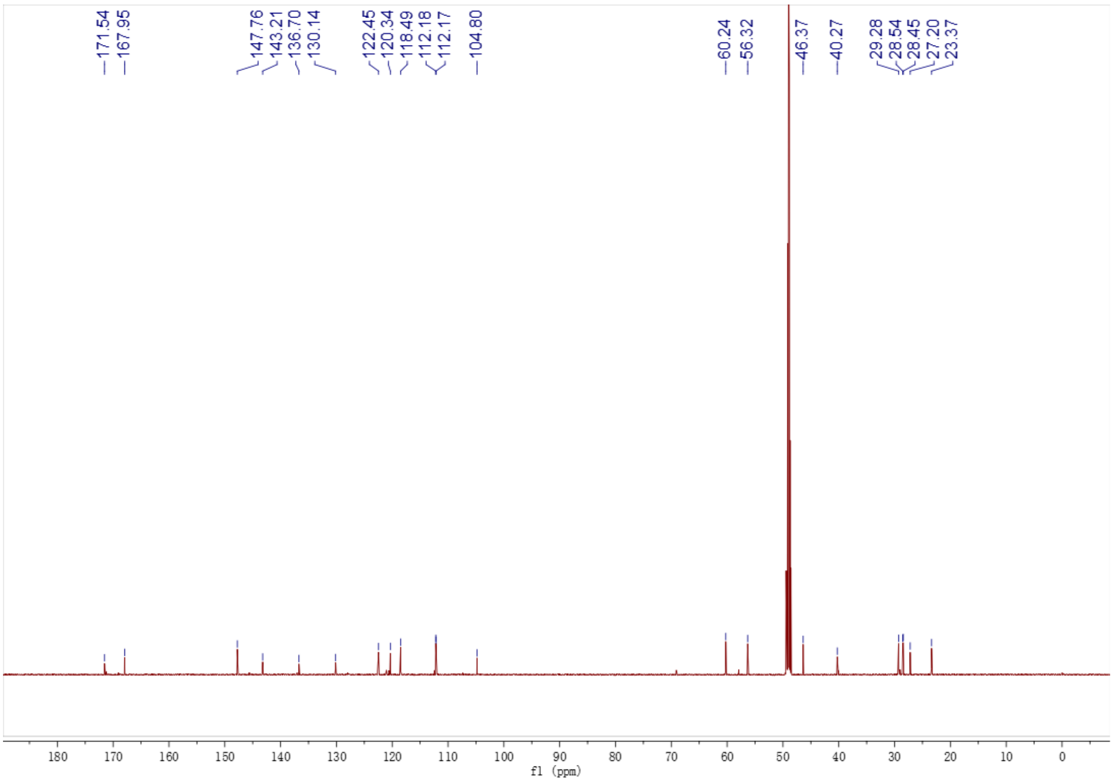

Figure S55 $^{13}$C NMR spectrum (600 MHz, methanol-$d_4$) of compound **13**

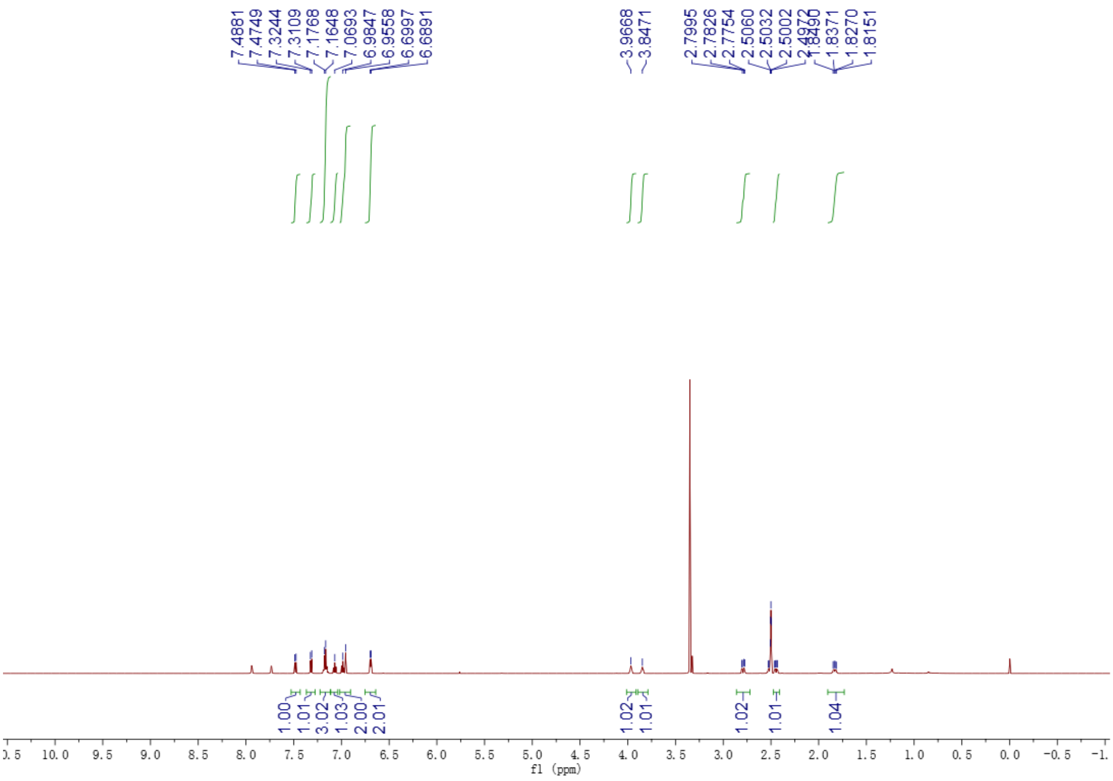

Figure S56 $^{1}$H NMR spectrum (600 MHz, DMSO-$d_6$) of compound **14**

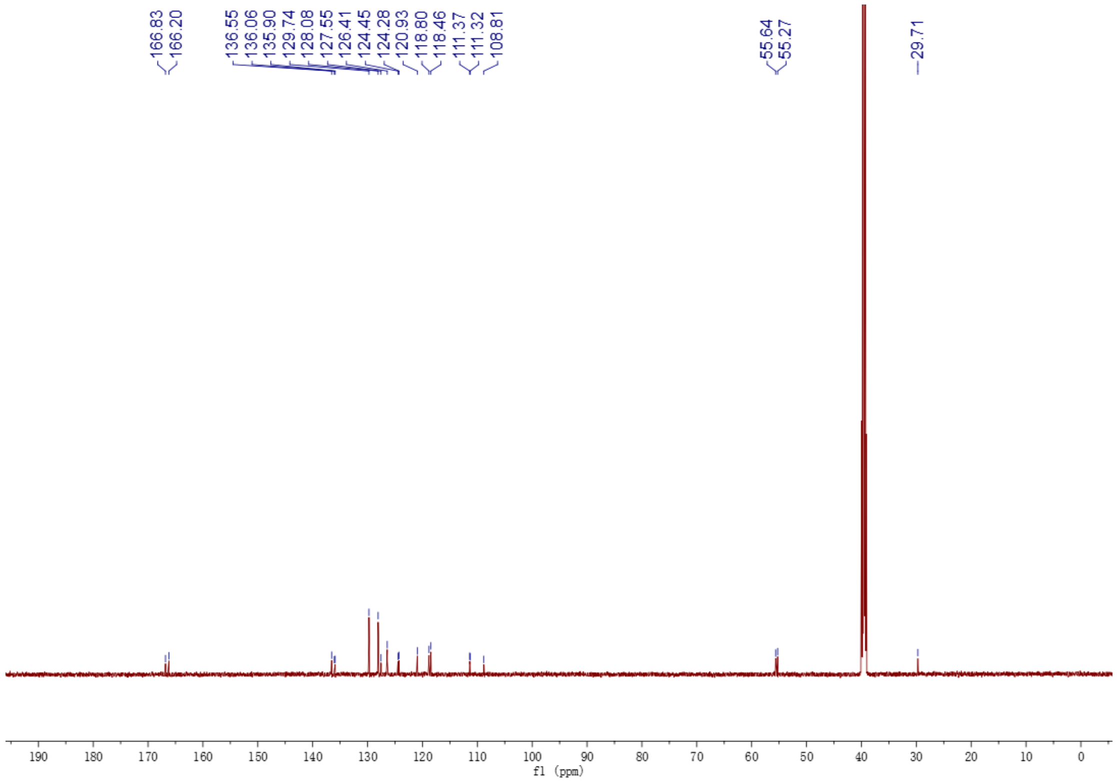

Figure S57 $^{13}$C NMR spectrum (600 MHz, DMSO-$d_4$) of compound **14**

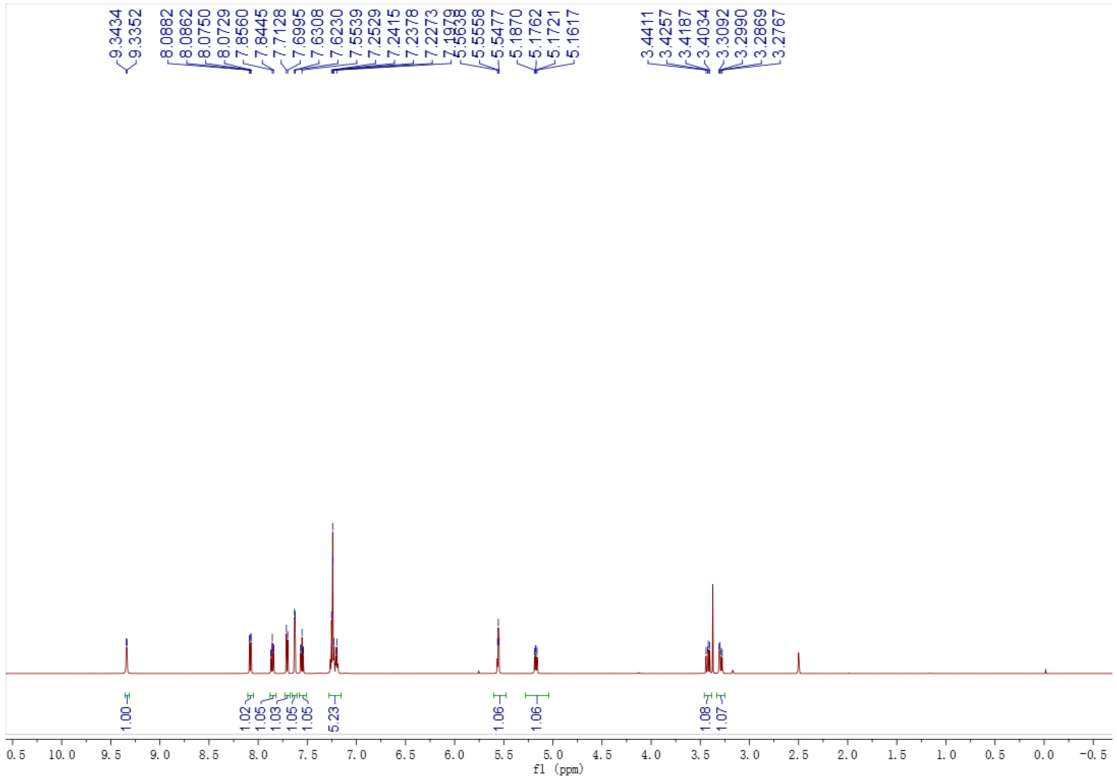

Figure S58 $^{1}$H NMR spectrum (600 MHz, DMSO-$d_6$) of compound **15**

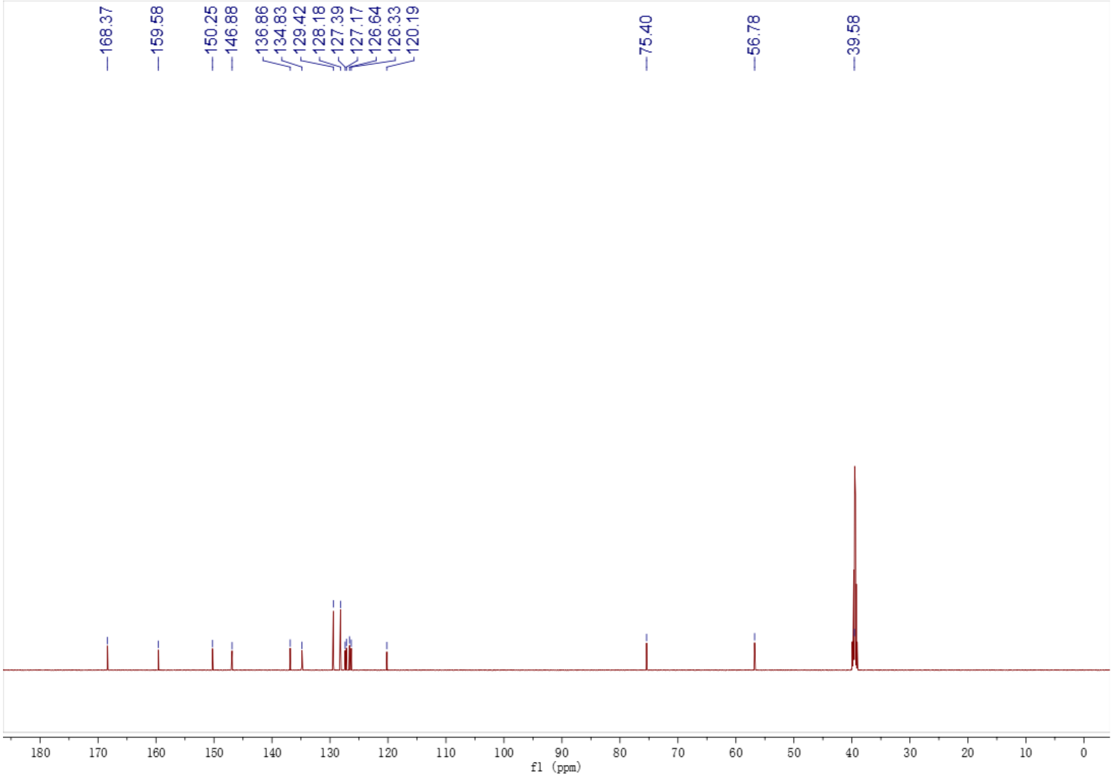

Figure S59 $^{13}C$ NMR spectrum (600 MHz, DMSO-$d_4$) of compound **15**

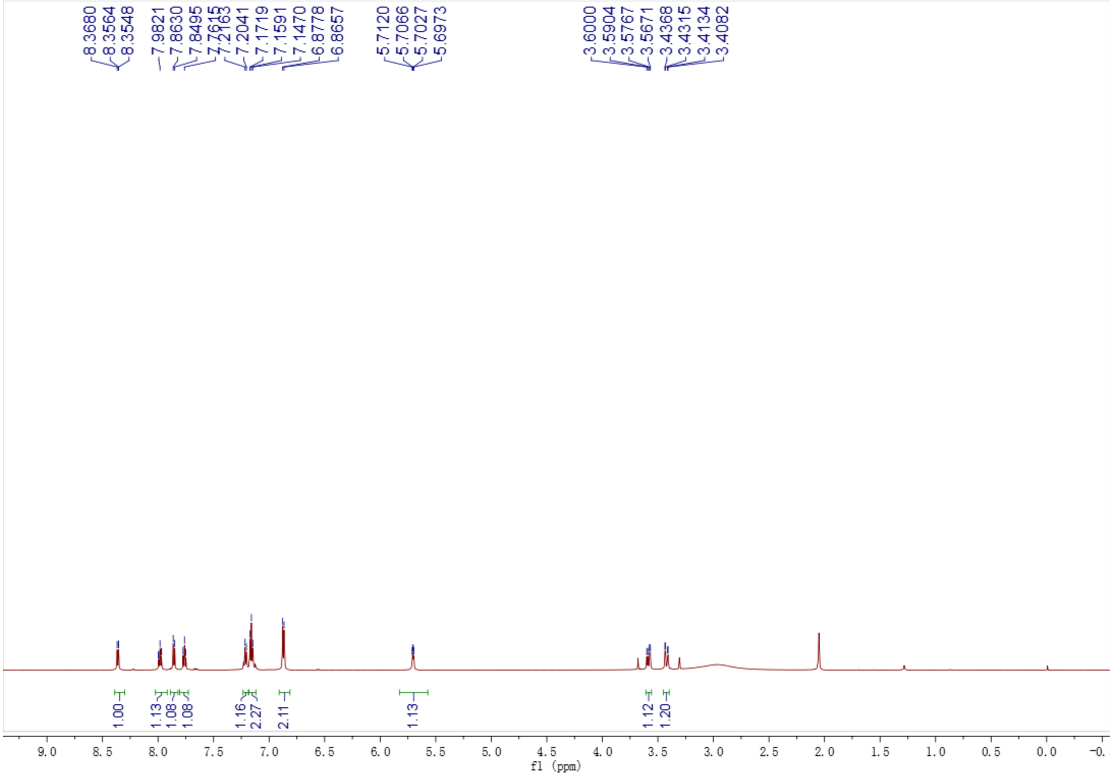

Figure S60 $^{1}H$ NMR spectrum (600 MHz, acetone-$d_6$) of compound **16**

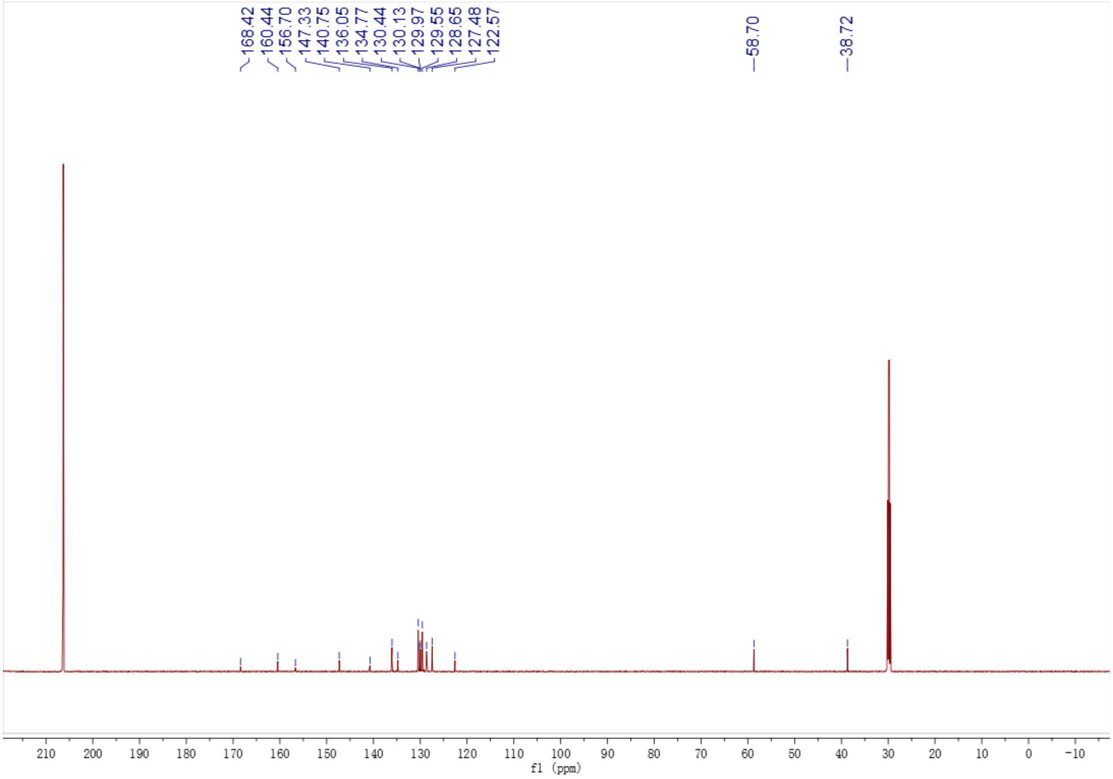


Figure S61 $^{13}$C NMR spectrum (600 MHz, acetone-$d_4$) of compound **16**
